# Supplementary material for: Patient-reported experiences and views on the Cytosponge test: a mixed-methods analysis from the BEST3 trial
Source: BMJ Open. 2022 Apr 7;12(4):e054258. doi: 10.1136/bmjopen-2021-054258 (PMC8990713; doi:10.1136/bmjopen-2021-054258)
Supplement: Supplementary data [file bmjopen-2021-054258supp001.pdf]

Patient-reported experiences and views on the Cytosponge test: a mixed-methods analysis from the BEST3 trial

Roberta Maroni*, Jessica Barnes*, Judith Offman*, Fiona Scheibl, Samuel G Smith, Irene Debiram-Beecham, Jo Waller, Peter D Sasieni, Rebecca C Fitzgerald, Greg Rubin, BEST3 Consortium, Fiona M Walter

*Roberta Maroni, Jessica Barnes and Judith Offman equally contributed to this paper.

SUPPLEMENTARY MATERIALS

METHODS

Study design

The BEST3 trial [1] was a randomised controlled trial set in primary care with a mixed design (site-level and patient-level randomised) that enrolled 13,222 participants aged 50 or over with acid reflux symptoms ongoing for more than six months, identified via their general practice medical records. The primary endpoint was to compare the rate of diagnosis of Barrett's oesophagus between those offered the Cytosponge-trefoil factor 3 (TFF3) test and those on current management, and the results showed a ten-fold increase in being diagnosed with Barrett's oesophagus in the intervention arm compared with usual care.

The invitation letter (intervention arm only) was accompanied by an information leaflet on the Cytosponge. Participants expressing interest in receiving the test received a further information sheet with more details on the study and the Cytosponge. On the day of the test, participants were asked not to eat or drink anything in the four hours before the appointment. The appointment was held at participants' general practices and attendees (N = 1750) were offered an anaesthetic throat spray (optional) and water to drink to help ingest the capsule, following which 1654 (95%) patients produced a successful swallow. Furthermore, those producing a successful swallow but receiving an 'inadequate' test result (i.e. low-confidence negative TFF3, equivocal, or processing/technical failure) were invited to a repeat appointment when local resources and capacity allowed for that. All patients with a positive TFF3 result were referred for a confirmatory endoscopy, which was necessary to establish a diagnosis of Barrett's oesophagus or oesophageal cancer.

Data collection

Qualitative analysis

Face-to-face interviews were conducted by Fiona Scheibl, BA (Hons) and PhD, working at the time as Research Associate for the Department of Public Health and Primary Care at the University of Cambridge. FS has undergraduate and postgraduate training in social research and has spent more than 15 years in social and health care research in several universities in the UK. No relationship between FS and the interviewees was established prior to study commencement and the participants had no knowledge of the researcher's goals, except for what was reported in the Patient Information Leaflet, the invitation letters or the further information sheet sent by the BEST3 team, which set out all the aims and terms of the research project. The interview questions were provided by the authors and were pilot tested. No field notes were collected. Transcriptions of the audio recordings of the interviews were not returned to participants for comments or correction.

Analysis

Quantitative analysis
Questionnaire scoring

- Gastro-oesophageal reflux disease Impact Scale [2]: answers to each item were converted to scores on a four-point ordinal scale (1 = 'Never', 2 = 'Sometimes', 3 = 'Often', 4 = 'Daily') and then averaged to obtain each participant's final score.

Maroni R, *et al. BMJ Open* 2022; 12:e054258. doi: 10.1136/bmjopen-2021-054258

- Shorter six-item form of the Spielberger State-Trait Anxiety Inventory (STAI-6) [3]: item scores on a four-point ordinal scale (1 = 'Not at all', 2 = 'Somewhat', 3 = 'Moderately', 4 = 'Very much') were reversed for positively worded questions and their sum was scaled so that the total score ranged from 20 to 80, as per the STAI guidelines.
- Perceived risk [5]: both risk compared to someone of the same age ('Much lower', 'Lower', 'Neither higher nor lower', 'Higher', 'Much higher') and absolute risk in a lifetime ('0%', '5%', '10%', '25%', '50%', '75%', '100%') are shown with some of the answer categories combined.
- Inventory to Assess Patient Satisfaction (IAPS) [6]: ratings categories ('Strongly agree', 'Agree', 'Not sure', 'Disagree', 'Strongly disagree') were combined ('Agree', 'Not sure', 'Disagree') and number and proportion of participants for each item are presented. For presentation purposes (Table 2), the text of items referring to negative aspects of the patient experience was rephrased using negative constructs to facilitate the visual comparison between items. Answers to the three items in the category "Pulling of the Cytosponge" were converted to scores on a 5-point ordinal scale (1 = 'Strongly agree', 2 = 'Agree', 3 = 'Not sure', 4 = 'Disagree', 5 = 'Strongly disagree'), which were then reversed for the two items referring to negative aspects. The three scores were then averaged for each patient to identify participants dissatisfied with the Cytosponge retrieval (i.e. score of 4 or above). Their ratings were cross-checked with the two inventory items referring to willingness to have the procedure again or to recommend it to friends.

Qualitative analysis

Researcher JB performed a thematic analysis on the interview data, with input from FW and JW. Data were organised and managed according to the Framework approach.[7] After familiarisation with the data through reading all the transcripts, JB developed an initial thematic framework of data labels. The aim with producing the initial set of labels was to enable effective data sorting and management – not to arrive at an exhaustive set of themes. This involved identifying an initial, broad set of labels that would be used to label and sort the data to enable the subsequent thematic analysis.

Labels were created inductively and deductively. Inductively-created labels were based on emergent concepts identified in the data. Deductively-created labels were based on the IAPS (as used in the quantitative questionnaire for the trial) [6] and the Theoretical Framework of Acceptability (TFA).[8] Use of the IAPS constructs as labels allowed us to more directly relate participant experience across qualitative and quantitative datasets. Use of the TFA constructs allowed us to examine additional dimensions of patient experience associated with acceptability that were not captured by IAPS.

Labels were discussed and reviewed by JB, FW and JW. JB then sorted the data by reading through each transcript and applying the labels cross-sectionally (i.e. the set of labels was applied across the entire set of transcripts where relevant). The labelled data was then transposed into the conventional Framework matrix, in which each label becomes a column and each participant/case becomes a row.

REFERENCES

1. Fitzgerald RC, di Pietro M, O'Donovan M, et al. Cytosponge-trefoil factor 3 versus usual care to identify Barrett's oesophagus in a primary care setting: a multicentre, pragmatic, randomised controlled trial. *Lancet* 2020;396(10247):333-44. doi: 10.1016/s0140-6736(20)31099-0.
2. Jones R, Coyne K, Wiklund I. The gastro-oesophageal reflux disease impact scale: a patient management tool for primary care. *Aliment Pharmacol Ther* 2007;25(12):1451-9. doi: 10.1111/j.1365-2036.2007.03343.x.
3. Marteau TM, Bekker H. The development of a six-item short-form of the state scale of the Spielberger State-Trait Anxiety Inventory (STAI). *Br J Clin Psychol* 1992;31(3):301-6. doi: 10.1111/j.2044-8260.1992.tb00997.x.
4. Kadri SR, Lao-Sirieix P, O'Donovan M, et al. Acceptability and accuracy of a non-endoscopic screening test for Barrett's oesophagus in primary care: cohort study. *BMJ* 2010;341:c4372. doi: 10.1136/bmj.c4372.
5. Lerman C, Trock B, Rimer BK, et al. Psychological side effects of breast cancer screening. *Health Psychol* 1991;10(4):259-67. doi: 10.1037//0278-6133.10.4.259.
6. Schoen RE, Weissfeld JL, Bowen NJ, et al. Patient satisfaction with screening flexible sigmoidoscopy. *Arch Intern Med* 2000;160(12):1790-6. doi: 10.1001/archinte.160.12.1790.

7. Ritchie J, Lewis C, McNaughton Nicholls C, et al. Qualitative Research Practice (Second Edition). London, UK: SAGE Publications Ltd. 2014.
8. Sekhon M, Cartwright M, Francis JJ. Acceptability of healthcare interventions: an overview of reviews and development of a theoretical framework. *BMC Health Services Research* 2017;17(1):88. doi: 10.1186/s12913-017-2031-8.

SUPPLEMENTARY TABLES

Supplementary Table 1. Sampling characteristics* of BEST3 participants being interviewed.

	**No. of participants (N = 30)**
**Geographic region in England**	
East	20
North-east	8
West	2
**Cytosponge-TFF3 outcome (at first appointment)**	
TFF3 negative	10
TFF3 positive	10
Inadequate (equivocal/low-confidence negative/technical or processing failure)**	6
Unsuccessful swallow	4
**Visual analogue scale acceptability rating (0-10)*** **	
5	2
6	1
7	1
8	4
9	4
10	9
Missing	9

*Also refer to Table 1 for the other sampling characteristics: age group and sex.
**Participants with an inadequate test result were invited to a repeat appointment when local resources and availability allowed for that.
***Visual analogue scale ratings were not used to ensure equal sampling of interviewees across scores, but rather to guarantee a diversity of experiences.

Supplementary Table 2. Understanding of test results, summarised by themes and quotes from patient interviews.

**Cytosponge test result**	**Theme**	**Exemplar quotes**
Positive	<u>Sense of shock due to expecting negative result</u><br>Some participants experienced shock as they had expected a negative result based on their understanding of cancer in general (i.e. that it is caused by lifestyle factors such as drinking or smoking, or that it is hereditary) rather than an understanding of Barrett's oesophagus or oesophageal cancer.	*"I never thought any further than taking the test, really. Well, I mean I don't smoke, I don't drink so I didn't expect anything other than a clear."*<br>(age 80+, positive result)
	<u>Sense of shock due to connotations of cancer more generally</u><br>Receiving a positive Cytosponge result was experienced to some degree as being like receiving a cancer diagnosis for some participants.	*"I think it was just a shock to hear that, you automatically… when I've read the leaflets and that and it's like Barrett's oesophagus is like looking for cancer, you just automatically always have that word in the back of your head, which I still have."*<br>(age 50-59, positive result)

	Sense of confusion or concern about test result meaning<br>Some participants receiving positive test results felt that the use of the word "positive" was difficult to understand, as they initially interpreted it in the lay sense of meaning "good".<br>Another cause of concern was about how to communicate the positive test result to family members. Some participants did not have an adequate understanding of what the test result meant to be able to explain it reassuringly to their family members.	*"I think saying positive is like saying you've got it. It would be like a possibility that needs further investigation or something like that."*<br>(age 50-59, positive result)<br><br>*"I suppose really it may have been better for possible where you get a positive is maybe sit there in front of the GP with your wife and then explain, because I had no idea what Barrett's was and you can look it up and it tells you all sorts of... and it's not the best way to look at anything, is it?"*<br>(age 50-59, positive result)
	No particularly strong reaction to positive result<br>Some participants did not react strongly to their result. In some cases, this was due to previous experiences that had given them relevant literacy or knowledge on cancer and cancer test results. In other cases, it was because the participant felt they had the necessary coping skills and attitudes, such as feeling there was no point in worrying, or that any problems can be managed or planned around.	*"… it didn't worry me, I had no problem. I want to know, end of, whatever you're going to throw at me, as long as I can plan it, that's how I live my life."*<br>(age 60-69, positive result)
Negative	Sense of relief<br>The negative result alleviated a sense of uncertainty or anxiety for some participants. This sometimes extended to a sense of relief on behalf of their families. In some cases, participants felt relieved to get confirmation that they were not on the same trajectory as family members who had previously suffered from issues related to reflux, or to get confirmation that their PPI medication had been effective.	Researcher: *Alright then, and did you have any emotional feelings about having that result at all, apart from relief?*<br>Participant: *No, just relief really. And the family as well.*<br>Interviewer: *Yeah.*<br>Participant: *Because I got to that stage now where my children think they should look after me, so it was a relief to them as well.*<br>(age 70-79, negative result)
	No particularly significant reaction, or a mildly positive reaction<br>This was sometimes due to participants simply having expected a negative result, while others simply had the attitude that there was no point in worrying.	*"I wasn't particularly bothered. There would be either something wrong or not"*<br>(age 70-79, negative result)
Inadequate (low confidence/ equivocal/ processing failure)	Understood the result<br>Some participants understood what this result meant and the reasons behind it and were willing to attend a second appointment for another procedure. They reported understanding that the reason for their result was that there were not enough cells collected.	*"The first time I didn't receive any notification. It was about two...just over two weeks, but that possibly was to do with the fact that they hadn't been able to take enough cells, and that notification was just to say that... apologising there wasn't enough cells, and would I mind coming back? And I said no, it's fine. And the second time I received a letter quite quickly, about seven to ten days afterwards, saying that the cells were all normal."*<br>(age 60-69, inadequate result)

	A sense of confusion about what the result meant<br>Some participants seemed unaware that this result was possible. They wondered why the test had not worked as expected and had not collected enough cells, and if theirs was the only case of this occurring. In some cases, this experience generated mistrust. This suggests that participants needed clearer information about how this result might come about and how common it is.	*"Well it did cross my mind that I wasn't being told the truth the first time. [...] I just wondered about it. [...] But I was assured that wasn't the case, I wasn't told anything that wasn't the truth. I was told there were too few cells collected."*<br>(age 70-79, inadequate result from first test, negative result from second test)

Supplementary Box 1. Types of discomfort during removal of the Cytosponge reported by patients being interviewed.

"Notable" pain, pain that was "horrible" or "worse than was expecting"

However, in these instances, some participants noted that the pain was to be expected (and interpreted the pain as a sign that the Cytosponge was effectively gathering cells). Alternatively, some participants felt the pain was so brief that it was not a problem. In these cases where pain was experienced, participants also said they would still be willing to have the Cytosponge procedure again, suggesting that an understanding of the mechanisms of the Cytosponge and the speed of its removal were factors that made the pain more tolerable. This was also confirmed by responses to the IAPS questionnaire: out of the 193 participants agreeing with the statement "Pulling up of the Cytosponge™ caused me great discomfort" (Table 2), 113 (59%) said that they would be willing to have another test if necessary.

Gagging

Some participants mentioned that this was unpleasant but also that, because the removal process was so fast that the gagging was not much of a problem, they would be willing to have the Cytosponge procedure again. Again, this was confirmed by responses to the IAPS questionnaire: out of the 889 participants agreeing with the statement "I had to gag when the Cytosponge™ was pulled up" (Table 2), 688 (79%) said they would be willing to have another test if necessary.

Roughness or scratching from the Cytosponge

In some cases, this caused the throat to immediately feel sore. One participant commented, however, that they understood the need for the Cytosponge to be rough, otherwise it would not effectively gather cells.

Weird or unexpected sensations

For example, the removal felt funny, weird, disconcerting, strange, or "that you can literally feel it being pulled like it could cut the back of your tongue. But I don't think it actually did because I had no pain afterwards so I was fine" [age 60-69, negative result].

Supplementary Table 3. Overall STAI-6 score at Cytosponge-TFF3 appointment (baseline) and 7-14 day follow-up for participants completing both questionnaires.

**Overall STAI-6 score**		**At follow-up**				
		20<40	40<60	60-80	Missing	**Total**
**At baseline**	20<40	858	82	2	48	**990**
	40<60	300	142	12	21	**475**
	60-80	7	13	2	1	**23**
	**Total**	**1165**	**237**	**16**	**70**	**1488**

Maroni R, *et al. BMJ Open* 2022; 12:e054258. doi: 10.1136/bmjopen-2021-054258

Supplementary Table 4. Perceived risk of oesophageal cancer compared to someone of the same age at Cytosponge-TFF3 appointment (baseline) and 7-14 day follow-up for participants completing both questionnaires.

**Relative risk of oesophageal cancer**		**At follow-up**					**p-value for McNemar's test (comparing "less than others" vs "more than others")**
		Less than others	Same as others	More than others	Missing	**Total**	
**At baseline**	Less than others	130	189	36	17	**372**	< 0.001
	Same as others	56	570	94	19	**739**	
	More than others	4	102	266	5	**377**	
	**Total**	**190**	**861**	**396**	**41**	**1488**	

Supplementary Table 5. Perceived percent risk of developing oesophageal cancer in a lifetime at Cytosponge-TFF3 appointment (baseline) and 7-14 day follow-up for participants completing both questionnaires.

**Percent absolute risk of oesophageal cancer**		**At follow-up**					**p-value for McNemar's test (comparing 0%, 5% vs 25%, 50%, 75%, 100%)**
		0%, 5%	10%	25%, 50%, 75%, 100%	Missing	**Total**	
**At baseline**	0%, 5%	396	108	181	27	**712**	< 0.001
	10%	70	123	100	12	**305**	
	25%, 50%, 75%, 100%	31	56	375	8	**470**	
	Missing	0	0	1	0	**1**	
	**Total**	**497**	**287**	**657**	**47**	**1488**	

SUPPLEMENTARY FIGURES

Supplementary Figure 1. (a) Trial flowchart for the patient-reported experience analysis of the BEST3 trial. (b) Venn diagram with the three subgroups of participation outlined in the analysis.

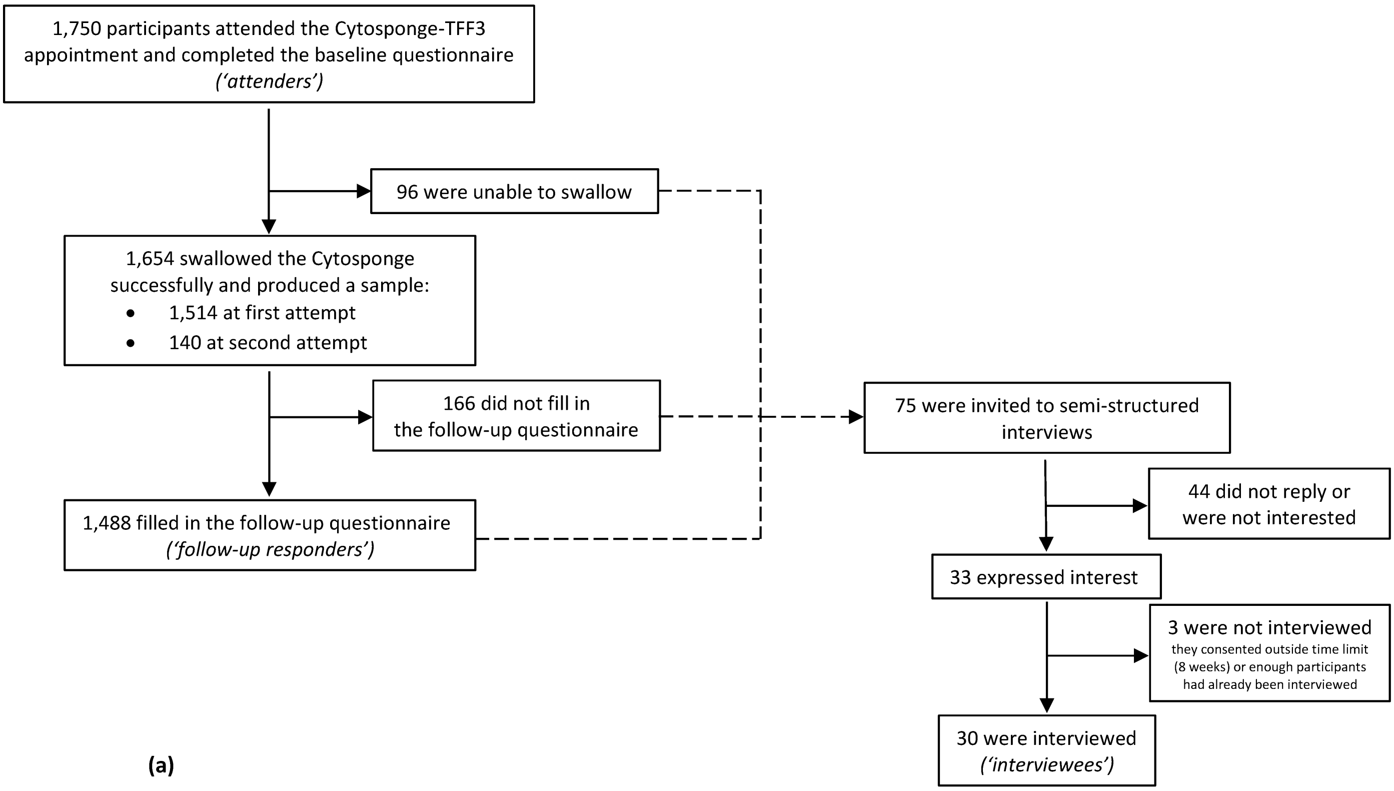

(a)

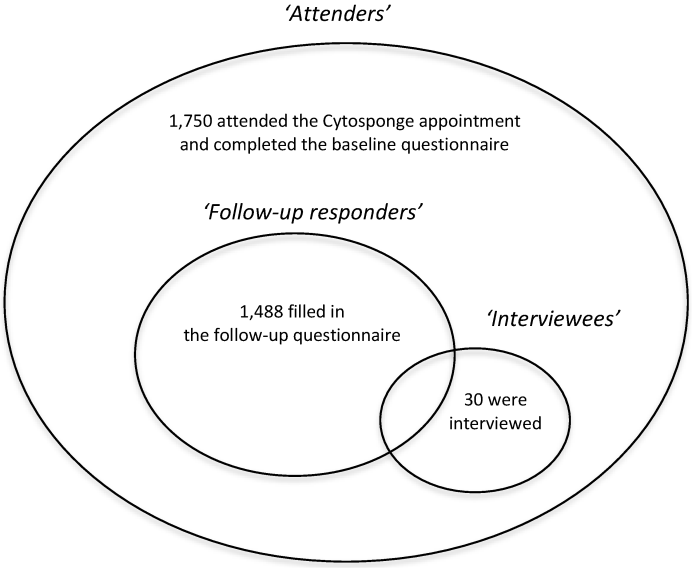

(b)

APPENDIX - QUESTIONNAIRES

A. BASELINE CLINICAL FORM

GENERAL INFORMATION

1. Height ……….. cm
2. Weight ……….. kg
3. What was your weight aged 20?
   - Underweight
   - Normal range
   - Overweight
   - Obese
   - Don't remember
4. Have you ever been obese?
   - Yes
   - No
5. Waist circumference ……….. cm
6. Hip circumference ……….. cm

MEDICATION

7. Medication type for reflux symptoms
   - H2 receptor antagonists
   - Proton pump inhibitor
   - Over the counter anti-acids
   - Other

7a. Name: ………..

7b. Dose: ………..

7c. Units:
- mg
- g
- ml
- tablet

7d. Frequency:
- OD
- BD
- TDS
- QDS
- PRN

7e. Month/year started:
- 0-1 year
- 1-2 years
- 3-4 years
- 4-5 years
- 5-6 years
- 6+ years

8. Please confirm whether patient has any comorbidities:
   - Yes
   - No

[COMORBIDITY TYPES COLLECTED IN SEPARATE SUBFORM]

SYMPTOMS

9. Gastro-oesophageal Reflux Disease Impact Scale (GIS) – part A

Please complete the following questions by marking one response per question. Consider your symptoms prior to you taking any acid suppressant medication. There are no right or wrong answers. Be sure to answer every question.

**Prior to you taking any acid suppressant medication please confirm the following…**	**DAILY**	**OFTEN**	**SOMETIMES**	**NEVER**
1. How often did you have the following symptoms:				
a. Pain in your chest or behind the breastbone?				
b. Burning sensation in your chest or behind the breastbone?				
c. Regurgitation or acid taste in your mouth?				
d. Pain or burning in your upper stomach?				
e. Sore throat or hoarseness that was related to your heartburn or acid reflux?				
2. How often did you have difficulty getting a good night's sleep because of your symptoms?				
3. How often did your symptoms prevent you from eating or drinking any of the foods you like?				
4. How frequently did your symptoms keep you from being fully productive in your job or daily activities?				

5. How often did you buy over-the-counter medication (such as Rennies, Tums, Gaviscon)?				

10. Gastro-oesophageal Reflux Disease Impact Scale (GIS) – part B

Please complete the following questions by marking one response per question. Consider your symptoms over the past week. There are no right or wrong answers. Be sure to answer every question.

In the past week…	DAILY	OFTEN	SOMETIMES	NEVER
1. How often have you had the following symptoms:				
f. Pain in your chest or behind the breastbone?				
g. Burning sensation in your chest or behind the breastbone?				
h. Regurgitation or acid taste in your mouth?				
i. Pain or burning in your upper stomach?				
j. Sore throat or hoarseness that is related to your heartburn or acid reflux?				
2. How often have you had difficulty getting a good night's sleep because of your symptoms?				
3. How often have your symptoms prevented you from eating or drinking any of the foods you like?				
4. How frequently have your symptoms kept your from being fully productive in your job or daily activities?				
5. How often do you take additional medication other than what the physician told you to take (such as Rennies, Tums, Gaviscon)?				

FURTHER INFORMATION

11. How long ago did your heartburn first begin?

- Never
- Last 6 months
- 7 months to 1 year
- 1 to 2 years
- 2 to 5 years
- 5 to 10 years
- 10 to 20 years
- More than 20 years

12. How long ago did you first notice the acid/sour taste in your mouth?

- Never
- Last 6 months
- 7 months to 1 year
- 1 to 2 years
- 2 to 5 years
- 5 to 10 years
- 10 to 20 years
- More than 20 years

13. Have you been prescribed treatment for H.pylori?

- Yes
- No
- Don't know

14. Did the treatment for H.pylori make your symptoms:

- Worse
- No change
- Better

15. Are you taking medicine for your stomach symptoms?

- Yes
- No

B. BASELINE CLINICAL FORM

Lifestyle/family history

EDUCATION

1. What is the highest level of education that you have achieved?
   - School up to 15-16 years
   - College or vocational study
   - University graduate
   - Professional training beyond college or postgraduate degree
   - Other
   - Prefer not to say
2. If other, please specify ....

SMOKING

3. How many hours a day are you exposed to other people's smoke?
   - 0 hours
   - 1-6 hours
   - 6-12 hours
   - 12-18 hours
   - 18-24 hours
4. Have you ever smoked cigarettes, tobacco, pipe or cigars?
   - Yes
   - No
5. Age when you started smoking ....
6. Have you stopped smoking?
   - Yes
   - No
7. If you are no longer smoking, at what age did you stop? ....

How many/much did you or do you smoke per day of:

8. Cigarettes ....
9. Cigars ....
10. Tobacco (cigarettes/pipe) .... oz or grams

ALCOHOL HISTORY

11. Which one of the following best describes your present alcohol intake?
    - None
    - Daily or most days
    - Weekends only
    - Occasional (once / twice per month)
12. Which of the following is your preferred beverage(s)?
    - Red wine
    - White wine
    - Spirits
    - Beer
    - Alcopops
    - Other
    - Prefer not to say
13. If other, please specify ....
14. At present, how many units do you drink a week?
    - 1-5 units
    - 6-10 units
    - 11-15 units
    - 16-20 units
    - 21-25 units
    - 26-30 units
    - 30+ units
    - Not sure
    - Prefer not to say
15. Did you ever drink heavily in the past? (Heavy drinking is defined as >14 units per week for women and >21 units per week for men)
    - Yes
    - No
    - Not sure
    - Prefer not to say
16. How many units a week did you drink when you were 20?
    - 0 units
    - 1-5 units
    - 6-10 units
    - 11-15 units
    - 16-20 units
    - 21-25 units
    - 26-30 units
    - 30+ units
    - Not sure
    - Prefer not to say

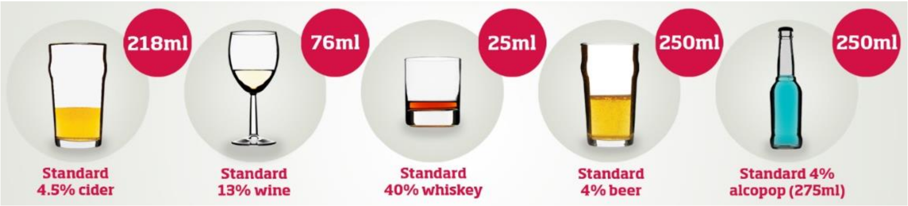

Courtesy of Public Health England

FAMILY HISTORY

17. Do any of your family have any of the following: heartburn, Barretts's oesophagus, cancer of the gullet/oesophagus, any other cancer and type.
    - Yes
    - No

18. (Please answer all questions for the relatives this is applicable for)

Relative	Heartburn	Barrett's oesophagus	Cancer of the gullet or oesophagus	Any other cancer and type

Perceived risk of developing oesophageal cancer

These questions are about how susceptible you feel to oesophageal cancer.

Compared to a person of the same age as you, what are your chances of developing oesophageal cancer? *(Please tick one)*	Much lower ☐<br>Lower ☐<br>Neither higher nor lower ☐<br>Higher ☐<br>Much higher ☐
In your lifetime, what do you consider your risk of developing oesophageal cancer is? *(Please tick one)*	0% ☐<br>5% ☐<br>10% ☐<br>25% ☐<br>50% ☐<br>75% ☐<br>100% ☐

Short-form of the state scale of the Spielberger State-Trait Anxiety Inventory (STAI-6)

A number of statements which people have used to describe themselves are given below. Read each sentence and then circle the most appropriate number to the right of the statement to indicate how you feel RIGHT NOW, AT THIS MOMENT. There are no right or wrong answers. Do not spend too much time on any one statement but give the answer which seems to describe your present feelings best.

(Please tick one box for each statement)

	Not at all	Somewhat	Moderately	Very much
**I feel calm**	1	2	3	4
**I am tense**	1	2	3	4
**I feel upset**	1	2	3	4
**I am relaxed**	1	2	3	4
**I feel content**	1	2	3	4
**I am worried**	1	2	3	4

Maroni R, *et al. BMJ Open* 2022; 12:e054258. doi: 10.1136/bmjopen-2021-054258

C. 7-14 DAY FOLLOW-UP QUESTIONNAIRE

Inventory to Assess Patient Satisfaction (IAPS)

You recently received the Cytosponge™ test at your practice as part of the BEST3 Trial. On a scale of 1-5, please indicate whether you agree or disagree with the following statements:

(Please circle one response per statement)

	Strongly agree	Agree	Not sure	Disagree	Strongly disagree
**Convenience and accessibility**					
I felt that i had to wait too long.	1	2	3	4	5
The test is in a place that is easy for me to get to.	1	2	3	4	5
I found it hard to find a convenient time to come to the test.	1	2	3	4	5
**Staff interpersonal skills**					
I felt free to ask the staff questions i wanted to ask.	1	2	3	4	5
The staff seemed to hurry me through too quickly.	1	2	3	4	5
The staff used words that were hard to understand.	1	2	3	4	5
**Perceived technical competence**					
The nurse or member of staff was too rough when performing the Cytosponge test.	1	2	3	4	5
I feel confident that the Cytosponge test was performed properly.	1	2	3	4	5
**Swallowing of the capsule**					
I had to gag when I swallowed the Cytosponge capsule.	1	2	3	4	5
Swallowing the Cytosponge capsule was more comfortable than i expected.	1	2	3	4	5
Swallowing the Cytosponge capsule caused me great discomfort.	1	2	3	4	5
**Waiting with capsule in stomach**					
I had to gag while I waited with the Cytosponge capsule in my stomach.	1	2	3	4	5
Waiting with the Cytosponge capsule in my stomach was more comfortable than i expected.	1	2	3	4	5
Waiting with the Cytosponge capsule in my stomach caused me great discomfort.	1	2	3	4	5
**Pulling up of the Cytosponge**					
I had to gag when the Cytosponge was pulled up.	1	2	3	4	5
Pulling up of the Cytosponge was more comfortable than i expected.	1	2	3	4	5
Pulling up of the Cytosponge caused me great discomfort.	1	2	3	4	5
**Expectations and beliefs**					
I was very anxious about having the Cytosponge test.	1	2	3	4	5
Undergoing the Cytosponge test will benefit my health.	1	2	3	4	5
**General satisfaction**					
I was very satisfied with the care I received.	1	2	3	4	5
I would recommend the Cytosponge test to my friends.	1	2	3	4	5
I would be willing to have another if necessary.*	1	2	3	4	5

*As part of the Trial, you may still be invited for a repeat Cytosponge test.

Cytosponge test experience questionnaire

Please mark on the line your experience with the Cytosponge test:

0	5	10
Completely unacceptable	*Neither unacceptable nor acceptable*	*Completely acceptable*

Comments:

Perceived risk of developing oesophageal cancer

These questions are about how susceptible you feel to oesophageal cancer.

Compared to a person of the same age as you, what are your chances of developing oesophageal cancer? *(please tick one)*	Much lower ☐<br>Lower ☐<br>Neither higher nor lower ☐<br>Higher ☐<br>Much higher ☐
In your lifetime, what do you consider your risk of developing oesophageal cancer is? *(please tick one)*	0% ☐<br>5% ☐<br>10% ☐<br>25% ☐<br>50% ☐<br>75% ☐<br>100% ☐

Short-form of the state scale of the Spielberger State-Trait Anxiety Inventory (STAI-6)

A number of statements which people have used to describe themselves are given below. Read each sentence and then circle the most appropriate number to the right of the statement to indicate how you feel RIGHT NOW, AT THIS MOMENT. There are no right or wrong answers. Do not spend too much time on any one statement but give the answer which seems to describe your present feelings best.

(Please tick one box for each statement)

	Not at all	Somewhat	Moderately	Very much
I feel calm	1	2	3	4
I am tense	1	2	3	4
I feel upset	1	2	3	4
I am relaxed	1	2	3	4
I feel content	1	2	3	4
I am worried	1	2	3	4

Maroni R, *et al. BMJ Open* 2022; 12:e054258. doi: 10.1136/bmjopen-2021-054258
