# Supplementary material for: Patient-reported experiences and views on the Cytosponge test: a mixed-methods analysis from the BEST3 trial
Source: BMJ Open. 2022 Apr 7;12(4):e054258. doi: 10.1136/bmjopen-2021-054258 (PMC8990713; doi:10.1136/bmjopen-2021-054258)
Supplement: Author's manuscript [file bmjopen-2021-054258.draft_revisions.pdf]

BMJ Open

BMJ Open is committed to open peer review. As part of this commitment we make the peer review history of every article we publish publicly available.

When an article is published we post the peer reviewers' comments and the authors' responses online. We also post the versions of the paper that were used during peer review. These are the versions that the peer review comments apply to.

The versions of the paper that follow are the versions that were submitted during the peer review process. They are not the versions of record or the final published versions. They should not be cited or distributed as the published version of this manuscript.

BMJ Open is an open access journal and the full, final, typeset and author-corrected version of record of the manuscript is available on our site with no access controls, subscription charges or pay-per-view fees (http://bmjopen.bmj.com).

If you have any questions on BMJ Open's open peer review process please email info.bmjopen@bmj.com

BMJ Open

Patient-reported experiences and views on the Cytosponge test: a mixed-methods analysis from the BEST3 trial

Journal:	*BMJ Open*
Manuscript ID	bmjopen-2021-054258
Article Type:	Original research
Date Submitted by the Author:	09-Jun-2021
Complete List of Authors:	Maroni, Roberta; King's College London, Cancer Prevention Trials Unit (CPTU), Cancer Prevention Group, School of Cancer & Pharmaceutical Sciences<br>Barnes, Jessica; King's College London, Cancer Prevention Group, School of Cancer and Pharmaceutical Sciences<br>Offman, Judith; King's College London, Cancer Prevention Group, School of Cancer and Pharmaceutical Sciences<br>Scheibl, Fiona; University of East Anglia, Norwich Medical School<br>Smith, Samuel; University of Leeds, Leeds Institute of Health Sciences<br>Debiram-Beecham, Irene; University of Cambridge, MRC Cancer Unit, Hutchison-MRC Research Centre<br>Waller, Jo; King's College London, Cancer Prevention Group, School of Cancer and Pharmaceutical Sciences<br>Sasieni, Peter; King's College London, Cancer Prevention Group, School of Cancer and Pharmaceutical Sciences<br>Fitzgerald, RC; University of Cambridge, MRC Cancer Unit, Hutchison-MRC Research Centre; Cambridge University Hospitals NHS Foundation Trust<br>Rubin, Greg; Newcastle University, Population Health Sciences Institute<br>Walter, Fiona; University of Cambridge, The Primary Care Unit, Department of Public Health & Primary Care; Queen Mary University of London, Institute of Population Health Sciences
Keywords:	PRIMARY CARE, Gastrointestinal tumours < GASTROENTEROLOGY, Gastrointestinal tumours < ONCOLOGY, PREVENTIVE MEDICINE, PUBLIC HEALTH

SCHOLARONE™
Manuscripts

I, the Submitting Author has the right to grant and does grant on behalf of all authors of the Work (as defined in the below author licence), an exclusive licence and/or a non-exclusive licence for contributions from authors who are: i) UK Crown employees; ii) where BMJ has agreed a CC-BY licence shall apply, and/or iii) in accordance with the terms applicable for US Federal Government officers or employees acting as part of their official duties; on a worldwide, perpetual, irrevocable, royalty-free basis to BMJ Publishing Group Ltd ("BMJ") its licensees and where the relevant Journal is co-owned by BMJ to the co-owners of the Journal, to publish the Work in this journal and any other BMJ products and to exploit all rights, as set out in our licence.

The Submitting Author accepts and understands that any supply made under these terms is made by BMJ to the Submitting Author unless you are acting as an employee on behalf of your employer or a postgraduate student of an affiliated institution which is paying any applicable article publishing charge ("APC") for Open Access articles. Where the Submitting Author wishes to make the Work available on an Open Access basis (and intends to pay the relevant APC), the terms of reuse of such Open Access shall be governed by a Creative Commons licence – details of these licences and which Creative Commons licence will apply to this Work are set out in our licence referred to above.

Other than as permitted in any relevant BMJ Author's Self Archiving Policies, I confirm this Work has not been accepted for publication elsewhere, is not being considered for publication elsewhere and does not duplicate material already published. I confirm all authors consent to publication of this Work and authorise the granting of this licence.

For peer review only

Patient-reported experiences and views on the Cytosponge test: a mixed-methods analysis from the BEST3 trial

Roberta Maroni[1*], Jessica Barnes[2*], Judith Offman[2*], Fiona Scheibl[3], Samuel G Smith[4], Irene Debiram-Beecham[5], Jo Waller[2], Peter D Sasieni[2], Rebecca C Fitzgerald[5,6], Greg Rubin[7], BEST3 Consortium[6], Fiona M Walter[9,10]

[1] Cancer Research UK and King's College London Cancer Prevention Trials Unit, Cancer Prevention Group, School of Cancer and Pharmaceutical Sciences, King's College London, London, UK

[2] Cancer Prevention Group, School of Cancer and Pharmaceutical Sciences, King's College London, London, UK

[3] Norwich Medical School, University of East Anglia, Norwich, UK

[4] Leeds Institute of Health Sciences, University of Leeds, Leeds, UK

[5] MRC Cancer Unit, Hutchison-MRC Research Centre, University of Cambridge, Cambridge, UK

[6] Cambridge University Hospitals NHS Foundation Trust, Cambridge, UK

[7] Population Health Sciences Institute, Newcastle University, 5th Floor, Ridley 1, Newcastle upon Tyne, UK

[8] BEST3 Trial team NIHR, Clinical Research Networks, UK

[9] The Primary Care Unit, Department of Public Health & Primary Care, University of Cambridge, UK

[10] Institute of Population Health Sciences, Queen Mary University of London, UK

*Roberta Maroni, Jessica Barnes and Judith Offman equally contributed to this paper.

Corresponding author:

Jessica Barnes, King's College London, School of Cancer & Pharmaceutical Sciences, Innovation Hub, Guys Cancer Centre, Guys Hospital, Great Maze Pond, London SE1 9RT, United Kingdom, jessica.barnes@kcl.ac.uk

WORD COUNT (excluding title page, abstract, references, figures and tables):

4,371

For peer review only

ABSTRACT

Objectives: The BEST3 trial demonstrated the efficacy and safety of the Cytosponge-TFF3, a cell collection device coupled with the biomarker trefoil factor 3, as a tool for detecting Barrett's oesophagus, a precursor of oesophageal adenocarcinoma (OAC), in primary care. In this nested study, our aim was to understand patient experiences.

Design: Mixed methods using questionnaires (including Inventory to Assess Patient Satisfaction, STAI-6 and two-item perceived risk) and interviews.

Outcome measures: Participant satisfaction, anxiety and perceived risk of developing OAC.

Setting: General practices in England.

Participants: Patients with acid reflux enrolled in the intervention arm of the BEST3 trial and attending the Cytosponge appointment (N = 1750).

Results: 1488 patients successfully swallowing the Cytosponge completed the follow-up questionnaires, while 30 were interviewed, including some with an unsuccessful swallow.

Overall, participants were satisfied with the Cytosponge test. Several items showed positive ratings, in particular convenience and accessibility, staff's interpersonal skills and perceived technical competence. The most discomfort was reported during the Cytosponge removal, with more than 60% of participants experiencing gagging. Nevertheless, about 80% were willing to have the procedure again or to recommend it to friends; this was true even for participants experiencing discomfort, as confirmed in the interviews.

Median anxiety scores were below the pre-defined level of clinically significant anxiety and slightly decreased between baseline and follow-up ($p < 0.001$). Interviews revealed concerns around the ability to swallow, participating in a clinical trial, and waiting for test results.

The perceived risk of OAC increased following the Cytosponge appointment ($p < 0.001$). Moreover, interviews suggested that some participants had trouble conceptualising risk and did not understand the relationships between test results, gastro-oesophageal reflux, and risk of Barrett's oesophagus and OAC.

Conclusions: When delivered during a trial in primary care, the Cytosponge is well accepted and causes little anxiety.

Trial registration: ISRCTN68382401

Keywords: Barrett's oesophagus, oesophageal cancer, Cytosponge, anxiety, perceived risk, patient satisfaction, acceptability, mixed methods, questionnaires, interviews, primary care.

ARTICLE SUMMARY

Strengths and limitations of this study

- Our study is the first to explore patients' experiences of, and satisfaction with, the Cytosponge test in the primary care setting, gaining in-depth understanding by using questionnaire and interview data in a mixed-methods approach.

- Thirty participants, purposively sampled to reflect a range of characteristics (gender, age group, geographic region, Cytosponge result), underwent semi-structured interviews, whose analyses were underpinned by a robust approach, including a conceptual framework.

- A small proportion (10%) of patients undergoing the Cytosponge test did not complete a follow-up questionnaire.

For peer review only - http://bmjopen.bmj.com/site/about/guidelines.xhtml

- The Inventory to Assess Patient Satisfaction in the follow-up questionnaire was adapted from flexible sigmoidoscopy and validated on a small number of individuals.

INTRODUCTION

In the UK, oesophageal adenocarcinoma (OAC) is the seventh most common cause of cancer death. It has a bleak prognosis, with a 5-year net survival of just 17%.[1] Barrett's oesophagus (BE) is a pre-cancerous lesion that develops into OAC in up to 1% of the patients affected with it per year [2], but it is diagnosed in only 20% of patients prior to developing OAC.[3] An important risk factor for BE is gastro-oesophageal reflux disease (GORD), which burdens about 20% of the adult population [4] and is usually managed effectively with acid-suppressant medications and endoscopy referrals, which are suggested by NICE only if the symptoms are not controlled.[5] However, endoscopies are invasive, expensive [6] and entail some risks.[7] Given that pressures on endoscopy capacity in secondary care in the UK have been exacerbated by the recent COVID-19 pandemic,[8] novel technologies to help detect BE are now more important than ever.

The Cytosponge is a cell collection device, which, coupled with the biomarker trefoil factor 3 (TFF3), can be used to identify BE. The device consists of a sponge tied to a string and compressed into a gelatine capsule, which is swallowed by the patient and retrieved by pulling on the string once the capsule has dissolved. The cell sample is then processed in a laboratory for immunohistochemical staining with TFF3. The Cytosponge-TFF3 test has been evaluated among more than 2000 patients in two clinical settings,[9, 10] proving its safety, cost-effectiveness, and accuracy as a potential test for BE.[11-14] The large (N > 13,000), pragmatic, randomised, controlled BEST3 trial was recently conducted in primary care in England [15] and demonstrated that offer of the Cytosponge test to individuals on medication for recurrent reflux symptoms identified ten times more cases of BE than usual care. In this trial, fewer than 10% of participants successfully swallowing the Cytosponge reported any side effects, and those were mainly mild (e.g. sore throat).

Successful implementation of a new diagnostic device requires not only evidence on diagnostic accuracy, safety, effectiveness and cost-effectiveness, but also an understanding of patient experience and satisfaction, including the identification of possible barriers to uptake. During the BEST3 trial, where patients were invited to receive the Cytosponge by postal letter, uptake was 24% (1654/6834) and median overall acceptability on an 11-point visual analogue scale from 'completely unacceptable' to 'completely acceptable' was 9 (interquartile range (IQR) 8-10).[15] We undertook a nested mixed-methods study within BEST3, aiming to evaluate patients' experiences of the Cytosponge test in primary care, any anxiety caused by the test, and their perceived risk of OAC.

METHODS

Study design

The design of the BEST3 trial is described in more detail in the Supplementary Materials and elsewhere.[15, 16] It enrolled participants aged 50 or over with GORD symptoms, identified via their general practice prescribing records. For this nested study, only participants in the intervention arm of the trial attending a Cytosponge appointment were included (N = 1750). Participants with an 'inadequate' test result (i.e. low-confidence negative TFF3, equivocal, or processing/technical failure) were invited to a repeat appointment when possible. All patients with a positive TFF3 result were referred for an endoscopy to establish a diagnosis.

Patient and public involvement

Patient and public involvement representatives were involved in all stages of the BEST3 trial, including two as members of the BEST3 trial steering committee;[16] they reviewed the protocol and the interview topics, and contributed to the early analysis of the patient interviews. The adapted Inventory to Assess Patient Satisfaction

For peer review only - http://bmjopen.bmj.com/site/about/guidelines.xhtml

(IAPS) was piloted on eight individuals who had previously had the Cytosponge procedure to check for comprehension.

Data collection

a. Quantitative data

At the Cytosponge appointment, in each participant's general practice, a nurse collected demographic, anthropometric and clinical data, including the GORD Impact Scale.[17] This is a nine-item assessment of GORD symptoms experienced in the week before the appointment, which was duplicated to also address any symptoms from before patients started taking acid-suppressant medications.

Immediately before having the test, participants were asked to complete the baseline questionnaire, with questions on:

- Education level, smoking/alcohol history, family history of heartburn/BE/cancer;
- A shorter six-item form of the Spielberger State-Trait Anxiety Inventory (STAI-6);[18]
- Perceived risk of OAC, using two items widely applied for other cancer risk assessments: perceived risk compared with a person of the same age (comparative risk) and percent absolute risk of developing OAC in their lifetime.[19]

A week to 14 days later, participants who successfully swallowed the Cytosponge (1654/1750, 95%) were invited to fill in a follow-up questionnaire. A reminder to complete the questionnaire was sent after two weeks, and some received a further reminder. The follow-up questionnaire consisted of:

- The IAPS, with 22 items addressing both positive and negative aspects of the experience, adapted from a study on flexible screening sigmoidoscopy [20];
- STAI-6;
- Perceived risk of OAC (two items).

b. Qualitative data

Participants were purposively sampled to reflect a range of characteristics: gender, age group (50-59, 60-69, 70-79, 80+), geographic region in England (East, North-East, West) and Cytosponge result at first appointment (positive, negative, low confidence/equivocal, unsuccessful swallow), see Table 1 and Supplementary Table 1. Semi-structured interviews were conducted face-to-face at home or in clinics in the only presence of a female qualitative researcher (FS), with the aim of interviewing 30 participants within six weeks of their Cytosponge test (to reduce issues with recall). Interviews lasted 23 minutes on average (range: 13-50 minutes), were audio-recorded and transcribed verbatim for analysis. More details on qualitative data collection are in the Supplementary Materials.

Analysis

a. Quantitative analysis

Questionnaire scoring is described in the Supplementary Materials. Patient characteristics and GORD Impact Scale responses were analysed according to three subgroups of participation: attended the Cytosponge test appointment and completed the baseline questionnaire *('attenders'); 'attenders'* who completed the follow-up questionnaire *('follow-up responders')*; *'attenders'* who undertook an interview *('interviewees')*, see Supplementary Figure 1.

STAI-6 and perceived risk of OAC are presented only for the subgroup of participants completing at least one of those items in both baseline and follow-up questionnaires. STAI-6 scores between the two time points were compared using Wilcoxon matched-pairs signed-rank test, while differences in risk perceptions were analysed by McNemar's test, which included only patients with scores different than the neutral (e.g. 'Neither higher or

For peer review only - http://bmjopen.bmj.com/site/about/guidelines.xhtml

lower') or middle-ranking category (from a list of ordered options). A STAI-6 score over 40 was predefined as a threshold for clinically significant anxiety.[9, 21]

Statistical significance was based on a two-tailed test with size of 5%. Analyses were performed using Stata version 15.[22]

b. Qualitative analysis

We undertook a thematic analysis, having organised and managed data according to the Framework approach.[23] For more details, see Supplementary Materials. Briefly, this involved identifying an initial, broad set of labels inductively and deductively that would be used to categorise and sort the data to enable the subsequent thematic analysis. Inductively-created labels were based on emergent concepts identified in the data. Deductively-created labels were based on the IAPS [20], which allowed us to more directly relate participant experience across qualitative and quantitative datasets. Use of the Theoretical Framework of Acceptability [24] constructs allowed us to examine additional dimensions of patient experience associated with acceptability that were not captured by the IAPS. We then conducted the thematic analysis, aiming to achieve both description and explanation with the dataset. Data within each column of the Framework matrix was explored and further organised into more abstract themes, using drawings.net open-source software to allow visual representation and coding of the data, therefore facilitating the identification patterns and linkages between different types of participant experience and/or demographic characteristics. Participants did not provide feedback on the findings.

RESULTS

Demographics and baseline characteristics

A trial flowchart from the intervention arm of the BEST3 trial is shown in Supplementary Figure 1. There were 1750 participants who completed the baseline questionnaire at Cytosponge appointment *('attenders')*, with a minimum completion rate of 80% (12/15), considering only questions applicable to all participants. The follow-up questionnaire was completed by 1488 participants (90% of 1654 successful swallows) *('follow-up responders')*, with a minimum completion rate of 23% (7/31) at a median of 10 days (IQR 7-14 days) after undergoing the Cytosponge test. 159 participants (11% of 1488) completed the follow-up questionnaire after being mailed the letter with their Cytosponge test results, 5 (0.3%) after attending a repeat Cytosponge test and one after receiving their repeat Cytosponge test result.

Out of the 1750 *'attenders'*, 75 (4%) were invited for an interview; 30 interviews were completed *('interviewees')* at a median of 59 days (IQR 48-78 days) after the Cytosponge test. At the time of interview, all participants who successfully swallowed the Cytosponge had received their first test result, and one was still waiting for the result from their repeat test, while another had declined to have a repeat test. Among participants who had received a positive test result, some were awaiting confirmatory endoscopy, while others had already had theirs.

Table 1 shows patient and clinical characteristics for the three subgroups of participants. Those completing the follow-up questionnaire differed from the non-completers (N = 262) by age group, waist-hip ratio categories and comorbidity status.

1. Patient-reported satisfaction and experiences of the Cytosponge test

Participants were generally satisfied with their experiences of the processes undertaken before, during and immediately after the Cytosponge test (Figure 1). Several items of the IAPS were rated positively by most participants (Table 2). The Cronbach's α, measuring the IAPS reliability, was 0.83 overall, and it ranged between 0.81 and 0.83 (improving the overall coefficient in three instances) when excluding each of the 22 items at a time.

For peer review only - http://bmjopen.bmj.com/site/about/guidelines.xhtml

Experiences linked to the understanding of the test results, discussed during the patient interviews, are presented in Supplementary Table 2.

1.1 Convenience and accessibility

The majority of participants (92-94%) rated the study sites' convenience and accessibility positively. In the interviews, some commented that it was practical to go to their own General Practice and that this was preferable to going to a hospital appointment. Participants also appreciated the scheduling, as they were able to select from a range of appointment dates and times that suited them.

1.2 Staff interpersonal skills

Staff interpersonal skills were rated positively by 96%-98% of participants, and uniformly described across the interviews in very good terms. Participants felt that they had adequate opportunities to ask questions, which the nurses were able to answer well providing important reassurance. The interpersonal manner of staff was consistently described in highly positive ways by participants – staff were "calm", "in control", "friendly", "helpful", "supportive", "professional", and created an experience that was "straightforward and bordering on enjoyable". When participants failed to swallow the Cytosponge, staff were empathetic and reassuring.

1.3 Perceived technical competence of staff

The majority of participants (93%-96%) agreed that the staff was competent. In the interviews, participants focused on the speed and efficiency of the Cytosponge removal – for example, that the staff members had good technique, and they went quickly enough to get removal done efficiently but slowly enough to gather cells. Some issues related to technical skills were noted: one participant described how their procedure was performed by a practice nurse, and the Cytosponge seemed to get stuck part way through removal and caused pain. In this case, the practice nurse needed to consult a research nurse who advised how to resolve the issue.

1.4 Swallowing the Cytosponge capsule

The majority of participants (69%-87%) rated swallowing the Cytosponge positively. Among the lowest rated measure of satisfaction was "*I had to gag when I swallowed the Cytosponge capsule*" (N = 373, 25% agreed).

When interview participants described the procedure as straightforward, they recounted the swallowing aspect as routine and nothing unexpected.

> *"I think that's just normal as taking a tablet, the only difference is it's got string on it."*
> *[age 60-69, negative result]*

When participants described the swallowing as involving minimal difficulty, they commented on characteristics such as the string being uncomfortable, or that it was difficult to drink enough water to get the string and capsule down. However, these difficulties were mainly perceived as nothing to worry about.

Interviewees who reported significant difficulty swallowing, such as gagging, retching or heaving, underlined issues with being unable to place the capsule and string far enough at the back of their throat without causing themselves to gag; in some cases, this could be rectified by the nurse placing the string and capsule instead of the participant. Participants who failed to swallow reported struggling with getting the string down as it unwound, and gagging too much to be able to drink water to wash the string and capsule down the oesophagus. One participant reported that they had not realised that they would be required to swallow the string in a bundle, and if they had known this, they may have declined to participate. During the interviews, responses were varied amongst the four participants who failed to swallow: some would not do the procedure again but would still recommend it to friends, while others would still try the procedure again in the future.

> *"Well swallowing the capsule was all right. The string attached to it was a bit difficult, it felt a bit like a cat trying to swallow a mouse, you know, can't get the tail in the mouth. [...] It went down all right… it was just an odd feeling with the string coming up."*
> *[age 60-69, negative result]*

For peer review only - http://bmjopen.bmj.com/site/about/guidelines.xhtml

1.5 Waiting with Cytosponge in stomach

Overall, 85-92% participants rated the experience of waiting for the capsule to dissolve in their stomach positively. During the interviews, some reported not being able to feel anything untoward at all, nor did they experience any distress. Others reported minor issues, such as being aware of the string, tickling or gagging when trying to talk, but these experiences were not considered concerning.

> *"The only strange sensation was... after I'd swallowed the pill, it was having a tiny piece of cotton or whatever it was hanging out, but the way [the nurses] talked, it took my mind off it anyway."*
> *[age 60-69, negative result]*

Some participants discussed more distressing experiences: one experienced "pains in my stomach", significant enough for them to ask the nurse to remove the Cytosponge prematurely. This procedure resulted in a low confidence/equivocal result, and the participant attended a repeat appointment, where they were able to successfully swallow the Cytosponge and it was "less uncomfortable" while the capsule was dissolving. The participant suggested that this may be because they drank more water the second time, causing the Cytosponge to successfully reach the stomach.

1.6 Retrieving the Cytosponge

Amongst the lowest rated measures of satisfaction were: "*I had to gag when the Cytosponge was pulled up*" (N = 889, 60% agreed) and "*Pulling up of the Cytosponge was more comfortable than I expected*" (N = 354, 24% disagreed).

During the interviews, participants gave more detailed descriptions of this part of the Cytosponge test, with some reporting a number of types of discomfort during removal (Figure 1b). Not all of them were particularly serious or concerning. Despite these experiences of discomfort, participants often expressed a willingness to have the Cytosponge test again and to recommend it to others, as confirmed by responses to the IAPS questionnaire (61% and 65% of participants with low average satisfaction scores for items about pulling the Cytosponge, respectively). One interviewee was unwilling to have the procedure again due to the perceived possibility of the string breaking and an endoscopy being necessary to retrieve it.

1.7 Expectations, beliefs and general satisfaction

A fifth of the participants (20%) agreed with the item *"I was very anxious about having the Cytosponge test"*, while 97% reported being very satisfied with the care received.

During the interviews, participants discussed a range of after-effects (including none). Some explained that they felt fine after their appointment, and sometimes forgot completely about it until they received their results letter. Some participants described experiencing a sore, scratchy or tickly throat that resolved relatively quickly. Some reported experiencing unexpected reflux following their test.

2. Patient-reported anxiety before and after the Cytosponge test

Participants who completed both pre- and post-test measures (N = 1418) had a median STAI-6 score of 33 (IQR 23-40; possible range: 20-80) at baseline and 27 (IQR 20-37) at follow-up. As a comparison, the median score for participants not filling in the follow-up questionnaire was 33 at baseline (IQR 23-43). A score of over 40 was predefined as meeting a clinical threshold of anxiety: 334 (24%) and 166 (12%) reported such scores at baseline and follow-up, respectively. There was a statistically significant difference in scores between baseline and follow-up ($p < 0.001$), with a median change between follow-up and baseline of -3 (IQR -10-0). For a breakdown of scores by questionnaire, see Supplementary Table 3.

Interviewees offered reflections on how they were feeling the day of the appointment or the night before. Some were worried about being able to complete the test (e.g. participants who had problems swallowing) or the test itself. A few were concerned about 'the unknown' and it being 'experimental'. Reflecting on the period after the Cytosponge test, some described how receiving their result alleviated the sense of anxiety and

For peer review only - http://bmjopen.bmj.com/site/about/guidelines.xhtml

uncertainty that they had been experiencing. Other participants, however, reported not being particularly bothered while waiting for their results.

3. Perceived risk of oesophageal cancer

Amongst participants filling in both questionnaires, just over half (N = 739, 50% at baseline; N = 861, 58% at follow-up) considered their risk to be equivalent to that of someone of the same age (Figure 2a). Opinions on absolute risk in a lifetime were more varied: while the largest group pre-test (N = 712, 48%) thought that their risk was not more than 5%, the largest group post-test (N = 657, 44%) expected theirs to be higher than 25% (Figure 2b). There was a statistically significant change ($p < 0.001$) for both items of perceived risk between baseline and follow-up, with 319 (21%) and 389 (26%) participants thinking that their chances of OAC had increased for comparative and absolute risk, respectively (Supplementary Tables 4 and 5).

Some interview participants did not demonstrate a good understanding of the relationship between reflux, BE and OAC, which may have led them to different interpretations of questions about personal risk of BE and OAC, and the size of their risk:

> *"…when I was searching for the probability, the ratio of Barrett's to actual oesophageal cancer, I seemed to be getting different answers."*
> *[age 70-79, positive test result]*

Some participants found the information about risk in the invitation leaflet difficult to understand but drew attention to the important role that the nurses played in explaining this to them at the start of the appointment.

> *"Because I'd never heard of Barrett's before,... obviously it was on the leaflets I read that, but when I actually come for the test the nurse that I saw... she explained it all to me and… how that can be a sign that you may get the cancerous cells and things like that. So yeah, it was very interesting. I didn't know that."*
> *[age 60-69, inadequate test result]*

DISCUSSION

Summary of main findings

This mixed-methods study evaluated patients' experiences of, and satisfaction with, the Cytosponge test in primary care as part of the BEST3 trial. Overall, participants were satisfied with their experience of the Cytosponge: they found it very convenient to attend their appointment at their own general practice and rated the staff interpersonal skills and competence very highly. Regarding the Cytosponge procedure itself, 87% of participants did not find swallowing very uncomfortable, while 60% reported gagging during the Cytosponge withdrawal in the questionnaire data; despite that, more than 80% were willing to have the test again or to recommend it to others. In interviews, patients provided more detailed descriptions of their experience, specifically different levels of pain and scratching resulting in a sore throat. Questionnaire data found a slight decrease in anxiety levels between before and after the test, and interviews helped identify patients' underlying motivations for feeling anxious: their ability to swallow, participating in a clinical trial, and waiting for test results. Lastly, we observed a statistically significant change in perceived risk of OAC pre- and post-test with 21% to 26% (depending on the risk type) of participants rating their risk as higher at follow-up. Interview data suggested that information about risk in the invitation leaflet was difficult to interpret for some patients and that nurses played an important role in providing more information on risk at the appointment, despite participants still not having a good understanding of the relationship between reflux, BE and OAC.

Interpretation

This study has provided a deeper understanding of those aspects of the Cytosponge test that worked well in the trial and would need to be maintained to ensure acceptability during implementation. First, the nurses

For peer review only - http://bmjopen.bmj.com/site/about/guidelines.xhtml

administering the Cytosponge were rated as supportive, knowledgeable and reassuring. Despite experiencing different levels of discomfort, most participants found that the pain was as expected, suggesting that it is important to explain how the Cytosponge is removed, that removal is brief, and that some discomfort may be necessary for the sponge to effectively gather cells. This may help patients to manage their expectations. Second, delivering the Cytosponge near home was perceived as convenient and acceptable. Third, staff technical competence was also rated very highly. Implementation of the Cytosponge test as a routine diagnostic test in primary care will need to ensure balance between convenience and adequate staff training.

Some aspects of the Cytosponge test were rated less well and our interview data provide insights into what could be changed. First, although the majority of participants (95%) were able to successfully swallow the device, swallowing and retrieval of the Cytosponge were less highly rated. To ensure a good overall experience continues when implementing the Cytosponge in primary care, it will be important to provide high-quality information and manage patient expectations of the physical experience, as was done in BEST3. Second, the median STAI-6 scores observed before the procedure and at follow-up were both well under the predefined level of clinically significant anxiety of 40 in the average adult population in a non-clinical setting.[21] However, some interviewees reported varying levels of pre-test anxiety linked to concerns about swallowing the device and general fear of the unknown; this might improve once patients are more familiar with the Cytosponge. While these findings are broadly reassuring, efforts should be made to ensure patients know what to expect and are supported if they feel anxious. Third, at both time points, the majority of participants rated their risk of OAC as being average for people of their age, showing some evidence of the 'optimistic bias' often observed in measures of comparative risk. At follow-up, a greater proportion of people rated their risk as being above average, which may reflect a greater awareness of the association between reflux, BE and OAC following the procedure. However, the qualitative data point to an inconsistent understanding of the relationships between these three conditions, which suggests room for improvement of the explanations given to patients.

Context of other literature

Previously, acceptability of the Cytosponge test had been assessed using a visual analogue scale ranging from 0 (worst) to 10 (best experience).[9, 10, 25] A review of five studies assessing the Cytosponge test found a satisfactory overall acceptability, with a median score of 6.[13] In addition, the BEST1 study showed, using the STAI-6, that anxiety levels were low before and after the test with similar scores obtained as in this current study.[9] One qualitative study has investigated the acceptability of Cytosponge, but the participants had not actually taken the test, so their attitudes were hypothetical.[26] It showed that acceptability was high despite initial concerns about swallowing and extracting the capsule.

Even though BEST3 participants experienced different levels of discomfort or pain during the swallowing and removal stages of the procedure, in most cases this would not discourage them from having the test again or recommending it to someone else. This is relevant in the context of implementing the Cytosponge as a routine test. Interestingly, studies investigating barriers to screening attendance found varying degrees of association between pain and re-attendance, with 25-46% of women citing pain of having a mammography as a reason for non-attendance;[27] however, worry about pain was not associated with low intention to re-attend cervical screening.[28]

Strengths and limitations

This study was undertaken within a large pragmatic randomised controlled trial, in which 1750 patients attended the Cytosponge appointment. Key strengths are that the BEST3 trial was set in primary care, where Cytosponge implementation is planned, and that this study used a mixed-methods approach. The findings from the IAPS, STAI-6 and perceived risk questionnaires, completed by nearly 1500 participants, were explored in more depth during interviews with a diverse sample of 30 patients, which included patients with unsuccessful swallows whose experience had otherwise not been captured in the follow-up questionnaire. The qualitative analyses, supported by a conceptual framework, offered detailed insights of participants' experiences and enriched the interpretation of the quantitative findings.

For peer review only - http://bmjopen.bmj.com/site/about/guidelines.xhtml

This study had limitations. Some attendees (<10%) did not return the follow-up questionnaire, and there were some small statistically significant differences in the distribution of patients' characteristics in those completing vs not completing the follow-up questionnaire. The IAPS, which had been adapted from flexible sigmoidoscopy, was only validated by piloting with a small number of patients, but the Cronbach's alpha of 0.83 indicates appropriate internal reliability of the adaptation of the questionnaire to the Cytosponge test. The predefined threshold of clinical anxiety (over 40) used in our analysis for the STAI-6 was defined in the literature for a non-clinical setting and for the complete STAI questionnaire.[21] The main limitation of the qualitative findings was that, for some, more than six weeks elapsed from a participant's Cytosponge procedure and their interview. This may have affected recall, although most participants were able to remember their experiences in substantial detail.

Conclusion

This study, exploring patients' experiences of, and satisfaction with, the Cytosponge test used extensive questionnaire and in-depth interview data. Overall, participants were satisfied with their experiences and we did not observe excess anxiety due to the procedure. Identifying aspects of the procedure which are currently working well or rated less positively will enable specific improvements to communications with patients that will result in a better experience once the Cytosponge test is implemented in clinical care.

For peer review only

TABLES

Table 1. Patient characteristics and GORD Impact Scale for the three subgroups of participation: attended the Cytosponge appointment and completed the baseline questionnaire *('attenders')*; completed the follow-up questionnaire *('follow-up responders')*; or interviewed *('interviewees')*.

	Completed baseline questionnaire *('attenders')* (N = 1750)		Completed follow-up questionnaire *('follow-up responders')* (N = 1488)		Interviewed *('interviewees')* (N = 30)		p-values for chi-squared test between *'follow-up responders'* (N = 1488) and *'attenders'* who are not *'follow-up responders'* (N = 262)
	N	%	N	%	N	%	
**Sex**							
Female	919	53%	782	52%	15	50%	0.985
Male	830	47%	706	47%	15	50%	
Missing	1	<1%	0	0%	0	0%	
**Age group**							
50-59	345	20%	285	19%	4	13%	*0.028
60-69	596	34%	497	33%	11	35%	
70-79	647	37%	572	38%	9	29%	
80-99	161	9%	134	9%	6	19%	
Missing	1	<1%	0	0%	1	3%	
**Cytosponge-TFF3 outcome (after repeat test)**							
TFF3 negative	1252	72%	1126	76%	14	47%	^0.246
TFF3 positive	231	13%	213	14%	10	33%	
Inadequate (equivocal/low-confidence negative/technical or processing failure)	171	10%	149	10%	2	7%	
Unsuccessful swallow	96	5%	0	0%	4	13%	
**Underwent repeat Cytosponge test**							
No	1560	89%	1322	89%	25	83%	0.338
Yes	190	11%	166	11%	5	17%	
**Education level**							
School up to 15-16 years of age	712	41%	605	41%	16	53%	0.104
College or vocational school	537	31%	455	31%	8	27%	
Professional training beyond college, university graduate or postgraduate degree	480	27%	414	28%	4	13%	
Other or prefer not to say	21	1%	14	1%	2	7%	
**Waist-hip ratio**							
<0.90	685	39%	601	40%	11	37%	*0.010
0.90<0.99	686	39%	562	38%	10	33%	
0.99+	378	22%	324	22%	9	30%	
Missing	1	<1%	1	<1%	0	0%	
**Comorbidities**							
No	228	13%	182	12%	6	20%	*0.018
Yes	1522	87%	1306	88%	24	80%	
**Medication duration**							
Less than 5 years	518	30%	431	29%	7	23%	0.166
More than 5 years	1232	70%	1057	71%	23	77%	
**Diagnoses**							

For peer review only - http://bmjopen.bmj.com/site/about/guidelines.xhtml

No Barrett's oesophagus	1618	92%	1367	92%	26	87%	0.118
Barrett's oesophagus – without dysplasia	117	7%	106	7%	4	13%	
Barrett's oesophagus – with dysplasia	11	1%	11	1%	0	0%	
Oesophageal adenocarcinoma (stage 1)	4	<1%	4	<1%	0	0%	
							**p-values for t-test between *'follow-up responders'* (N = 1488) and *'attenders'* who are not *'follow-up responders'* (N = 262)**
**GORD Impact Scale – Before taking acid-suppressant medications**							
Mean (SD)		1.9 (0.5)		1.9 (0.5)		1.9 (0.5)	0.319
No. missing		2		1		0	
**GORD Impact Scale – In the last week**							
Mean (SD)		1.3 (0.4)		1.3 (0.4)		1.3 (0.5)	0.451
No. missing		0		0		0	

* $p < 0.05$
^ Comparison excluding participants producing an unsuccessful swallow as they were not invited to fill in a follow-up questionnaire.

TFF3 = trefoil factor 3, GORD = gastro-oesophageal reflux disease, SD = standard deviation

For peer review only

Table 2. Number and proportion of participants (N = 1488) by ratings for the 22 questions of the inventory to assess patient satisfaction.

	Disagree		Neither		Agree		Missing	
	N	%	N	%	N	%	N	%
**Convenience and accessibility**								
I did not feel that I had to wait too long.*	42	3%	24	2%	1395	94%	28	2%
The test is in a place that is easy for me to get to.	90	6%	4	<1%	1389	93%	5	<1%
I did not find it hard to find a convenient time to come to the test.*	71	5%	15	1%	1368	92%	34	2%
**Staff interpersonal skills**								
I felt free to ask the staff questions I wanted to ask.	23	2%	1	<1%	1456	98%	8	1%
The staff did not seem to hurry me through too quickly.*	9	1%	2	<1%	1454	98%	23	2%
The staff did not use words that were hard to understand.*	22	1%	10	1%	1425	96%	31	2%
**Perceived technical competence**								
The nurse or member of staff was not too rough when performing the Cytosponge test.*	20	1%	14	1%	1422	96%	32	2%
I feel confident that the Cytosponge test was performed properly.	86	6%	10	1%	1384	93%	8	1%
**Swallowing of the capsule**								
I did not have to gag when I swallowed the Cytosponge capsule.*	373	25%	53	4%	1020	69%	42	3%
Swallowing the Cytosponge capsule was more comfortable than I expected.	221	15%	169	11%	1073	72%	25	2%
Swallowing the Cytosponge capsule did not cause me great discomfort.*	82	6%	60	4%	1300	87%	46	3%
**Waiting with capsule in stomach**								
I did not have to gag while I waited with the Cytosponge capsule in my stomach.*	146	10%	36	2%	1264	85%	42	3%
Waiting with the Cytosponge capsule in my stomach was more comfortable than I expected.	123	8%	133	9%	1207	81%	25	2%
Waiting with the Cytosponge capsule in my stomach did not cause me great discomfort.*	39	3%	36	2%	1365	92%	48	3%
**Pulling of the Cytosponge**								
I did not have to gag when the Cytosponge was pulled up.*	889	60%	68	5%	491	33%	40	3%
Pulling up of the Cytosponge was more comfortable than I expected.	354	24%	234	16%	866	58%	34	2%
Pulling up of the Cytosponge did not cause me great discomfort.*	193	13%	108	7%	1134	76%	53	4%
**Expectations and beliefs**								
I was not very anxious about having the Cytosponge test.*	296	20%	132	9%	1029	69%	31	2%
Undergoing the Cytosponge test will benefit my health.	27	2%	281	19%	1153	77%	27	2%
**General satisfaction**								
I was very satisfied with the care I received.	16	1%	2	<1%	1450	97%	20	1%
I would recommend the Cytosponge test to my friends.	38	3%	184	12%	1236	83%	30	2%
I would be willing to have another test if necessary.<br>As part of the Trial, you may still be contacted for a repeat Cytosponge test.	48	3%	229	15%	1185	80%	26	2%

* Items referring to negative aspects of patient experience were rephrased for this table using negative constructs to facilitate comparison between items. Changes are underlined.

For peer review only

For peer review only - http://bmjopen.bmj.com/site/about/guidelines.xhtml

AUTHOR CONTRIBUTIONS

GR and FMW conceptualised and JO, GR and FMW designed this mixed-methods study as nested within the BEST3 trial. FS conducted the patient interviews. RM, JB and FS acquired and analysed the data. RM, JB, JO, SGS, JW, PDS and FMW interpreted the data. RM, JB, JO and FMW drafted the manuscript. PDS provided statistical support. All authors critically reviewed the manuscript and approved the final version.

FUNDING

The laboratory of RCF is funded by a Core Programme Grant from the Medical Research Council (RG84369). The BEST3 study was primarily funded by Cancer Research UK (C14478/A21047). The National Institute for Health Research (NIHR) covered service support costs and National Health Service commissioners funded excess treatment costs, while Medtronic funded Cytosponge devices and TFF3 antibodies. This research was supported by the NIHR Cambridge Biomedical Research Centre (BRC-1215-20014). Cancer Research UK provide funding to the Cancer Prevention Trials Unit (C8162/A25356) and Cancer Prevention Group at King's College London (C8162/A16892). SGS is supported by a Yorkshire Cancer Research Fellowship. JW is funded by a Cancer Research UK career development fellowship (C7492/A17219). The funders had no role in study design, data collection, data analysis, data interpretation, or writing of the report.

ETHICAL APPROVAL

Ethical approval for the BEST3 trial was obtained from the East of England – Cambridge East Research Ethics Committee (Trial Registration ISRCTN68382401). All participants gave informed consent before any individual-level patient data were collected and any clinical procedure was done. In addition, interview participants provided written consent before participating in interviews.

ACKNOWLEDGMENTS

This research is linked to the CanTest Collaborative, which is funded by Cancer Research UK (C8640/A23385), of which FMW is Director and GR is Chair of the Steering Committee. We would also like to acknowledge patient input from Heartburn Cancer UK, and the contributions of our CanTest PPI lead, Mrs Margaret Johnson.

We acknowledge the contribution of statistician Irene Kaimi, who had a leading role early in the trial but who tragically died before the study was completed. We thank all of the sites and patients who participated in the BEST3 trial, without whom this research would not have been possible.

COMPETING INTERESTS

RCF is named on patents related to the Cytosponge-trefoil factor 3 test. Covidien GI Solutions (now Medtronic) licensed the Cytosponge from the Medical Research Council, and the device has now received the CE mark and is cleared by the US Food and Drug Administration. RCF is a shareholder in Cyted, a company working on early detection technology. PS reports fees paid to his organisation from GRAIL, outside of the submitted work. The remaining authors have no conflicts of interest to declare.

DATA SHARING STATEMENT

The trial protocol, statistical analysis plan, and statistical report will be available via the University of Cambridge data repository (https://www.data.cam.ac.uk/repository). Datasets will be available from the authors on request.

For peer review only - http://bmjopen.bmj.com/site/about/guidelines.xhtml

REFERENCES

1. Cancer Research UK. Oesophageal cancer statistics. Available from: https://www.cancerresearchuk.org/health-professional/cancer-statistics/statistics-by-cancer-type/oesophageal-cancer (Accessed 30th December 2020).
2. Cancer Research UK. Barrett's oesophagus. Available from: https://www.cancerresearchuk.org/about-cancer/other-conditions/barretts-oesophagus/about-barrett%27s (Accessed 30th December 2020).
3. Zeki S, Fitzgerald RC. Targeting care in Barrett's oesophagus. *Clinical Medicine* 2014;14(Suppl 6):s78-s83. doi: 10.7861/clinmedicine.14-6-s78.
4. Locke GR, 3rd, Talley NJ, Fett SL, et al. Prevalence and clinical spectrum of gastroesophageal reflux: a population-based study in Olmsted County, Minnesota. *Gastroenterology* 1997;112(5):1448-56. doi: 10.1016/s0016-5085(97)70025-8.
5. National Institute for Health and Care Excellence (NICE). Gastro-oesophageal reflux disease and dyspepsia in adults: investigation and management. NICE guideline.
6. NHS England. 2018/19 National Cost Collection data. Available from: https://www.england.nhs.uk/national-cost-collection/#ncc1819 (Accessed 4th January 2021).
7. Goudra B, Nuzat A, Singh PM, et al. Association between Type of Sedation and the Adverse Events Associated with Gastrointestinal Endoscopy: An Analysis of 5 Years' Data from a Tertiary Center in the USA. *Clin Endosc* 2017;50(2):161-69. doi: 10.5946/ce.2016.019.
8. Catlow J, Beaton D, Beintaris I, et al. JAG/BSG national survey of UK endoscopy services: impact of the COVID-19 pandemic and early restoration of endoscopy services. *Frontline Gastroenterology* 2020:flgastro-2020-101582. doi: 10.1136/flgastro-2020-101582.
9. Kadri SR, Lao-Sirieix P, O'Donovan M, et al. Acceptability and accuracy of a non-endoscopic screening test for Barrett's oesophagus in primary care: cohort study. *BMJ* 2010;341:c4372. doi: 10.1136/bmj.c4372.
10. Ross-Innes CS, Debiram-Beecham I, O'Donovan M, et al. Evaluation of a Minimally Invasive Cell Sampling Device Coupled with Assessment of Trefoil Factor 3 Expression for Diagnosing Barrett's Esophagus: A Multi-Center Case–Control Study. *PLOS Medicine* 2015;12(1):e1001780. doi: 10.1371/journal.pmed.1001780.
11. Benaglia T, Sharples LD, Fitzgerald RC, et al. Health benefits and cost effectiveness of endoscopic and nonendoscopic cytosponge screening for Barrett's esophagus. *Gastroenterology* 2013;144(1):62-73.e6. doi: 10.1053/j.gastro.2012.09.060.
12. Heberle CR, Omidvari AH, Ali A, et al. Cost Effectiveness of Screening Patients With Gastroesophageal Reflux Disease for Barrett's Esophagus With a Minimally Invasive Cell Sampling Device. *Clin Gastroenterol Hepatol* 2017;15(9):1397-404.e7. doi: 10.1016/j.cgh.2017.02.017.
13. Januszewicz W, Tan WK, Lehovsky K, et al. Safety and Acceptability of Esophageal Cytosponge Cell Collection Device in a Pooled Analysis of Data From Individual Patients. *Clin Gastroenterol Hepatol* 2019;17(4):647-56.e1. doi: 10.1016/j.cgh.2018.07.043.
14. Iqbal U, Siddique O, Ovalle A, et al. Safety and efficacy of a minimally invasive cell sampling device ('Cytosponge') in the diagnosis of esophageal pathology: a systematic review. *Eur J Gastroenterol Hepatol* 2018;30(11):1261-69. doi: 10.1097/meg.0000000000001210.
15. Fitzgerald RC, di Pietro M, O'Donovan M, et al. Cytosponge-trefoil factor 3 versus usual care to identify Barrett's oesophagus in a primary care setting: a multicentre, pragmatic, randomised controlled trial. *Lancet* 2020;396(10247):333-44. doi: 10.1016/s0140-6736(20)31099-0.
16. Offman J, Muldrew B, O'Donovan M, et al. Barrett's oESophagus trial 3 (BEST3): study protocol for a randomised controlled trial comparing the Cytosponge-TFF3 test with usual care to facilitate the diagnosis of oesophageal pre-cancer in primary care patients with chronic acid reflux. *BMC Cancer* 2018;18(1):784. doi: 10.1186/s12885-018-4664-3.

For peer review only - http://bmjopen.bmj.com/site/about/guidelines.xhtml

17. Jones R, Coyne K, Wiklund I. The gastro-oesophageal reflux disease impact scale: a patient management tool for primary care. *Aliment Pharmacol Ther* 2007;25(12):1451-9. doi: 10.1111/j.1365-2036.2007.03343.x.
18. Marteau TM, Bekker H. The development of a six-item short-form of the state scale of the Spielberger State-Trait Anxiety Inventory (STAI). *Br J Clin Psychol* 1992;31(3):301-6. doi: 10.1111/j.2044-8260.1992.tb00997.x.
19. Lerman C, Trock B, Rimer BK, et al. Psychological side effects of breast cancer screening. *Health Psychol* 1991;10(4):259-67. doi: 10.1037//0278-6133.10.4.259.
20. Schoen RE, Weissfeld JL, Bowen NJ, et al. Patient satisfaction with screening flexible sigmoidoscopy. *Arch Intern Med* 2000;160(12):1790-6. doi: 10.1001/archinte.160.12.1790.
21. Balsamo M, Cataldi F, Carlucci L, et al. Assessment of anxiety in older adults: a review of self-report measures. *Clin Interv Aging* 2018;13:573-93. doi: 10.2147/cia.S114100.
22. Stata Statistical Software: Release 15 [program]. College Station, TX: StataCorp LLC.
23. Ritchie J, Lewis C, McNaughton Nicholls C, et al. Qualitative Research Practice (Second Edition). London, UK: SAGE Publications Ltd. 2014.
24. Sekhon M, Cartwright M, Francis JJ. Acceptability of healthcare interventions: an overview of reviews and development of a theoretical framework. *BMC Health Services Research* 2017;17(1):88. doi: 10.1186/s12913-017-2031-8.
25. Katzka DA, Geno DM, Ravi A, et al. Accuracy, safety, and tolerability of tissue collection by Cytosponge vs endoscopy for evaluation of eosinophilic esophagitis. *Clin Gastroenterol Hepatol* 2015;13(1):77-83.e2. doi: 10.1016/j.cgh.2014.06.026.
26. Freeman M, Offman J, Walter FM, et al. Acceptability of the Cytosponge procedure for detecting Barrett's oesophagus: a qualitative study. *BMJ Open* 2017;7(3):e013901. doi: 10.1136/bmjopen-2016-013901.
27. Whelehan P, Evans A, Wells M, et al. The effect of mammography pain on repeat participation in breast cancer screening: a systematic review. *Breast* 2013;22(4):389-94. doi: 10.1016/j.breast.2013.03.003.
28. Waller J, Bartoszek M, Marlow L, et al. Barriers to cervical cancer screening attendance in England: a population-based survey. *J Med Screen* 2009;16(4):199-204. doi: 10.1258/jms.2009.009073.

For peer review only

For peer review only - http://bmjopen.bmj.com/site/about/guidelines.xhtml

FIGURE LEGENDS

Figure 1. Findings from questionnaires and patient interviews according to the themes of the Inventory to Assess Patient Satisfaction.

(a) Summary of findings

(b) Example interview quotes illustrating the practical elements of the Cytosponge procedure

Aspect of Cytosponge procedure	Example interview quotes
Convenience and accessibility	*Convenient alternative to a procedure in secondary care:*<br>"[…] from what [the nurse] was saying to me is that [the Cytosponge procedure] takes away that waiting for a hospital appointment, that you can have it done in the [GP] surgery, and if it was me again and, I don't know, something was not quite right, I wouldn't hesitate at coming down and having that done. Not at all, not at all."<br>*(age 60-69, inadequate test result at first appointment)*
Staff interpersonal skills	*Positive interpersonal skills:*<br>"The [nurse] who actually did it was really lovely. She really was. She was very calm, very in control and we chatted about different things and she was about to get married and all this sort of thing and it was, we learned a bit about each other, which was absolutely fine […] [The procedure is] done very nicely, lovely people, nothing to worry about, go and get it done."<br>*(age 70-79, negative test result)*<br>*Procedure explained clearly:*<br>""I mean if I didn't understand then I asked to explain it. I think [the staff] were very helpful and very nice, the way they put things over. I mean there was the two of them here and what one didn't answer, the other one did. No, I think they were very helpful and very kind."<br>*(age 80 and over, failed swallow)*
Perceived technical competence of staff	*Staff were skilled at removing sponge:*<br>"No, it was fine, it was just that and she did really well, she [removed the Cytosponge] as quick as she could be, obviously she had to go slower to get what she needed."<br>*(age 50-59, negative test result)*<br>*Patients and inexperienced staff may need more guidance:*<br>"...the first part of the extraction [of the Cytosponge] was fairly non-event[ful] but then again it did get stuck a bit in my throat […] And the [practice] nurse had to ask the [research] nurse […] she just said pull harder. So she pulled harder and it popped out. […] So I don't know if positioning the throat in a different way or me being told to hold the throat in a particular angle may have helped but, I mean, I know that sword swallowers, they hold their throat quite straight […] But there was no advice as to how to hold your head or position your throat and I thought that might have been useful […] Well to hold the head in a particular position and relax may have helped, I don't know, it may have got stuck whatever."<br>*(age 70-79, positive test result)*
Swallowing of the Cytosponge	*Difficulties due to string and retching:*<br>"The first time, when I swallowed it, the string seemed to flick around in the back of my throat and it didn't go down properly, so I was trying to add a bit more water and that, but I couldn't […] I was just retching all the time and I couldn't even get […] the water in my mouth because I just kept retching all the time […] And then the second time, it went straight down, straight down. It was marvellous, it went straight down and I thought, oh, I've cracked it, so I just kept sipping, and then all of a sudden I think a bit of the string... Like I felt down at the side, and I just went uh, and it just came straight out, just all came straight out altogether. […] I think it's the water I drank, it was still lying on my stomach and just brought it straight back up."<br>*(age 50-59, failed swallow)*<br>*Swallowing was easy:*<br>"That swallowing the capsule was simple, there was no... it was easy, it was just a matter of a few mouthfuls of water and that was it."<br>*(age 50-59, positive test result)*
Waiting with Cytosponge instomach	*Waiting was acceptable, especially when there were distractions:*<br>"But it wasn't horrendous and for the time that I was there and, you know, and by the time I'd sort of swallowed [the Cytosponge] and answered a few questions, had a little chat and drank some water, it was time for it to come up."<br>*(age 60-69, negative test result)*<br>*Mild discomfort:*<br>"You're aware of the string being in the throat […] It was slightly uncomfortable […] It was making you want to [cough] […] [but] There was no problem with it."<br>*(age 60-60, negative test result)*
Pulling of the Cytosponge	*Experience of pain:*<br>"It was painful. It was worse than I was expecting […] the nurse explained it to me afterwards, because afterwards I said to her, I said wow, that was more painful than I was expecting, and she explained that where your muscles will work to push things downwards, obviously, she said, when you're pulling the sponge up you're going completely against everything that it's doing and, do you know, I couldn't even describe what sort of pain it was, but it was literally... well it felt like a sponge literally was pulling out, you know, but […] I have to say, it only lasted a few seconds, and once it was out I suppose I had a tickly throat for the rest of the day. Not hurting, just a bit scratchy, tickly, certainly no painkillers, nothing like that. It was just those few seconds of it actually coming out wasn't pleasant, no. […] I did come back again [for second appointment following inadequate test result]."<br>*(age 60-69, inadequate test result)*

For peer review only - http://bmjopen.bmj.com/site/about/guidelines.xhtml

	Discomfort from gagging/coughing:<br>"It was all over in a matter of seconds, but it was when it hit the back of my throat, I did gag, and I started to cough or I had a coughing fit after it was out, I was red hot, you know, I think it was just with gagging, yeah, but it was fine, it was just something that lasted a matter of two seconds."<br>*(age 50-59, positive test result)*<br>*No discomfort:*<br>"It was over and done within a matter of… […] Woosh, gone. […] Finished, I didn't even feel it coming out."<br>*(age 60-69, negative test result)*

Figure 2. Ratings for perceived risk of oesophageal cancer at the Cytosponge appointment (baseline) and 7-14 day follow-up for participants completing both questionnaires (N = 1488). (a) Risk compared to someone of the same age (comparative risk). (b) Percent absolute risk.

Possible answers to the multiple-choice question on absolute percent risk were: 0%, 5%, 10%, 25%, 50%, 75% and 100%. Participants with missing answers at follow-up were included in the figures as they filled in other parts of the post-test questionnaire.

For peer review only

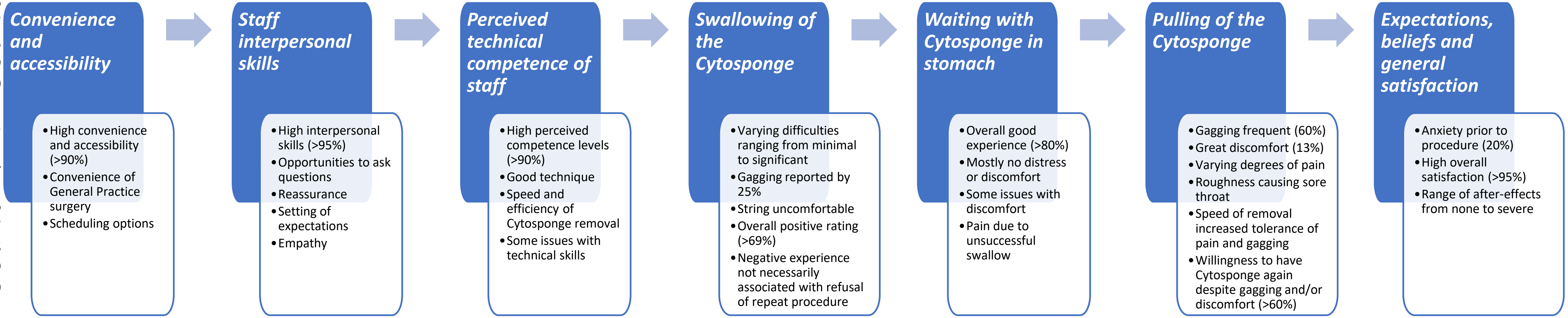
Convenience and accessibility
• High convenience and accessibility (>90%)
• Convenience of General Practice surgery
• Scheduling options
Staff interpersonal skills
• High interpersonal skills (>95%)
• Opportunities to ask questions
• Reassurance
• Setting of expectations
• Empathy
Perceived technical competence of staff
• High perceived competence levels (>90%)
• Good technique
• Speed and efficiency of Cytosponge removal
• Some issues with technical skills
Swallowing of the Cytosponge
• Varying difficulties ranging from minimal to significant
• Gagging reported by 25%
• String uncomfortable
• Overall positive rating (>69%)
• Negative experience not necessarily associated with refusal of repeat procedure
Waiting with Cytosponge in stomach
• Overall good experience (>80%)
• Mostly no distress or discomfort
• Some issues with discomfort
• Pain due to unsuccessful swallow
Pulling of the Cytosponge
• Gagging frequent (60%)
• Great discomfort (13%)
• Varying degrees of pain
• Roughness causing sore throat
• Speed of removal increased tolerance of pain and gagging
• Willingness to have Cytosponge again despite gagging and/or discomfort (>60%)
Expectations, beliefs and general satisfaction
• Anxiety prior to procedure (20%)
• High overall satisfaction (>95%)
• Range of after-effects from none to severe

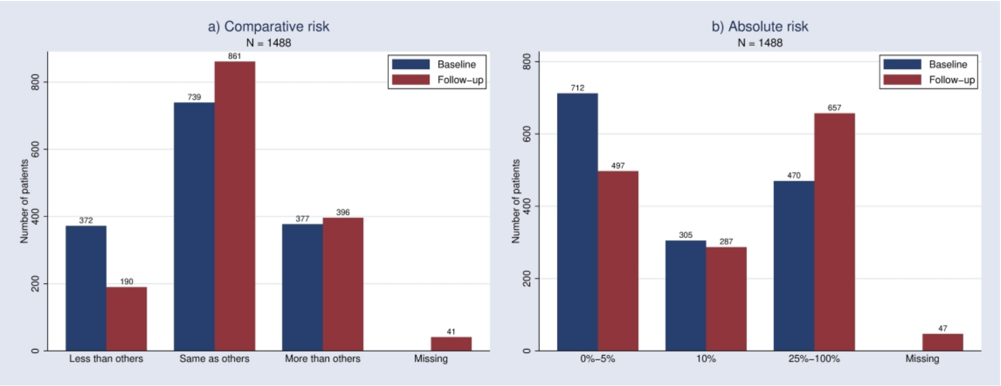

Figure 2. Ratings for perceived risk of oesophageal cancer at the Cytosponge appointment (baseline) and 7-14 day follow-up for participants completing both questionnaires (N = 1488). (a) Risk compared to someone of the same age (comparative risk). (b) Percent absolute risk.
Possible answers to the multiple-choice question on absolute percent risk were: 0%, 5%, 10%, 25%, 50%, 75% and 100%. Participants with missing answers at follow-up were included in the figures as they filled in other parts of the post-test questionnaire.

330x127mm (300 x 300 DPI)

Patient-reported experiences and views on the Cytosponge test: a mixed-methods analysis from the BEST3 trial

Roberta Maroni*, Jessica Barnes*, Judith Offman*, Fiona Scheibl, Samuel G Smith, Irene Debiram-Beecham, Jo Waller, Peter D Sasieni, Rebecca C Fitzgerald, Greg Rubin, BEST3 Consortium, Fiona M Walter

*Roberta Maroni, Jessica Barnes and Judith Offman equally contributed to this paper.

SUPPLEMENTARY MATERIALS

METHODS

Study design

The BEST3 trial [1] was a randomised controlled trial set in primary care with a mixed design (site-level and patient-level randomised) that enrolled 13,222 participants aged 50 or over with acid reflux symptoms ongoing for more than six months, identified via their general practice medical records. The primary endpoint was to compare the rate of diagnosis of Barrett's oesophagus between those offered the Cytosponge-trefoil factor 3 (TFF3) test and those on current management, and the results showed a ten-fold increase in being diagnosed with Barrett's oesophagus in the intervention arm compared with usual care.

The invitation letter (intervention arm only) was accompanied by an information leaflet on the Cytosponge. Participants expressing interest in receiving the test received a further information sheet with more details on the study and the Cytosponge. On the day of the test, participants were asked not to eat or drink anything in the four hours before the appointment. The appointment was held at participants' general practices and attendees (N = 1750) were offered an anaesthetic throat spray (optional) and water to drink to help ingest the capsule, following which 1654 (95%) patients produced a successful swallow. Furthermore, those producing a successful swallow but receiving an 'inadequate' test result (i.e. low-confidence negative TFF3, equivocal, or processing/technical failure) were invited to a repeat appointment when local resources and capacity allowed for that. All patients with a positive TFF3 result were referred for a confirmatory endoscopy, which was necessary to establish a diagnosis of Barrett's oesophagus or oesophageal cancer.

Data collection

Qualitative analysis

Face-to-face interviews were conducted by Fiona Scheibl, BA (Hons) and PhD, working at the time as Research Associate for the Department of Public Health and Primary Care at the University of Cambridge. FS has undergraduate and postgraduate training in social research and has spent 30 years in social and health care research in several universities in the UK. No relationship between FS and the interviewees was established prior to study commencement and the participants had no knowledge of the researcher's goals, except for what was reported in the Patient Information Leaflet, the invitation letters or the further information sheet sent by the BEST3 team, which set out all the aims and terms of the research project. The interview questions were provided by the authors and were pilot tested. No field notes were collected. Transcriptions of the audio recordings of the interviews were not returned to participants for comments or correction.

Analysis

Quantitative analysis

Questionnaire scoring

- Gastro-oesophageal reflux disease Impact Scale [2]: answers to each item were converted to scores on a four-point ordinal scale (1 = 'Never', 2 = 'Sometimes', 3 = 'Often', 4 = 'Daily') and then averaged to obtain each participant's final score.

For peer review only - http://bmjopen.bmj.com/site/about/guidelines.xhtml

- Shorter six-item form of the Spielberger State-Trait Anxiety Inventory (STAI-6) [3]: item scores on a four-point ordinal scale (1 = 'Not at all', 2 = 'Somewhat', 3 = 'Moderately', 4 = 'Very much') were reversed for positively worded questions and their sum was scaled so that the total score ranged from 20 to 80, as per the STAI guidelines.
- Perceived risk [5]: both risk compared to someone of the same age ('Much lower', 'Lower', 'Neither higher nor lower', 'Higher', 'Much higher') and absolute risk in a lifetime ('0%', '5%', '10%', '25%', '50%', '75%', '100%') are shown with some of the answer categories combined.
- Inventory to Assess Patient Satisfaction (IAPS) [6]: ratings categories ('Strongly agree', 'Agree', 'Not sure', 'Disagree', 'Strongly disagree') were combined ('Agree', 'Not sure', 'Disagree') and number and proportion of participants for each item are presented. For presentation purposes (Table 2), the text of items referring to negative aspects of the patient experience was rephrased using negative constructs to facilitate the visual comparison between items. Answers to the three items in the category "Pulling of the Cytosponge" were converted to scores on a 5-point ordinal scale (1 = 'Strongly agree', 2 = 'Agree', 3 = 'Not sure', 4 = 'Disagree', 5 = 'Strongly disagree'), which were then reversed for the two items referring to negative aspects. The three scores were then averaged for each patient to identify participants dissatisfied with the Cytosponge retrieval (i.e. score of 4 or above). Their ratings were cross-checked with the two inventory items referring to willingness to have the procedure again or to recommend it to friends.

Qualitative analysis

Researcher JB performed a thematic analysis on the interview data, with input from FW and JW. Data were organised and managed according to the Framework approach.[7] After familiarisation with the data through reading all the transcripts, JB developed an initial thematic framework of data labels. The aim with producing the initial set of labels was to enable effective data sorting and management – not to arrive at an exhaustive set of themes. This involved identifying an initial, broad set of labels that would be used to label and sort the data to enable the subsequent thematic analysis.

Labels were created inductively and deductively. Inductively-created labels were based on emergent concepts identified in the data. Deductively-created labels were based on the IAPS (as used in the quantitative questionnaire for the trial) [6] and the Theoretical Framework of Acceptability (TFA).[8] Use of the IAPS constructs as labels allowed us to more directly relate participant experience across qualitative and quantitative datasets. Use of the TFA constructs allowed us to examine additional dimensions of patient experience associated with acceptability that were not captured by IAPS.

Labels were discussed and reviewed by JB, FW and JW. JB then sorted the data by reading through each transcript and applying the labels cross-sectionally (i.e. the set of labels was applied across the entire set of transcripts where relevant). The labelled data was then transposed into the conventional Framework matrix, in which each label becomes a column and each participant/case becomes a row.

REFERENCES

1. Fitzgerald RC, di Pietro M, O'Donovan M, et al. Cytosponge-trefoil factor 3 versus usual care to identify Barrett's oesophagus in a primary care setting: a multicentre, pragmatic, randomised controlled trial. *Lancet* 2020;396(10247):333-44. doi: 10.1016/s0140-6736(20)31099-0.
2. Jones R, Coyne K, Wiklund I. The gastro-oesophageal reflux disease impact scale: a patient management tool for primary care. *Aliment Pharmacol Ther* 2007;25(12):1451-9. doi: 10.1111/j.1365-2036.2007.03343.x.
3. Marteau TM, Bekker H. The development of a six-item short-form of the state scale of the Spielberger State-Trait Anxiety Inventory (STAI). *Br J Clin Psychol* 1992;31(3):301-6. doi: 10.1111/j.2044-8260.1992.tb00997.x.
4. Kadri SR, Lao-Sirieix P, O'Donovan M, et al. Acceptability and accuracy of a non-endoscopic screening test for Barrett's oesophagus in primary care: cohort study. *BMJ* 2010;341:c4372. doi: 10.1136/bmj.c4372.
5. Lerman C, Trock B, Rimer BK, et al. Psychological side effects of breast cancer screening. *Health Psychol* 1991;10(4):259-67. doi: 10.1037//0278-6133.10.4.259.

For peer review only - http://bmjopen.bmj.com/site/about/guidelines.xhtml

For peer review only

6. Schoen RE, Weissfeld JL, Bowen NJ, et al. Patient satisfaction with screening flexible sigmoidoscopy. *Arch Intern Med* 2000;160(12):1790-6. doi: 10.1001/archinte.160.12.1790.
7. Ritchie J, Lewis C, McNaughton Nicholls C, et al. Qualitative Research Practice (Second Edition). London, UK: SAGE Publications Ltd. 2014.
8. Sekhon M, Cartwright M, Francis JJ. Acceptability of healthcare interventions: an overview of reviews and development of a theoretical framework. *BMC Health Services Research* 2017;17(1):88. doi: 10.1186/s12913-017-2031-8.

SUPPLEMENTARY TABLES

Supplementary Table 1. Sampling characteristics* of BEST3 participants being interviewed.

	No. of participants (N = 30)
**Geographic region in England**	
East	20
North-east	8
West	2
**Cytosponge-TFF3 outcome (at first appointment)**	
TFF3 negative	10
TFF3 positive	10
Inadequate (equivocal/low-confidence negative/technical or processing failure)**	6
Unsuccessful swallow	4
**Visual analogue scale acceptability rating (0-10)*** **	
5	2
6	1
7	1
8	4
9	4
10	9
Missing	9

*Also refer to Table 1 for the other sampling characteristics: age group and sex.
**Participants with an inadequate test result were invited to a repeat appointment when local resources and availability allowed for that.
***Visual analogue scale ratings were not used to ensure equal sampling of interviewees across scores, but rather to guarantee a diversity of experiences.

Supplementary Table 2. Understanding of test results, summarised by themes and quotes from patient interviews.

Cytosponge test result	Theme	Exemplar quotes
Positive	Sense of shock due to expecting negative result<br>Some participants experienced shock as they had expected a negative result based on their understanding of cancer in general (i.e. that it is caused by lifestyle factors such as drinking or smoking, or that it is hereditary) rather than an understanding of Barrett's oesophagus or oesophageal cancer.	*"I never thought any further than taking the test, really. Well, I mean I don't smoke, I don't drink so I didn't expect anything other than a clear."*<br>(age 80+, positive result)
	Sense of shock due to connotations of cancer more generally<br>Receiving a positive Cytosponge result was experienced to some degree as being like receiving a cancer diagnosis for some participants.	*"I think it was just a shock to hear that, you automatically… when I've read the leaflets and that and it's like Barrett's oesophagus is like looking for cancer, you just automatically always have that word in the back of your head, which I still have."*<br>(age 50-59, positive result)

For peer review only - http://bmjopen.bmj.com/site/about/guidelines.xhtml

	Sense of confusion or concern about test result meaning<br>Some participants receiving positive test results felt that the use of the word "positive" was difficult to understand, as they initially interpreted it in the lay sense of meaning "good".<br>Another cause of concern was about how to communicate the positive test result to family members. Some participants did not have an adequate understanding of what the test result meant to be able to explain it reassuringly to their family members.	*"I think saying positive is like saying you've got it. It would be like a possibility that needs further investigation or something like that."*<br>(age 50-59, positive result)<br><br>*"I suppose really it may have been better for possible where you get a positive is maybe sit there in front of the GP with your wife and then explain, because I had no idea what Barrett's was and you can look it up and it tells you all sorts of... and it's not the best way to look at anything, is it?"*<br>(age 50-59, positive result)
	No particularly strong reaction to positive result<br>Some participants did not react strongly to their result. In some cases, this was due to previous experiences that had given them relevant literacy or knowledge on cancer and cancer test results. In other cases, it was because the participant felt they had the necessary coping skills and attitudes, such as feeling there was no point in worrying, or that any problems can be managed or planned around.	*"... it didn't worry me, I had no problem. I want to know, end of, whatever you're going to throw at me, as long as I can plan it, that's how I live my life."*<br>(age 60-69, positive result)
Negative	Sense of relief<br>The negative result alleviated a sense of uncertainty or anxiety for some participants. This sometimes extended to a sense of relief on behalf of their families. In some cases, participants felt relieved to get confirmation that they were not on the same trajectory as family members who had previously suffered from issues related to reflux, or to get confirmation that their PPI medication had been effective.	Researcher: *Alright then, and did you have any emotional feelings about having that result at all, apart from relief?*<br>Participant: *No, just relief really. And the family as well.*<br>Interviewer: *Yeah.*<br>Participant: *Because I got to that stage now where my children think they should look after me, so it was a relief to them as well.*<br>(age 70-79, negative result)
	No particularly significant reaction, or a mildly positive reaction<br>This was sometimes due to participants simply having expected a negative result, while others simply had the attitude that there was no point in worrying.	*"I wasn't particularly bothered. There would be either something wrong or not"*<br>(age 70-79, negative result)
Inadequate (low confidence/ equivocal/ processing failure)	Understood the result<br>Some participants understood what this result meant and the reasons behind it and were willing to attend a second appointment for another procedure. They reported understanding that the reason for their result was that there were not enough cells collected.	*"The first time I didn't receive any notification. It was about two...just over two weeks, but that possibly was to do with the fact that they hadn't been able to take enough cells, and that notification was just to say that... apologising there wasn't enough cells, and would I mind coming back? And I said no, it's fine. And the second time I received a letter quite quickly, about seven to ten days afterwards, saying that the cells were all normal."*<br>(age 60-69, inadequate result)

For peer review only - http://bmjopen.bmj.com/site/about/guidelines.xhtml

	A sense of confusion about what the result meant<br>Some participants seemed unaware that this result was possible. They wondered why the test had not worked as expected and had not collected enough cells, and if theirs was the only case of this occurring. In some cases, this experience generated mistrust. This suggests that participants needed clearer information about how this result might come about and how common it is.	*"Well it did cross my mind that I wasn't being told the truth the first time. [...] I just wondered about it. [...] But I was assured that wasn't the case, I wasn't told anything that wasn't the truth. I was told there were too few cells collected."*<br>(age 70-79, inadequate result from first test, negative result from second test)

Supplementary Table 3. Overall STAI-6 score at Cytosponge-TFF3 appointment (baseline) and 7-14 day follow-up for participants completing both questionnaires.

**Overall STAI-6 score**		**At follow-up**				
		20<40	40<60	60-80	Missing	**Total**
**At baseline**	20<40	858	82	2	48	**990**
	40<60	300	142	12	21	**475**
	60-80	7	13	2	1	**23**
	**Total**	**1165**	**237**	**16**	**70**	**1488**

Supplementary Table 4. Perceived risk of oesophageal cancer compared to someone of the same age at Cytosponge-TFF3 appointment (baseline) and 7-14 day follow-up for participants completing both questionnaires.

**Relative risk of oesophageal cancer**		**At follow-up**					**p-value for McNemar's test (comparing "less than others" vs "more than others")**
		Less than others	Same as others	More than others	Missing	**Total**	
**At baseline**	Less than others	130	189	36	17	**372**	< 0.001
	Same as others	56	570	94	19	**739**	
	More than others	4	102	266	5	**377**	
	**Total**	**190**	**861**	**396**	**41**	**1488**	

Supplementary Table 5. Perceived percent risk of developing oesophageal cancer in a lifetime at Cytosponge-TFF3 appointment (baseline) and 7-14 day follow-up for participants completing both questionnaires.

**Percent absolute risk of oesophageal cancer**		**At follow-up**					**p-value for McNemar's test (comparing 0%, 5% vs 25%, 50%, 75%, 100%)**
		0%, 5%	10%	25%, 50%, 75%, 100%	Missing	**Total**	
**At baseline**	0%, 5%	396	108	181	27	**712**	< 0.001
	10%	70	123	100	12	**305**	
	25%, 50%, 75%, 100%	31	56	375	8	**470**	
	Missing	0	0	1	0	**1**	
	**Total**	**497**	**287**	**657**	**47**	**1488**	

For peer review only - http://bmjopen.bmj.com/site/about/guidelines.xhtml

SUPPLEMENTARY FIGURES

Supplementary Figure 1. (a) Trial flowchart for the patient-reported experience analysis of the BEST3 trial. (b) Venn diagram with the three subgroups of participation outlined in the analysis.

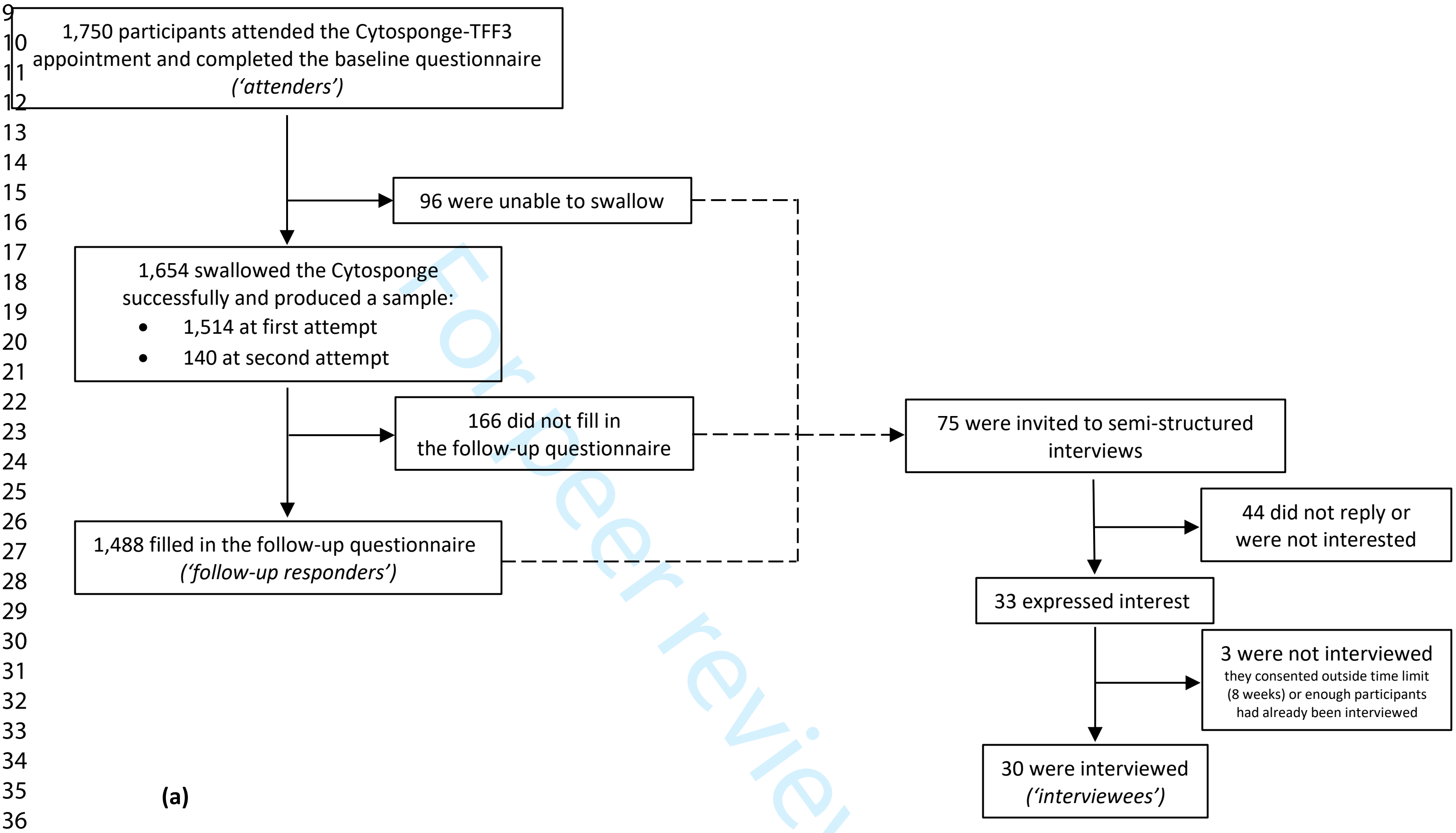

(a)

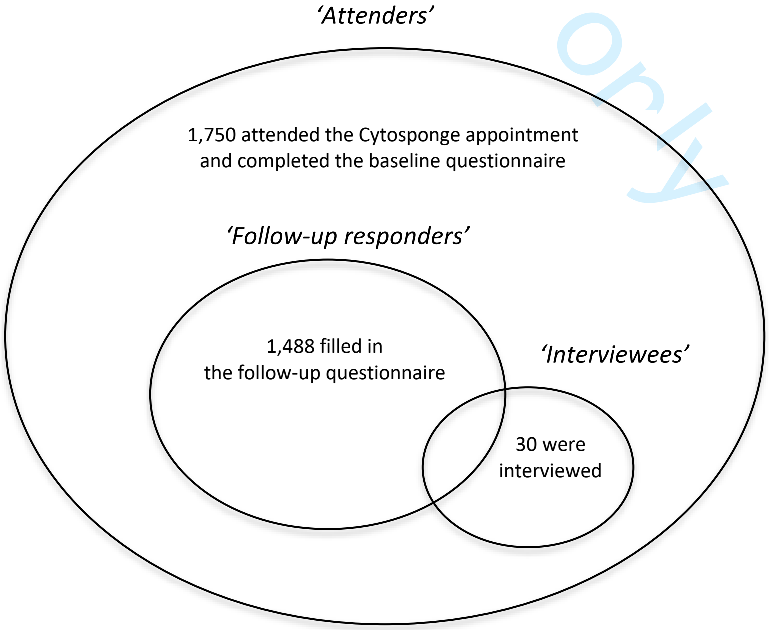

(b)

For peer review only - http://bmjopen.bmj.com/site/about/guidelines.xhtml

APPENDIX - QUESTIONNAIRES

A. BASELINE CLINICAL FORM

GENERAL INFORMATION

1. Height ………. cm
2. Weight ………. kg
3. What was your weight aged 20?
   - Underweight
   - Normal range
   - Overweight
   - Obese
   - Don't remember

4. Have you ever been obese?
   - Yes
   - No

5. Waist circumference ………. cm
6. Hip circumference ………. cm

MEDICATION

7. Medication type for reflux symptoms
   - H2 receptor antagonists
   - Proton pump inhibitor
   - Over the counter anti-acids
   - Other

7a. Name: ……….

7b. Dose: ……….

7c. Units:
- mg
- g
- ml
- tablet

7d. Frequency:
- OD
- BD
- TDS
- QDS
- PRN

7e. Month/year started:
- 0-1 year
- 1-2 years
- 3-4 years
- 4-5 years
- 5-6 years
- 6+ years

8. Please confirm whether patient has any comorbidities:
   - Yes
   - No

[COMORBIDITY TYPES COLLECTED IN SEPARATE SUBFORM]

SYMPTOMS

9. Gastro-oesophageal Reflux Disease Impact Scale (GIS) – part A

Please complete the following questions by marking one response per question. Consider your symptoms prior to you taking any acid suppressant medication. There are no right or wrong answers. Be sure to answer every question.

**Prior to you taking any acid suppressant medication please confirm the following…**	**DAILY**	**OFTEN**	**SOMETIMES**	**NEVER**
1. How often did you have the following symptoms:				
a. Pain in your chest or behind the breastbone?				
b. Burning sensation in your chest or behind the breastbone?				
c. Regurgitation or acid taste in your mouth?				
d. Pain or burning in your upper stomach?				
e. Sore throat or hoarseness that was related to your heartburn or acid reflux?				
2. How often did you have difficulty getting a good night's sleep because of your symptoms?				
3. How often did your symptoms prevent you from eating or drinking any of the foods you like?				
4. How frequently did your symptoms keep you from being fully productive in your job or daily activities?				

For peer review only - http://bmjopen.bmj.com/site/about/guidelines.xhtml

5.	How often did you buy over-the-counter medication (such as Rennies, Tums, Gaviscon)?				

10. Gastro-oesophageal Reflux Disease Impact Scale (GIS) – part B
Please complete the following questions by marking one response per question. Consider your symptoms over the past week. There are no right or wrong answers. Be sure to answer every question.

In the past week…		DAILY	OFTEN	SOMETIMES	NEVER
1.	How often have you had the following symptoms:				
	f. Pain in your chest or behind the breastbone?				
	g. Burning sensation in your chest or behind the breastbone?				
	h. Regurgitation or acid taste in your mouth?				
	i. Pain or burning in your upper stomach?				
	j. Sore throat or hoarseness that is related to your heartburn or acid reflux?				
2.	How often have you had difficulty getting a good night's sleep because of your symptoms?				
3.	How often have your symptoms prevented you from eating or drinking any of the foods you like?				
4.	How frequently have your symptoms kept your from being fully productive in your job or daily activities?				
5.	How often do you take additional medication other than what the physician told you to take (such as Rennies, Tums, Gaviscon)?				

<u>FURTHER INFORMATION</u>

11. How long ago did your heartburn first begin?
- Never
- Last 6 months
- 7 months to 1 year
- 1 to 2 years
- 2 to 5 years
- 5 to 10 years
- 10 to 20 years
- More than 20 years

12. How long ago did you first notice the acid/sour taste in your mouth?
- Never
- Last 6 months
- 7 months to 1 year
- 1 to 2 years
- 2 to 5 years
- 5 to 10 years
- 10 to 20 years
- More than 20 years

13. Have you been prescribed treatment for H.pylori?
- Yes
- No
- Don't know

14. Did the treatment for H.pylori make your symptoms:
- Worse
- No change
- Better

15. Are you taking medicine for your stomach symptoms?
- Yes
- No

For peer review only

B. BASELINE CLINICAL FORM

Lifestyle/family history

EDUCATION

1. What is the highest level of education that you have achieved?
   - School up to 15-16 years
   - College or vocational study
   - University graduate
   - Professional training beyond college or postgraduate degree
   - Other
   - Prefer not to say
2. If other, please specify ....

SMOKING

3. How many hours a day are you exposed to other people's smoke?
   - 0 hours
   - 1-6 hours
   - 6-12 hours
   - 12-18 hours
   - 18-24 hours
4. Have you ever smoked cigarettes, tobacco, pipe or cigars?
   - Yes
   - No
5. Age when you started smoking ....
6. Have you stopped smoking?
   - Yes
   - No
7. If you are no longer smoking, at what age did you stop? ....

How many/much did you or do you smoke per day of:

8. Cigarettes ....
9. Cigars ....
10. Tobacco (cigarettes/pipe) .... oz or grams

ALCOHOL HISTORY

11. Which one of the following best describes your present alcohol intake?
    - None
    - Daily or most days
    - Weekends only
    - Occasional (once / twice per month)
12. Which of the following is your preferred beverage(s)?
    - Red wine
    - White wine
    - Spirits
    - Beer
    - Alcopops
    - Other
    - Prefer not to say
13. If other, please specify ....
14. At present, how many units do you drink a week?
    - 1-5 units
    - 6-10 units
    - 11-15 units
    - 16-20 units
    - 21-25 units
    - 26-30 units
    - 30+ units
    - Not sure
    - Prefer not to say
15. Did you ever drink heavily in the past? (Heavy drinking is defined as >14 units per week for women and >21 units per week for men)
    - Yes
    - No
    - Not sure
    - Prefer not to say
16. How many units a week did you drink when you were 20?
    - 0 units
    - 1-5 units
    - 6-10 units
    - 11-15 units
    - 16-20 units
    - 21-25 units
    - 26-30 units
    - 30+ units
    - Not sure
    - Prefer not to say

For peer review only

For peer review only - http://bmjopen.bmj.com/site/about/guidelines.xhtml

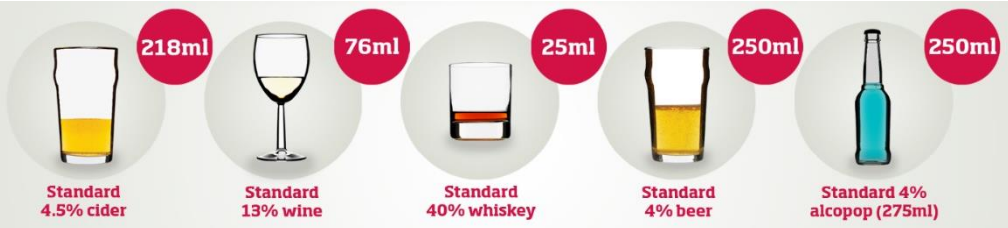

Courtesy of Public Health England

FAMILY HISTORY

17. Do any of your family have any of the following: heartburn, Barretts's oesophagus, cancer of the gullet/oesophagus, any other cancer and type.

- Yes
- No

18. (Please answer all questions for the relatives this is applicable for)

Relative	Heartburn	Barrett's oesophagus	Cancer of the gullet or oesophagus	Any other cancer and type

Perceived risk of developing oesophageal cancer

These questions are about how susceptible you feel to oesophageal cancer.

Compared to a person of the same age as you, what are your chances of developing oesophageal cancer? *(Please tick one)*	Much lower ☐<br>Lower ☐<br>Neither higher nor lower ☐<br>Higher ☐<br>Much higher ☐
In your lifetime, what do you consider your risk of developing oesophageal cancer is? *(Please tick one)*	0% ☐<br>5% ☐<br>10% ☐<br>25% ☐<br>50% ☐<br>75% ☐<br>100% ☐

Short-form of the state scale of the Spielberger State-Trait Anxiety Inventory (STAI-6)

A number of statements which people have used to describe themselves are given below. Read each sentence and then circle the most appropriate number to the right of the statement to indicate how you feel RIGHT NOW, AT THIS MOMENT. There are no right or wrong answers. Do not spend too much time on any one statement but give the answer which seems to describe your present feelings best.

(Please tick one box for each statement)

	Not at all	Somewhat	Moderately	Very much
**I feel calm**	1	2	3	4
**I am tense**	1	2	3	4
**I feel upset**	1	2	3	4
**I am relaxed**	1	2	3	4
**I feel content**	1	2	3	4
**I am worried**	1	2	3	4

For peer review only

C. 7-14 DAY FOLLOW-UP QUESTIONNAIRE

Inventory to Assess Patient Satisfaction (IAPS)

You recently received the Cytosponge™ test at your practice as part of the BEST3 Trial. On a scale of 1-5, please indicate whether you agree or disagree with the following statements:

(Please circle one response per statement)

	Strongly agree	Agree	Not sure	Disagree	Strongly disagree
**Convenience and accessibility**					
I felt that i had to wait too long.	1	2	3	4	5
The test is in a place that is easy for me to get to.	1	2	3	4	5
I found it hard to find a convenient time to come to the test.	1	2	3	4	5
**Staff interpersonal skills**					
I felt free to ask the staff questions i wanted to ask.	1	2	3	4	5
The staff seemed to hurry me through too quickly.	1	2	3	4	5
The staff used words that were hard to understand.	1	2	3	4	5
**Perceived technical competence**					
The nurse or member of staff was too rough when performing the Cytosponge test.	1	2	3	4	5
I feel confident that the Cytosponge test was performed properly.	1	2	3	4	5
**Swallowing of the capsule**					
I had to gag when I swallowed the Cytosponge capsule.	1	2	3	4	5
Swallowing the Cytosponge capsule was more comfortable than i expected.	1	2	3	4	5
Swallowing the Cytosponge capsule caused me great discomfort.	1	2	3	4	5
**Waiting with capsule in stomach**					
I had to gag while I waited with the Cytosponge capsule in my stomach.	1	2	3	4	5
Waiting with the Cytosponge capsule in my stomach was more comfortable than i expected.	1	2	3	4	5
Waiting with the Cytosponge capsule in my stomach caused me great discomfort.	1	2	3	4	5
**Pulling up of the Cytosponge**					
I had to gag when the Cytosponge was pulled up.	1	2	3	4	5
Pulling up of the Cytosponge was more comfortable than i expected.	1	2	3	4	5
Pulling up of the Cytosponge caused me great discomfort.	1	2	3	4	5
**Expectations and beliefs**					
I was very anxious about having the Cytosponge test.	1	2	3	4	5
Undergoing the Cytosponge test will benefit my health.	1	2	3	4	5
**General satisfaction**					
I was very satisfied with the care I received.	1	2	3	4	5
I would recommend the Cytosponge test to my friends.	1	2	3	4	5
I would be willing to have another if necessary.*	1	2	3	4	5

*As part of the Trial, you may still be invited for a repeat Cytosponge test.

For peer review only - http://bmjopen.bmj.com/site/about/guidelines.xhtml

Cytosponge test experience questionnaire

Please mark on the line your experience with the Cytosponge test:

0	5	10
Completely unacceptable	*Neither unacceptable nor acceptable*	*Completely acceptable*

Comments:

Perceived risk of developing oesophageal cancer

These questions are about how susceptible you feel to oesophageal cancer.

Compared to a person of the same age as you, what are your chances of developing oesophageal cancer? *(please tick one)*	Much lower ☐<br>Lower ☐<br>Neither higher nor lower ☐<br>Higher ☐<br>Much higher ☐
In your lifetime, what do you consider your risk of developing oesophageal cancer is? *(please tick one)*	0% ☐<br>5% ☐<br>10% ☐<br>25% ☐<br>50% ☐<br>75% ☐<br>100% ☐

Short-form of the state scale of the Spielberger State-Trait Anxiety Inventory (STAI-6)

A number of statements which people have used to describe themselves are given below. Read each sentence and then circle the most appropriate number to the right of the statement to indicate how you feel RIGHT NOW, AT THIS MOMENT. There are no right or wrong answers. Do not spend too much time on any one statement but give the answer which seems to describe your present feelings best.

(Please tick one box for each statement)

	Not at all	Somewhat	Moderately	Very much
I feel calm	1	2	3	4
I am tense	1	2	3	4
I feel upset	1	2	3	4
I am relaxed	1	2	3	4
I feel content	1	2	3	4
I am worried	1	2	3	4

For peer review only - http://bmjopen.bmj.com/site/about/guidelines.xhtml

COREQ (COnsolidated criteria for REporting Qualitative research) Checklist

A checklist of items that should be included in reports of qualitative research. You must report the page number in your manuscript where you consider each of the items listed in this checklist. If you have not included this information, either revise your manuscript accordingly before submitting or note N/A.

Topic	Item No.	Guide Questions/Description	Reported on Page No.
**Domain 1: Research team and reflexivity**			
Personal characteristics			
Interviewer/facilitator	1	Which author/s conducted the interview or focus group?	
Credentials	2	What were the researcher's credentials? E.g. PhD, MD	
Occupation	3	What was their occupation at the time of the study?	
Gender	4	Was the researcher male or female?	
Experience and training	5	What experience or training did the researcher have?	
Relationship with participants			
Relationship established	6	Was a relationship established prior to study commencement?	
Participant knowledge of the interviewer	7	What did the participants know about the researcher? e.g. personal goals, reasons for doing the research	
Interviewer characteristics	8	What characteristics were reported about the inter viewer/facilitator? e.g. Bias, assumptions, reasons and interests in the research topic	
**Domain 2: Study design**			
Theoretical framework			
Methodological orientation and Theory	9	What methodological orientation was stated to underpin the study? e.g. grounded theory, discourse analysis, ethnography, phenomenology, content analysis	
Participant selection			
Sampling	10	How were participants selected? e.g. purposive, convenience, consecutive, snowball	
Method of approach	11	How were participants approached? e.g. face-to-face, telephone, mail, email	
Sample size	12	How many participants were in the study?	
Non-participation	13	How many people refused to participate or dropped out? Reasons?	
Setting			
Setting of data collection	14	Where was the data collected? e.g. home, clinic, workplace	
Presence of non-participants	15	Was anyone else present besides the participants and researchers?	
Description of sample	16	What are the important characteristics of the sample? e.g. demographic data, date	
Data collection			
Interview guide	17	Were questions, prompts, guides provided by the authors? Was it pilot tested?	
Repeat interviews	18	Were repeat inter views carried out? If yes, how many?	
Audio/visual recording	19	Did the research use audio or visual recording to collect the data?	
Field notes	20	Were field notes made during and/or after the inter view or focus group?	
Duration	21	What was the duration of the inter views or focus group?	
Data saturation	22	Was data saturation discussed?	
Transcripts returned	23	Were transcripts returned to participants for comment and/or	

For peer review only

Topic	Item No.	Guide Questions/Description	Reported on Page No.
		correction?	
**Domain 3: analysis and findings**			
Data analysis			
Number of data coders	24	How many data coders coded the data?	
Description of the coding tree	25	Did authors provide a description of the coding tree?	
Derivation of themes	26	Were themes identified in advance or derived from the data?	
Software	27	What software, if applicable, was used to manage the data?	
Participant checking	28	Did participants provide feedback on the findings?	
Reporting			
Quotations presented	29	Were participant quotations presented to illustrate the themes/findings? Was each quotation identified? e.g. participant number	
Data and findings consistent	30	Was there consistency between the data presented and the findings?	
Clarity of major themes	31	Were major themes clearly presented in the findings?	
Clarity of minor themes	32	Is there a description of diverse cases or discussion of minor themes?	

Developed from: Tong A, Sainsbury P, Craig J. Consolidated criteria for reporting qualitative research (COREQ): a 32-item checklist for interviews and focus groups. *International Journal for Quality in Health Care*. 2007. Volume 19, Number 6: pp. 349 – 357

Once you have completed this checklist, please save a copy and upload it as part of your submission. DO NOT include this checklist as part of the main manuscript document. It must be uploaded as a separate file.

For peer review only

BMJ Open

Patient-reported experiences and views on the Cytosponge test: a mixed-methods analysis from the BEST3 trial

Journal:	*BMJ Open*
Manuscript ID	bmjopen-2021-054258.R1
Article Type:	Original research
Date Submitted by the Author:	28-Jan-2022
Complete List of Authors:	Maroni, Roberta; King's College London, Cancer Prevention Trials Unit (CPTU), Cancer Prevention Group, School of Cancer & Pharmaceutical Sciences<br>Barnes, Jessica; King's College London, Cancer Prevention Group, School of Cancer and Pharmaceutical Sciences<br>Offman, Judith; King's College London, Cancer Prevention Group, School of Cancer and Pharmaceutical Sciences<br>Scheibl, Fiona; University of East Anglia, Norwich Medical School<br>Smith, Samuel; University of Leeds, Leeds Institute of Health Sciences<br>Debiram-Beecham, Irene; University of Cambridge, MRC Cancer Unit, Hutchison-MRC Research Centre<br>Waller, Jo; King's College London, Cancer Prevention Group, School of Cancer and Pharmaceutical Sciences<br>Sasieni, Peter; King's College London, Cancer Prevention Group, School of Cancer and Pharmaceutical Sciences<br>Fitzgerald, RC; University of Cambridge, MRC Cancer Unit, Hutchison-MRC Research Centre; Cambridge University Hospitals NHS Foundation Trust<br>Rubin, Greg; Newcastle University, Population Health Sciences Institute<br>Walter, Fiona; University of Cambridge, The Primary Care Unit, Department of Public Health & Primary Care; Queen Mary University of London, Institute of Population Health Sciences
**Primary Subject Heading**:	Gastroenterology and hepatology
Secondary Subject Heading:	Qualitative research
Keywords:	PRIMARY CARE, Gastrointestinal tumours < GASTROENTEROLOGY, Gastrointestinal tumours < ONCOLOGY, PREVENTIVE MEDICINE, PUBLIC HEALTH

SCHOLARONE™ Manuscripts

I, the Submitting Author has the right to grant and does grant on behalf of all authors of the Work (as defined in the below author licence), an exclusive licence and/or a non-exclusive licence for contributions from authors who are: i) UK Crown employees; ii) where BMJ has agreed a CC-BY licence shall apply, and/or iii) in accordance with the terms applicable for US Federal Government officers or employees acting as part of their official duties; on a worldwide, perpetual, irrevocable, royalty-free basis to BMJ Publishing Group Ltd ("BMJ") its licensees and where the relevant Journal is co-owned by BMJ to the co-owners of the Journal, to publish the Work in this journal and any other BMJ products and to exploit all rights, as set out in our licence.

The Submitting Author accepts and understands that any supply made under these terms is made by BMJ to the Submitting Author unless you are acting as an employee on behalf of your employer or a postgraduate student of an affiliated institution which is paying any applicable article publishing charge ("APC") for Open Access articles. Where the Submitting Author wishes to make the Work available on an Open Access basis (and intends to pay the relevant APC), the terms of reuse of such Open Access shall be governed by a Creative Commons licence – details of these licences and which Creative Commons licence will apply to this Work are set out in our licence referred to above.

Other than as permitted in any relevant BMJ Author's Self Archiving Policies, I confirm this Work has not been accepted for publication elsewhere, is not being considered for publication elsewhere and does not duplicate material already published. I confirm all authors consent to publication of this Work and authorise the granting of this licence.

For peer review only

Patient-reported experiences and views on the Cytosponge test: a mixed-methods analysis from the BEST3 trial

Roberta Maroni[1*], Jessica Barnes[2*], Judith Offman[2*], Fiona Scheibl[3], Samuel G Smith[4], Irene Debiram-Beecham[5], Jo Waller[2], Peter D Sasieni[2], Rebecca C Fitzgerald[5,6], Greg Rubin[7], BEST3 Consortium[6], Fiona M Walter[9,10]

[1] Cancer Research UK and King's College London Cancer Prevention Trials Unit, Cancer Prevention Group, School of Cancer and Pharmaceutical Sciences, King's College London, London, UK

[2] Cancer Prevention Group, School of Cancer and Pharmaceutical Sciences, King's College London, London, UK

[3] Norwich Medical School, University of East Anglia, Norwich, UK

[4] Leeds Institute of Health Sciences, University of Leeds, Leeds, UK

[5] MRC Cancer Unit, Hutchison-MRC Research Centre, University of Cambridge, Cambridge, UK

[6] Cambridge University Hospitals NHS Foundation Trust, Cambridge, UK

[7] Population Health Sciences Institute, Newcastle University, 5th Floor, Ridley 1, Newcastle upon Tyne, UK

[8] BEST3 Trial team NIHR, Clinical Research Networks, UK

[9] The Primary Care Unit, Department of Public Health & Primary Care, University of Cambridge, UK

[10] Institute of Population Health Sciences, Queen Mary University of London, UK

*Roberta Maroni, Jessica Barnes and Judith Offman equally contributed to this paper.

Corresponding author:

Judith Offman, King's College London, School of Cancer & Pharmaceutical Sciences, Innovation Hub, Guys Cancer Centre, Guys Hospital, Great Maze Pond, London SE1 9RT, United Kingdom, Judith.offman@kcl.ac.uk

WORD COUNT (excluding title page, abstract, references, figures and tables):

4,914

For peer review only - http://bmjopen.bmj.com/site/about/guidelines.xhtml

ABSTRACT

Objectives: The BEST3 trial demonstrated the efficacy and safety of the Cytosponge-TFF3, a cell collection device coupled with the biomarker trefoil factor 3, as a tool for detecting Barrett's oesophagus, a precursor of oesophageal adenocarcinoma (OAC), in primary care. In this nested study, our aim was to understand patient experiences.

Design: Mixed methods using questionnaires (including Inventory to Assess Patient Satisfaction, STAI-6 and two-item perceived risk) and interviews.

Outcome measures: Participant satisfaction, anxiety and perceived risk of developing OAC.

Setting: General practices in England.

Participants: Patients with acid reflux enrolled in the intervention arm of the BEST3 trial and attending the Cytosponge appointment (N = 1750).

Results: 1488 patients successfully swallowing the Cytosponge completed the follow-up questionnaires, while 30 were interviewed, including some with an unsuccessful swallow.

Overall, participants were satisfied with the Cytosponge test. Several items showed positive ratings, in particular convenience and accessibility, staff's interpersonal skills and perceived technical competence. The most discomfort was reported during the Cytosponge removal, with more than 60% of participants experiencing gagging. Nevertheless, about 80% were willing to have the procedure again or to recommend it to friends; this was true even for participants experiencing discomfort, as confirmed in the interviews.

Median anxiety scores were below the pre-defined level of clinically significant anxiety and slightly decreased between baseline and follow-up ($p < 0.001$). Interviews revealed concerns around the ability to swallow, participating in a clinical trial, and waiting for test results.

The perceived risk of OAC increased following the Cytosponge appointment ($p < 0.001$). Moreover, interviews suggested that some participants had trouble conceptualising risk and did not understand the relationships between test results, gastro-oesophageal reflux, and risk of Barrett's oesophagus and OAC.

Conclusions: When delivered during a trial in primary care, the Cytosponge is well accepted and causes little anxiety.

Trial registration: ISRCTN68382401

Keywords: Barrett's oesophagus, oesophageal cancer, Cytosponge, anxiety, perceived risk, patient satisfaction, acceptability, mixed methods, questionnaires, interviews, primary care.

ARTICLE SUMMARY

Strengths and limitations of this study

- Our study is the first to explore patients' experiences of, and satisfaction with, the Cytosponge test in the primary care setting, gaining in-depth understanding by using questionnaire and interview data in a mixed-methods approach.

- Thirty participants, purposively sampled to reflect a range of characteristics (gender, age group, geographic region, Cytosponge result), underwent semi-structured interviews, whose analyses were underpinned by a robust approach, including a conceptual framework.

- A small proportion (10%) of patients undergoing the Cytosponge test did not complete a follow-up questionnaire.

For peer review only - http://bmjopen.bmj.com/site/about/guidelines.xhtml

- The Inventory to Assess Patient Satisfaction in the follow-up questionnaire was adapted from flexible sigmoidoscopy and validated on a small number of individuals.

INTRODUCTION

In the UK, oesophageal adenocarcinoma (OAC) is the seventh most common cause of cancer death. It has a bleak prognosis, with a 5-year net survival of just 17%.[1] Most cases of OAC are preceded by Barrett's oesophagus (BO), which provides an opportunity for early detection.[2, 3]BO Besides age, sex (male), obesity, ethnicity (Caucasian) and family history,[4, 5] the most important risk factor for BO is gastro-oesophageal reflux disease (GORD). Currently, only around 20% of patients with BO are diagnosed [6] since endoscopy is not feasible for all patients with GORD, and not all patients with GORD experience heartburn symptoms and so they may not come to medical attention. Overall, GORD burdens about 20% of the adult population [7] and is usually managed effectively with acid-suppressant medications and endoscopy referrals, which are suggested by NICE only if the symptoms are not controlled.[8] However, endoscopies are invasive, expensive [9] and entail some risks.[10] Given that pressures on endoscopy capacity in secondary care in the UK have been exacerbated by the recent COVID-19 pandemic,[11] novel technologies to help detect BO are now more critical than ever.

The Cytosponge is a cell collection device, which, coupled with the biomarker trefoil factor 3 (TFF3), can be used to identify BO. The device consists of a sponge tied to a string and compressed into a gelatine capsule, which is swallowed by the patient and retrieved by pulling on the string once the capsule has dissolved. The cell sample is then processed in a laboratory for immunohistochemical staining with TFF3. The Cytosponge-TFF3 test has been evaluated among more than 2000 patients in two clinical settings,[12, 13] proving its safety, cost-effectiveness, and accuracy as a potential test for BO.[14-17] The large (N > 13,000), pragmatic, randomised, controlled BEST3 trial was recently conducted in primary care in England [18] and demonstrated that offer of the Cytosponge test to individuals on medication for recurrent reflux symptoms identified ten times more cases of BO than usual care. In this trial, fewer than 10% of participants successfully swallowing the Cytosponge reported any side effects, and those were mainly mild (e.g. sore throat).

Successful implementation of a new diagnostic device requires not only evidence on diagnostic accuracy, safety, effectiveness and cost-effectiveness, but also an understanding of patient experience and satisfaction, including the identification of possible barriers to uptake. During the BEST3 trial patients were invited to receive the Cytosponge by postal letter. Invitation uptake was 24% (1654/6834) and median overall acceptability on an 11-point visual analogue scale from 'completely unacceptable' to 'completely acceptable' was 9 (interquartile range (IQR) 8-10).[18] Previous BEST studies [12, 13, 16, 17] reported results on acceptability and one of the studies [17] on anxiety scores. However, this nested mixed methods study as part of the BEST3 trial investigated patients' experiences of the Cytosponge in primary care, any anxiety caused by the test, and perceived risk of OAC were investigated more extensively by means of questionnaires and individual interviews.

METHODS

Study design

The design of the BEST3 trial is described in more detail in the Supplementary Materials and elsewhere.[18, 19] It enrolled participants aged 50 or over with GORD symptoms, identified via their general practice prescribing records. For this nested study, only participants in the intervention arm of the trial attending a Cytosponge appointment were included (N = 1750). Participants with an 'inadequate' test result (i.e. low-confidence negative TFF3, equivocal, or processing/technical failure) were invited to a repeat appointment when possible. All patients with a positive TFF3 result were referred for an endoscopy to establish a diagnosis.

For peer review only - http://bmjopen.bmj.com/site/about/guidelines.xhtml

Patient and public involvement

Patient and public involvement representatives were involved in all stages of the BEST3 trial, including two as members of the BEST3 trial steering committee;[19] they reviewed the protocol and the interview topics, and contributed to the early analysis of the patient interviews. The adapted Inventory to Assess Patient Satisfaction (IAPS) was piloted on eight individuals who had previously had the Cytosponge procedure to check for comprehension.

Data collection

a. Quantitative data

At the Cytosponge appointment, in each participant's general practice, a nurse collected demographic, anthropometric and clinical data, including the GORD Impact Scale.[20] This is a nine-item assessment of GORD symptoms experienced in the week before the appointment, which was duplicated to also address any symptoms from before patients started taking acid-suppressant medications.

Immediately before having the test, participants were asked to complete the baseline questionnaire, with questions on:

- Education level, smoking/alcohol history, family history of heartburn/BO/cancer;
- A shorter six-item form of the Spielberger State-Trait Anxiety Inventory (STAI-6);[21]
- Perceived risk of OAC, using two items widely applied for other cancer risk assessments: perceived risk compared with a person of the same age (comparative risk) and per cent absolute risk of developing OAC in their lifetime.[22]

A week to 14 days later, participants who successfully swallowed the Cytosponge (1654/1750, 95%) were invited to fill in a follow-up questionnaire. A reminder to complete the questionnaire was sent after two weeks, and some received a further reminder. The follow-up questionnaire consisted of:

- The IAPS, with 22 items addressing both positive and negative aspects of the experience, adapted from a study on flexible screening sigmoidoscopy [23];
- STAI-6;
- Perceived risk of OAC (two items).

b. Qualitative data

Participants were purposively sampled to reflect a range of characteristics: gender, age group (50-59, 60-69, 70-79, 80+), geographic region in England (East, North-East, West) and Cytosponge result at first appointment (positive, negative, low confidence/equivocal, unsuccessful swallow), see Table 1 and Supplementary Table 1. Semi-structured interviews were conducted face-to-face at home or in clinics in the only presence of a female qualitative researcher (FS), with the aim of interviewing 30 participants within six weeks of their Cytosponge test (to reduce issues with recall). Interviews lasted 23 minutes on average (range: 13-50 minutes), were audio-recorded and transcribed verbatim for analysis. More details on qualitative data collection are in the Supplementary Materials.

Analysis

a. Quantitative analysis

Questionnaire scoring is described in the Supplementary Materials. Patient characteristics and GORD Impact Scale responses were analysed according to three subgroups of participation: attended the Cytosponge test appointment and completed the baseline questionnaire *('attenders'); 'attenders'* who completed the follow-up questionnaire *('follow-up responders')*; *'attenders'* who undertook an interview *('interviewees')*, see Supplementary Figure 1.

For peer review only - http://bmjopen.bmj.com/site/about/guidelines.xhtml

STAI-6 and perceived risk of OAC are presented only for the subgroup of participants completing at least one of those items in both baseline and follow-up questionnaires. STAI-6 scores between the two time points were compared using Wilcoxon matched-pairs signed-rank test, while differences in risk perceptions were analysed by McNemar's test, which included only patients with scores different than the neutral (e.g. 'Neither higher or lower') or middle-ranking category (from a list of ordered options). A STAI-6 score over 40 was predefined as a threshold for clinically significant anxiety.[12, 24]

Statistical significance was based on a two-tailed test with size of 5%. Analyses were performed using Stata version 15.[25]

b. Qualitative analysis

We undertook a thematic analysis, having organised and managed data according to the Framework approach.[26] For more details, see Supplementary Materials. Briefly, this involved identifying an initial, broad set of labels inductively and deductively that would be used to categorise and sort the data to enable the subsequent thematic analysis. Inductively-created labels were based on emergent concepts identified in the data. Deductively-created labels were based on the IAPS [23], which allowed us to more directly relate participant experience across qualitative and quantitative datasets. Use of the Theoretical Framework of Acceptability [27] constructs allowed us to examine additional dimensions of patient experience associated with acceptability that were not captured by the IAPS. We then conducted the thematic analysis, aiming to achieve both description and explanation with the dataset. Data within each column of the Framework matrix was explored and further organised into more abstract themes, using drawings.net open-source software to allow visual representation and coding of the data, therefore facilitating the identification patterns and linkages between different types of participant experience and/or demographic characteristics. Participants did not provide feedback on the findings.

RESULTS

Demographics and baseline characteristics

A trial flowchart from the intervention arm of the BEST3 trial is shown in Supplementary Figure 1. There were 1750 participants who completed the baseline questionnaire at Cytosponge appointment *('attenders')*, with a minimum completion rate of 80% (12/15), considering only questions applicable to all participants. The follow-up questionnaire was completed by 1488 participants (90% of 1654 successful swallows) *('follow-up responders')*, with a minimum completion rate of 23% (7/31) at a median of 10 days (IQR 7-14 days) after undergoing the Cytosponge test. 159 participants (11% of 1488) completed the follow-up questionnaire after being mailed the letter with their Cytosponge test results, 5 (0.3%) after attending a repeat Cytosponge test and one after receiving their repeat Cytosponge test result.

Out of the 1750 *'attenders'*, 75 (4%) were invited for an interview; 30 interviews were completed *('interviewees')* at a median of 59 days (IQR 48-78 days) after the Cytosponge test. At the time of interview, all participants who successfully swallowed the Cytosponge had received their first test result, and one was still waiting for the result from their repeat test, while another had declined to have a repeat test. Among participants who had received a positive test result, some were awaiting confirmatory endoscopy, while others had already had theirs.

Table 1 shows patient and clinical characteristics for the three subgroups of participants. Those completing the follow-up questionnaire differed from the non-completers (N = 262) by age group, waist-hip ratio categories and comorbidity status.

1. Patient-reported satisfaction and experiences of the Cytosponge test

Participants were generally satisfied with their experiences of the processes undertaken before, during and immediately after the Cytosponge test (Figure 1 and Table 2). Several items of the IAPS were rated positively by

For peer review only - http://bmjopen.bmj.com/site/about/guidelines.xhtml

most participants (Table 3). The Cronbach's α, measuring the IAPS reliability, was 0.83 overall, and it ranged between 0.81 and 0.83 (improving the overall coefficient in three instances) when excluding each of the 22 items at a time.

1.1 Convenience and accessibility

The majority of participants (92-94%) rated the study sites' convenience and accessibility positively. In the interviews, some commented that it was practical to go to their own General Practice and that this was preferable to going to a hospital appointment. Participants also appreciated the scheduling, as they were able to select from a range of appointment dates and times that suited them.

1.2 Staff interpersonal skills

Staff interpersonal skills were rated positively by 96%-98% of participants, and uniformly described across the interviews in very good terms. Participants felt that they had adequate opportunities to ask questions, which the nurses were able to answer well providing important reassurance. The interpersonal manner of staff was consistently described in highly positive ways by participants – staff were "calm", "in control", "friendly", "helpful", "supportive", "professional", and created an experience that was "straightforward and bordering on enjoyable". When participants failed to swallow the Cytosponge, staff were empathetic and reassuring.

1.3 Perceived technical competence of staff

The majority of participants (93%-96%) agreed that the staff was competent. In the interviews, participants focused on the speed and efficiency of the Cytosponge removal – for example, that the staff members had good technique, and they went quickly enough to get removal done efficiently but slowly enough to gather cells. Some issues related to technical skills were noted: one participant described how their procedure was performed by a practice nurse, and the Cytosponge seemed to get stuck partway through removal and caused pain. In this case, the practice nurse needed to consult a research nurse who advised how to resolve the issue.

1.4 Swallowing the Cytosponge capsule

The majority of participants (69%-87%) rated swallowing the Cytosponge positively. Among the lowest rated measure of satisfaction was "*I had to gag when I swallowed the Cytosponge capsule*" (N = 373, 25% agreed).

When interview participants described the procedure as straightforward, they recounted the swallowing aspect as routine and nothing unexpected.

> *"I think that's just normal as taking a tablet, the only difference is it's got string on it."*
> *[age 60-69, negative result]*

When participants described the swallowing as involving minimal difficulty, they commented on characteristics such as the string being uncomfortable, or that it was difficult to drink enough water to get the string and capsule down. However, these difficulties were mainly perceived as nothing to worry about.

Interviewees who reported significant difficulty swallowing, such as gagging, retching or heaving, underlined issues with being unable to place the capsule and string far enough at the back of their throat without causing themselves to gag; in some cases, this could be rectified by the nurse placing the string and capsule instead of the participant. Participants who failed to swallow reported struggling with getting the string down as it unwound, and gagging too much to be able to drink water to wash the string and capsule down the oesophagus. One participant reported that they had not realised that they would be required to swallow the string in a bundle, and if they had known this, they may have declined to participate. During the interviews, responses were varied amongst the four participants who failed to swallow: some would not do the procedure again but would still recommend it to friends, while others would still try the procedure again in the future.

> *"Well swallowing the capsule was all right. The string attached to it was a bit difficult, it felt a bit like a cat trying to swallow a mouse, you know, can't get the tail in the mouth. [...] It went down all right… it was just an odd feeling with the string coming up."*
> *[age 60-69, negative result]*

For peer review only - http://bmjopen.bmj.com/site/about/guidelines.xhtml

1.5 Waiting with Cytosponge in stomach

Overall, 85-92% participants rated the experience of waiting for the capsule to dissolve in their stomach positively. During the interviews, some reported not being able to feel anything untoward at all, nor did they experience any distress. Others reported minor issues, such as being aware of the string, tickling or gagging when trying to talk, but these experiences were not considered concerning.

> *"The only strange sensation was... after I'd swallowed the pill, it was having a tiny piece of cotton or whatever it was hanging out, but the way [the nurses] talked, it took my mind off it anyway."*
> *[age 60-69, negative result]*

Some participants discussed more distressing experiences: one experienced "pains in my stomach", significant enough for them to ask the nurse to remove the Cytosponge prematurely. This procedure resulted in a low confidence/equivocal result, and the participant attended a repeat appointment, where they were able to successfully swallow the Cytosponge and it was "less uncomfortable" while the capsule was dissolving. The participant suggested that this may be because they drank more water the second time, causing the Cytosponge to successfully reach the stomach.

1.6 Retrieving the Cytosponge

Amongst the lowest rated measures of satisfaction were: "*I had to gag when the Cytosponge was pulled up*" (N = 889, 60% agreed) and "*Pulling up of the Cytosponge was more comfortable than I expected*" (N = 354, 24% disagreed).

During the interviews, participants gave more detailed descriptions of this part of the Cytosponge test, with some reporting a number of types of discomfort during removal (Figure 1 and Supplementary Box 1). Not all of them were particularly serious or concerning. Despite these experiences of discomfort, participants often expressed a willingness to have the Cytosponge test again and to recommend it to others, as confirmed by responses to the IAPS questionnaire (61% and 65% of participants with low average satisfaction scores for items about pulling the Cytosponge, respectively). One interviewee was unwilling to have the procedure again due to the perceived possibility of the string breaking and an endoscopy being necessary to retrieve it.

1.7 Expectations, beliefs and general satisfaction

A fifth of the participants (20%) agreed with the item *"I was very anxious about having the Cytosponge test"*, while 97% reported being very satisfied with the care received.

During the interviews, participants discussed a range of after-effects (including none). Some explained that they felt fine after their appointment, and sometimes forgot completely about it until they received their results letter. Some participants described experiencing a sore, scratchy or tickly throat that resolved relatively quickly. Some reported experiencing unexpected reflux following their test.

Experiences linked to the understanding of the test results, discussed during the patient interviews, are presented in Supplementary Table 2. Upon receiving their Cytosponge-TFF3 test results, participants reacted in ways that were influenced by their expectations, which varied due to a number of interplaying factors. These included: their understanding of the purpose of the test; previous relevant experiences that had improved their literacy of such test results; and their conceptualisation of the causes of cancer in general.

Some participants receiving a positive test result reacted with shock as the result went against their expectations, which were based on their understanding of the causes of cancer in general: their explanations of their reaction to their test result revealed an assumption that a positive result should only be expected by people who have a particular lifestyle or risk factors (such as a history of drinking or smoking), or if BO or oesophageal cancer runs in the family. Other participants who reacted with shock to a positive test result described that the trial's reference to cancer was heightened in their mind, so receiving a positive result was experienced to some degree as being like receiving a cancer diagnosis.

For peer review only - http://bmjopen.bmj.com/site/about/guidelines.xhtml

There was an issue with the language that was used to report test results, which caused confusion and concern. For positive test results, the issue was around use of the term "positive" as some participants initially interpreted this in the lay sense as meaning "good". Alternative terminology such as "needs further investigation" was recommended. Another issue with phrasing was around the reassurances that a positive result was "nothing to be unduly concerned about". Participants explained that, paradoxically, this made them more concerned, and they felt that they were not given adequate information about what a positive result meant to enable them to understand why exactly they should not be concerned.

2. Patient-reported anxiety before and after the Cytosponge test

Participants who completed both pre- and post-test measures (N = 1418) had a median STAI-6 score of 33 (IQR 23-40; possible range: 20-80) at baseline and 27 (IQR 20-37) at follow-up. As a comparison, the median score for participants not filling in the follow-up questionnaire was 33 at baseline (IQR 23-43). A score of over 40 was predefined as meeting a clinical threshold of anxiety: 334 (24%) and 166 (12%) reported such scores at baseline and follow-up, respectively. There was a statistically significant difference in scores between baseline and follow-up ($p < 0.001$), with a median change between follow-up and baseline of -3 (IQR -10-0). For a breakdown of scores by questionnaire, see Supplementary Table 3.

Interviewees offered reflections on how they were feeling the day of the appointment or the night before. Some were worried about being able to complete the test (e.g. participants who had problems swallowing) or the test itself. A few were concerned about 'the unknown' and it being 'experimental'. Reflecting on the period after the Cytosponge test, some described how receiving their result alleviated the sense of anxiety and uncertainty that they had been experiencing. Other participants, however, reported not being particularly bothered while waiting for their results.

3. Perceived risk of oesophageal cancer

Amongst participants filling in both questionnaires, just over half (N = 739, 50% at baseline; N = 861, 58% at follow-up) considered their risk to be equivalent to that of someone of the same age (see Figure 2a for a comparison of the ratings between baseline and follow-on questionnaire). Opinions on absolute risk in a lifetime were more varied: while the largest group pre-test (N = 712, 48%) thought that their risk was not more than 5%, the largest group post-test (N = 657, 44%) expected theirs to be higher than 25% (see Figure 2b for a comparison of the ratings pre-test and post-test). There was a statistically significant change ($p < 0.001$) for both items of perceived risk between baseline and follow-up, with 319 (21%) and 389 (26%) participants thinking that their chances of OAC had increased for comparative and absolute risk, respectively (Supplementary Tables 4 and 5).

Some interview participants did not demonstrate a good understanding of the relationship between reflux, BO and OAC, which may have led them to different interpretations of questions about personal risk of BO and OAC, and the size of their risk:

> *"…when I was searching for the probability, the ratio of Barrett's to actual oesophageal cancer, I seemed to be getting different answers."*
> *[age 70-79, positive test result]*

Some participants found the information about risk in the invitation leaflet difficult to understand but drew attention to the important role that the nurses played in explaining this to them at the start of the appointment.

> *"Because I'd never heard of Barrett's before,... obviously it was on the leaflets I read that, but when I actually come for the test the nurse that I saw... she explained it all to me and… how that can be a sign that you may get the cancerous cells and things like that. So yeah, it was very interesting. I didn't know that."*
> *[age 60-69, inadequate test result]*

For peer review only - http://bmjopen.bmj.com/site/about/guidelines.xhtml

DISCUSSION

Summary of main findings

This mixed-methods study evaluated patients' experiences of, and satisfaction with, the Cytosponge test in primary care as part of the BEST3 trial. Overall, participants were satisfied with their experience of the Cytosponge: they found it very convenient to attend their appointment at their own general practice and rated the staff interpersonal skills and competence very highly. Regarding the Cytosponge procedure itself, 87% of participants did not find swallowing very uncomfortable, while 60% reported gagging during the Cytosponge withdrawal in the questionnaire data; despite that, more than 80% were willing to have the test again or to recommend it to others. In interviews, patients provided more detailed descriptions of their experience, specifically different levels of pain and scratching resulting in a sore throat. Questionnaire data found a slight decrease in anxiety levels between before and after the test, and interviews helped identify patients' underlying motivations for feeling anxious: their ability to swallow, participating in a clinical trial, and waiting for test results. Lastly, we observed a statistically significant change in perceived risk of OAC pre- and post-test with 21% to 26% (depending on the risk type) of participants rating their risk as higher at follow-up. Interview data suggested that information about risk in the invitation leaflet was difficult to interpret for some patients and that nurses played an important role in providing more information on risk at the appointment, despite participants still not having a good understanding of the relationship between reflux, BO and OAC.

Interpretation

This study has provided a deeper understanding of those aspects of the Cytosponge test that worked well in the trial and would need to be maintained to ensure acceptability during implementation. First, delivering the Cytosponge near home was perceived as convenient and acceptable. Second, the nurses administering the Cytosponge were rated as supportive, knowledgeable and reassuring. Third, staff technical competence was also rated very highly. Implementation of the Cytosponge test as a routine diagnostic test in primary care will need to ensure balance between convenience and adequate staff training.

Some aspects of the Cytosponge test were rated less well and our interview data provide insights into what could be changed. First, although the majority of participants (95%) were able to successfully swallow the device, swallowing and retrieval of the Cytosponge were less highly rated. Despite experiencing different levels of discomfort, most participants found that the pain was as expected, suggesting that it is important to explain how the Cytosponge is removed, that removal is brief, and that some discomfort may be necessary for the sponge to effectively gather cells. To ensure a good overall experience continues when implementing the Cytosponge in primary care, it will be important to provide high-quality information and manage patient expectations of the physical experience, as was done in BEST3. This was achieved by explaining the procedure using the BEST3 leaflet and a demo Cytosponge as support, and reassuring about the potential risks at the beginning of the appointment. Second, some interviewees reported varying levels of pre-test anxiety linked to concerns about swallowing the device and general fear of the unknown. However, the median STAI-6 scores observed before the procedure and at follow-up were both well under the predefined level of clinically significant anxiety of 40 in the average adult population in a non-clinical setting.[24] In some cases, pre-test anxiety might improve once patients are more familiar with the Cytosponge. While these findings are broadly reassuring, efforts should be made to ensure patients know what to expect and are supported if they feel anxious. Using the same leaflet as in BEST3 and a demo Cytosponge, this should be achievable within the timeframe available in standard clinical NHS practice.

At both time points, the majority of participants rated their risk of OAC as being average for people of their age, showing some evidence of the 'optimistic bias' often observed in measures of comparative risk. At follow-up, a greater proportion of people rated their risk as being above average, which may reflect a greater awareness of the association between reflux, BO and OAC following the procedure. However, the qualitative data point to an inconsistent understanding of the relationships between these three conditions. There are

For peer review only - http://bmjopen.bmj.com/site/about/guidelines.xhtml

some parallels with the cervical screening context: confusion about the relationship between human papillomavirus (HPV), cervical intraepithelial neoplasia and cervical cancer is common and many women receiving a positive HPV result report adverse psychological outcomes.[28] As Cytosponge testing is rolled out more widely, it will be important to use best practice in risk communication [29] to ensure people understand the meaning of results to minimise misunderstanding and poor psychological outcomes.

Context of other literature

Previously, acceptability of the Cytosponge test had been assessed using a visual analogue scale ranging from 0 (worst) to 10 (best experience).[12, 13, 30] A review of five studies assessing the Cytosponge test found a satisfactory overall acceptability, with a median score of 6.[16] In addition, the BEST1 study showed, using the STAI-6, that anxiety levels were low before and after the test with similar scores obtained as in this current study.[12] One qualitative study has investigated the acceptability of Cytosponge, but the participants had not actually taken the test, so their attitudes were hypothetical.[31] It showed that acceptability was high despite initial concerns about swallowing and extracting the capsule.

Even though BEST3 participants experienced different levels of discomfort or pain during the swallowing and removal stages of the procedure, in most cases this would not discourage them from having the test again or recommending it to someone else. This is relevant in the context of implementing the Cytosponge as a routine test. Interestingly, studies investigating barriers to screening attendance found varying degrees of association between pain and re-attendance, with 25-46% of women citing pain of having a mammography as a reason for non-attendance;[32] however, worry about pain was not associated with low intention to re-attend cervical screening.[33]

Strengths and limitations

This study was undertaken within a large pragmatic randomised controlled trial, in which 1750 patients attended the Cytosponge appointment. Key strengths are that the BEST3 trial was set in primary care, where Cytosponge implementation is planned, and that this study used a mixed-methods approach. The findings from the IAPS, STAI-6 and perceived risk questionnaires, completed by nearly 1500 participants, were explored in more depth during interviews with a diverse sample of 30 patients, which included patients with unsuccessful swallows whose experience had otherwise not been captured in the follow-up questionnaire. The qualitative analyses, supported by a conceptual framework, offered detailed insights of participants' experiences and enriched the interpretation of the quantitative findings.

This study had limitations. Some attendees (N = 262, 15%) did not return the follow-up questionnaire, and there were some small statistically significant differences in the distribution of patients' characteristics in those completing vs not completing the follow-up questionnaire. However, a simulation including the non-completers and assuming that they had given the worst ratings to their Cytosponge experience in the IAPS and STAI-6 questionnaires showed good overall levels of patient satisfaction (about 80%) and relatively low levels of anxiety (median 30, IQR 20-43, results not shown).

The IAPS, which had been adapted from flexible sigmoidoscopy, was only validated by piloting with a small number of patients, but the Cronbach's alpha of 0.83 indicates appropriate internal reliability of the adaptation of the questionnaire to the Cytosponge test. The predefined threshold of clinical anxiety (over 40) used in our analysis for the STAI-6 was defined in the literature for a non-clinical setting and for the complete STAI questionnaire.[24] The main limitation of the qualitative findings was that, for some, more than six weeks elapsed from a participant's Cytosponge procedure and their interview. This may have affected recall, although most participants were able to remember their experiences in substantial detail.

Conclusion

This study, exploring patients' experiences of, and satisfaction with, the Cytosponge test used extensive questionnaire and in-depth interview data. Overall, participants were satisfied with their experiences and we did not observe excess anxiety due to the procedure. Identifying aspects of the procedure which are currently working well or rated less positively will enable specific improvements to communications with patients, for

For peer review only - http://bmjopen.bmj.com/site/about/guidelines.xhtml

example on how to better communicate test results, that will result in a better experience once the Cytosponge test is implemented in clinical care.

For peer review only

For peer review only - http://bmjopen.bmj.com/site/about/guidelines.xhtml

TABLES

Table 1. Patient characteristics and GORD Impact Scale for the three subgroups of participation: attended the Cytosponge appointment and completed the baseline questionnaire *('attenders')*; completed the follow-up questionnaire *('follow-up responders')*; or interviewed *('interviewees')*.

	**Completed baseline questionnaire *('attenders')* (N = 1750)**		**Completed follow-up questionnaire *('follow-up responders')* (N = 1488)**		**Interviewed *('interviewees')* (N = 30)**		**p-values for chi-squared test between *'follow-up responders'* (N = 1488) and *'attenders'* who are not *'follow-up responders'* (N = 262)**
	**N**	**%**	**N**	**%**	**N**	**%**	
**Sex**							
Female	919	53%	782	52%	15	50%	0.985
Male	830	47%	706	47%	15	50%	
Missing	1	<1%	0	0%	0	0%	
**Age group**							
50-59	345	20%	285	19%	4	13%	*0.028
60-69	596	34%	497	33%	11	35%	
70-79	647	37%	572	38%	9	29%	
80-99	161	9%	134	9%	6	19%	
Missing	1	<1%	0	0%	1	3%	
**Cytosponge-TFF3 outcome (after repeat test)**							
TFF3 negative	1252	72%	1126	76%	14	47%	^0.246
TFF3 positive	231	13%	213	14%	10	33%	
Inadequate (equivocal/low-confidence negative/technical or processing failure)	171	10%	149	10%	2	7%	
Unsuccessful swallow	96	5%	0	0%	4	13%	
**Underwent repeat Cytosponge test**							
No	1560	89%	1322	89%	25	83%	0.338
Yes	190	11%	166	11%	5	17%	
**Education level**							
School up to 15-16 years of age	712	41%	605	41%	16	53%	0.104
College or vocational school	537	31%	455	31%	8	27%	
Professional training beyond college, university graduate or postgraduate degree	480	27%	414	28%	4	13%	
Other or prefer not to say	21	1%	14	1%	2	7%	
**Waist-hip ratio**							
<0.90	685	39%	601	40%	11	37%	*0.010
0.90<0.99	686	39%	562	38%	10	33%	
0.99+	378	22%	324	22%	9	30%	
Missing	1	<1%	1	<1%	0	0%	
**Comorbidities**							
No	228	13%	182	12%	6	20%	*0.018
Yes	1522	87%	1306	88%	24	80%	
**Medication duration**							
Less than 5 years	518	30%	431	29%	7	23%	0.166
More than 5 years	1232	70%	1057	71%	23	77%	
**Diagnoses**							

For peer review only

For peer review only - http://bmjopen.bmj.com/site/about/guidelines.xhtml

No Barrett's oesophagus	1618	92%	1367	92%	26	87%	0.118
Barrett's oesophagus – without dysplasia	117	7%	106	7%	4	13%	
Barrett's oesophagus – with dysplasia	11	1%	11	1%	0	0%	
Oesophageal adenocarcinoma (stage 1)	4	<1%	4	<1%	0	0%	
							**p-values for t-test between *'follow-up responders'* (N = 1488) and *'attenders'* who are not *'follow-up responders'* (N = 262)**
**GORD Impact Scale – Before taking acid-suppressant medications**							
Mean (SD)		1.9 (0.5)		1.9 (0.5)		1.9 (0.5)	0.319
No. missing		2		1		0	
**GORD Impact Scale – In the last week**							
Mean (SD)		1.3 (0.4)		1.3 (0.4)		1.3 (0.5)	0.451
No. missing		0		0		0	

* $p < 0.05$
^ Comparison excluding participants producing an unsuccessful swallow as they were not invited to fill in a follow-up questionnaire.

TFF3 = trefoil factor 3, GORD = gastro-oesophageal reflux disease, SD = standard deviation

For peer review only

Table 2: Findings from questionnaires and patient interviews: Example interview quotes illustrating the practical elements of the Cytosponge procedure

Aspect of Cytosponge procedure	Example interview quotes
Convenience and accessibility	*Convenient alternative to a procedure in secondary care:*<br>"[…] from what [the nurse] was saying to me is that [the Cytosponge procedure] takes away that waiting for a hospital appointment, that you can have it done in the [GP] surgery, and if it was me again and, I don't know, something was not quite right, I wouldn't hesitate at coming down and having that done. Not at all, not at all."<br>*(age 60-69, inadequate test result at first appointment)*
Staff interpersonal skills	*Positive interpersonal skills:*<br>"The [nurse] who actually did it was really lovely. She really was. She was very calm, very in control and we chatted about different things and she was about to get married and all this sort of thing and it was, we learned a bit about each other, which was absolutely fine […] [The procedure is] done very nicely, lovely people, nothing to worry about, go and get it done."<br>*(age 70-79, negative test result)*<br>*Procedure explained clearly:*<br>"'I mean if I didn't understand then I asked to explain it. I think [the staff] were very helpful and very nice, the way they put things over. I mean there was the two of them here and what one didn't answer, the other one did. No, I think they were very helpful and very kind."<br>*(age 80 and over, failed swallow)*
Perceived technical competence of staff	*Staff were skilled at removing sponge:*<br>'No, it was fine, it was just that and she did really well, she [removed the Cytosponge] as quick as she could be, obviously she had to go slower to get what she needed."<br>*(age 50-59, negative test result)*<br>*Patients and inexperienced staff may need more guidance:*<br>"...the first part of the extraction [of the Cytosponge] was fairly non-event[ful] but then again it did get stuck a bit in my throat […] And the [practice] nurse had to ask the [research] nurse […] she just said pull harder. So she pulled harder and it popped out. […] So I don't know if positioning the throat in a different way or me being told to hold the throat in a particular angle may have helped but, I mean, I know that sword swallowers, they hold their throat quite straight […] But there was no advice as to how to hold your head or position your throat and I thought that might have been useful […] Well to hold the head in a particular position and relax may have helped, I don't know, it may have got stuck whatever."<br>*(age 70-79, positive test result)*
Swallowing of the Cytosponge	*Difficulties due to string and retching:*<br>"The first time, when I swallowed it, the string seemed to flick around in the back of my throat and it didn't go down properly, so I was trying to add a bit more water and that, but I couldn't […] I was just retching all the time and I couldn't even get […] the water in my mouth because I just kept retching all the time […] And then the second time, it went straight down, straight down. It was marvellous, it went straight down and I thought, oh, I've cracked it, so I just kept sipping, and then all of a sudden I think a bit of the string... Like I felt down at the side, and I just went uh, and it just came straight out, just all came straight out altogether. […] I think it's the water I drank, it was still lying on my stomach and just brought it straight back up."<br>*(age 50-59, failed swallow)*<br>*Swallowing was easy:*<br>"That swallowing the capsule was simple, there was no... it was easy, it was just a matter of a few mouthfuls of water and that was it."<br>*(age 50-59, positive test result)*
Waiting with Cytosponge in stomach	*Waiting was acceptable, especially when there were distractions:*

For peer review only - http://bmjopen.bmj.com/site/about/guidelines.xhtml

	"But it wasn't horrendous and for the time that I was there and, you know, and by the time I'd sort of swallowed [the Cytosponge] and answered a few questions, had a little chat and drank some water, it was time for it to come up."<br>*(age 60-69, negative test result)*<br>*Mild discomfort:*<br>"You're aware of the string being in the throat […] It was slightly uncomfortable […] It was making you want to [cough] […] [but] There was no problem with it."<br>*(age 60-60, negative test result)*
Pulling of the Cytosponge	*Experience of pain:*<br>"It was painful. It was worse than I was expecting […] the nurse explained it to me afterwards, because afterwards I said to her, I said wow, that was more painful than I was expecting, and she explained that where your muscles will work to push things downwards, obviously, she said, when you're pulling the sponge up you're going completely against everything that it's doing and, do you know, I couldn't even describe what sort of pain it was, but it was literally... well it felt like a sponge literally was pulling out, you know, but […] I have to say, it only lasted a few seconds, and once it was out I suppose I had a tickly throat for the rest of the day. Not hurting, just a bit scratchy, tickly, certainly no painkillers, nothing like that. It was just those few seconds of it actually coming out wasn't pleasant, no. […] I did come back again [for second appointment following inadequate test result]."<br>*(age 60-69, inadequate test result)*<br>*Discomfort from gagging/coughing:*<br>"It was all over in a matter of seconds, but it was when it hit the back of my throat, I did gag, and I started to cough or I had a coughing fit after it was out, I was red hot, you know, I think it was just with gagging, yeah, but it was fine, it was just something that lasted a matter of two seconds."<br>*(age 50-59, positive test result)*<br>*No discomfort:*<br>'It was over and done within a matter of… [...] Woosh, gone. [...] Finished, I didn't even feel it coming out."<br>*(age 60-69, negative test result)*

For peer review only - http://bmjopen.bmj.com/site/about/guidelines.xhtml

Table 3. Number and proportion of participants (N = 1488) by ratings for the 22 questions of the inventory to assess patient satisfaction.

	Disagree		Neither		Agree		Missing	
	N	%	N	%	N	%	N	%
**Convenience and accessibility**								
I did not feel that I had to wait too long.*	42	3%	24	2%	1395	94%	28	2%
The test is in a place that is easy for me to get to.	90	6%	4	<1%	1389	93%	5	<1%
I did not find it hard to find a convenient time to come to the test.*	71	5%	15	1%	1368	92%	34	2%
**Staff interpersonal skills**								
I felt free to ask the staff questions I wanted to ask.	23	2%	1	<1%	1456	98%	8	1%
The staff did not seem to hurry me through too quickly.*	9	1%	2	<1%	1454	98%	23	2%
The staff did not use words that were hard to understand.*	22	1%	10	1%	1425	96%	31	2%
**Perceived technical competence**								
The nurse or member of staff was not too rough when performing the Cytosponge test.*	20	1%	14	1%	1422	96%	32	2%
I feel confident that the Cytosponge test was performed properly.	86	6%	10	1%	1384	93%	8	1%
**Swallowing of the capsule**								
I did not have to gag when I swallowed the Cytosponge capsule.*	373	25%	53	4%	1020	69%	42	3%
Swallowing the Cytosponge capsule was more comfortable than I expected.	221	15%	169	11%	1073	72%	25	2%
Swallowing the Cytosponge capsule did not cause me great discomfort.*	82	6%	60	4%	1300	87%	46	3%
**Waiting with capsule in stomach**								
I did not have to gag while I waited with the Cytosponge capsule in my stomach.*	146	10%	36	2%	1264	85%	42	3%
Waiting with the Cytosponge capsule in my stomach was more comfortable than I expected.	123	8%	133	9%	1207	81%	25	2%
Waiting with the Cytosponge capsule in my stomach did not cause me great discomfort.*	39	3%	36	2%	1365	92%	48	3%
**Pulling of the Cytosponge**								
I did not have to gag when the Cytosponge was pulled up.*	889	60%	68	5%	491	33%	40	3%
Pulling up of the Cytosponge was more comfortable than I expected.	354	24%	234	16%	866	58%	34	2%
Pulling up of the Cytosponge did not cause me great discomfort.*	193	13%	108	7%	1134	76%	53	4%
**Expectations and beliefs**								
I was not very anxious about having the Cytosponge test.*	296	20%	132	9%	1029	69%	31	2%
Undergoing the Cytosponge test will benefit my health.	27	2%	281	19%	1153	77%	27	2%
**General satisfaction**								
I was very satisfied with the care I received.	16	1%	2	<1%	1450	97%	20	1%
I would recommend the Cytosponge test to my friends.	38	3%	184	12%	1236	83%	30	2%
I would be willing to have another test if necessary. As part of the Trial, you may still be contacted for a repeat Cytosponge test.	48	3%	229	15%	1185	80%	26	2%

* Items referring to negative aspects of patient experience were rephrased for this table using negative constructs to facilitate comparison between items. Changes are underlined.

For peer review only - http://bmjopen.bmj.com/site/about/guidelines.xhtml

AUTHOR CONTRIBUTIONS

RCF was the chief investigator of the BEST3 trial. GR and FMW conceptualised and JO, GR and FMW designed this mixed-methods study as nested within the BEST3 trial. FS conducted the patient interviews. ID-B, RM, JB and FS acquired and analysed the data. RM, JB, JO, SGS, JW, PDS and FMW interpreted the data. RM, JB, JO and FMW drafted the manuscript. PDS provided statistical support. All authors critically reviewed the manuscript and approved the final version.

FUNDING

The laboratory of RCF is funded by a Core Programme Grant from the Medical Research Council (RG84369). The BEST3 study was primarily funded by Cancer Research UK (C14478/A21047). The National Institute for Health Research (NIHR) covered service support costs and National Health Service commissioners funded excess treatment costs, while Medtronic funded Cytosponge devices and TFF3 antibodies. This research was supported by the NIHR Cambridge Biomedical Research Centre (BRC-1215-20014). Cancer Research UK provide funding to the Cancer Prevention Trials Unit (C8162/A25356) and Cancer Prevention Group at King's College London (C8162/A16892). SGS is supported by a Yorkshire Cancer Research Fellowship (L389SS). JW is funded by a Cancer Research UK career development fellowship (C7492/A17219). The funders had no role in study design, data collection, data analysis, data interpretation, or writing of the report.

ETHICAL APPROVAL

Ethical approval for the BEST3 trial was obtained from the East of England – Cambridge East Research Ethics Committee (Trial Registration ISRCTN68382401). All participants gave informed consent before any individual-level patient data were collected and any clinical procedure was done. In addition, interview participants provided written consent before participating in interviews.

ACKNOWLEDGMENTS

This research is linked to the CanTest Collaborative, which is funded by Cancer Research UK (C8640/A23385), of which FMW is Director and GR is Chair of the Steering Committee. We would also like to acknowledge patient input from Heartburn Cancer UK, and the contributions of our CanTest PPI lead, Mrs Margaret Johnson.

We acknowledge the contribution of statistician Irene Kaimi, who had a leading role early in the trial but who tragically died before the study was completed. We thank all of the sites and patients who participated in the BEST3 trial, without whom this research would not have been possible.

COMPETING INTERESTS

RCF is named on patents related to the Cytosponge-trefoil factor 3 test. Covidien GI Solutions (now Medtronic) licensed the Cytosponge from the Medical Research Council, and the device has now received the CE mark and is cleared by the US Food and Drug Administration. RCF is a shareholder in Cyted, a company working on early detection technology. PS reports fees paid to his organisation from GRAIL, outside of the submitted work. The remaining authors have no conflicts of interest to declare.

For peer review only - http://bmjopen.bmj.com/site/about/guidelines.xhtml

DATA SHARING STATEMENT

The trial protocol, statistical analysis plan, and statistical report will be available via the University of Cambridge data repository (https://www.data.cam.ac.uk/repository). Datasets will be available from the authors on request.

For peer review only

For peer review only - http://bmjopen.bmj.com/site/about/guidelines.xhtml

REFERENCES

1. Cancer Research UK. Oesophageal cancer statistics. Available from: https://www.cancerresearchuk.org/health-professional/cancer-statistics/statistics-by-cancer-type/oesophageal-cancer (Accessed 30th December 2020).
2. Cancer Research UK. Barrett's oesophagus. Available from: https://www.cancerresearchuk.org/about-cancer/other-conditions/barretts-oesophagus/about-barrett%27s (Accessed 30th December 2020).
3. Nowicki-Osuch K, Zhuang L, Jammula S, et al. Molecular phenotyping reveals the identity of Barrett’s esophagus and its malignant transition. *Science* 2021;373(6556):760-67. doi: doi:10.1126/science.abd1449.
4. Fitzgerald RC, di Pietro M, Ragunath K, et al. British Society of Gastroenterology guidelines on the diagnosis and management of Barrett's oesophagus. *Gut* 2014;63(1):7-42. doi: 10.1136/gutjnl-2013-305372.
5. Shaheen NJ, Falk GW, Iyer PG, et al. ACG Clinical Guideline: Diagnosis and Management of Barrett's Esophagus. *Am J Gastroenterol* 2016;111(1):30-50; quiz 51. doi: 10.1038/ajg.2015.322.
6. Zeki S, Fitzgerald RC. Targeting care in Barrett's oesophagus. *Clinical Medicine* 2014;14(Suppl 6):s78-s83. doi: 10.7861/clinmedicine.14-6-s78.
7. Locke GR, 3rd, Talley NJ, Fett SL, et al. Prevalence and clinical spectrum of gastroesophageal reflux: a population-based study in Olmsted County, Minnesota. *Gastroenterology* 1997;112(5):1448-56. doi: 10.1016/s0016-5085(97)70025-8.
8. National Institute for Health and Care Excellence (NICE). Gastro-oesophageal reflux disease and dyspepsia in adults: investigation and management. NICE guideline.
9. NHS England. 2018/19 National Cost Collection data. Available from: https://www.england.nhs.uk/national-cost-collection/#ncc1819 (Accessed 4th January 2021).
10. Goudra B, Nuzat A, Singh PM, et al. Association between Type of Sedation and the Adverse Events Associated with Gastrointestinal Endoscopy: An Analysis of 5 Years' Data from a Tertiary Center in the USA. *Clin Endosc* 2017;50(2):161-69. doi: 10.5946/ce.2016.019.
11. Catlow J, Beaton D, Beintaris I, et al. JAG/BSG national survey of UK endoscopy services: impact of the COVID-19 pandemic and early restoration of endoscopy services. *Frontline Gastroenterology* 2020:flgastro-2020-101582. doi: 10.1136/flgastro-2020-101582.
12. Kadri SR, Lao-Sirieix P, O’Donovan M, et al. Acceptability and accuracy of a non-endoscopic screening test for Barrett’s oesophagus in primary care: cohort study. *BMJ* 2010;341:c4372. doi: 10.1136/bmj.c4372.
13. Ross-Innes CS, Debiram-Beecham I, O'Donovan M, et al. Evaluation of a Minimally Invasive Cell Sampling Device Coupled with Assessment of Trefoil Factor 3 Expression for Diagnosing Barrett's Esophagus: A Multi-Center Case–Control Study. *PLOS Medicine* 2015;12(1):e1001780. doi: 10.1371/journal.pmed.1001780.
14. Benaglia T, Sharples LD, Fitzgerald RC, et al. Health benefits and cost effectiveness of endoscopic and nonendoscopic cytosponge screening for Barrett's esophagus. *Gastroenterology* 2013;144(1):62-73.e6. doi: 10.1053/j.gastro.2012.09.060.
15. Heberle CR, Omidvari AH, Ali A, et al. Cost Effectiveness of Screening Patients With Gastroesophageal Reflux Disease for Barrett's Esophagus With a Minimally Invasive Cell Sampling Device. *Clin Gastroenterol Hepatol* 2017;15(9):1397-404.e7. doi: 10.1016/j.cgh.2017.02.017.
16. Januszewicz W, Tan WK, Lehovsky K, et al. Safety and Acceptability of Esophageal Cytosponge Cell Collection Device in a Pooled Analysis of Data From Individual Patients. *Clin Gastroenterol Hepatol* 2019;17(4):647-56.e1. doi: 10.1016/j.cgh.2018.07.043.
17. Iqbal U, Siddique O, Ovalle A, et al. Safety and efficacy of a minimally invasive cell sampling device ('Cytosponge') in the diagnosis of esophageal pathology: a systematic review. *Eur J Gastroenterol Hepatol* 2018;30(11):1261-69. doi: 10.1097/meg.0000000000001210.

For peer review only - http://bmjopen.bmj.com/site/about/guidelines.xhtml

18. Fitzgerald RC, di Pietro M, O'Donovan M, et al. Cytosponge-trefoil factor 3 versus usual care to identify Barrett's oesophagus in a primary care setting: a multicentre, pragmatic, randomised controlled trial. *Lancet* 2020;396(10247):333-44. doi: 10.1016/s0140-6736(20)31099-0.
19. Offman J, Muldrew B, O'Donovan M, et al. Barrett's oESophagus trial 3 (BEST3): study protocol for a randomised controlled trial comparing the Cytosponge-TFF3 test with usual care to facilitate the diagnosis of oesophageal pre-cancer in primary care patients with chronic acid reflux. *BMC Cancer* 2018;18(1):784. doi: 10.1186/s12885-018-4664-3.
20. Jones R, Coyne K, Wiklund I. The gastro-oesophageal reflux disease impact scale: a patient management tool for primary care. *Aliment Pharmacol Ther* 2007;25(12):1451-9. doi: 10.1111/j.1365-2036.2007.03343.x.
21. Marteau TM, Bekker H. The development of a six-item short-form of the state scale of the Spielberger State-Trait Anxiety Inventory (STAI). *Br J Clin Psychol* 1992;31(3):301-6. doi: 10.1111/j.2044-8260.1992.tb00997.x.
22. Lerman C, Trock B, Rimer BK, et al. Psychological side effects of breast cancer screening. *Health Psychol* 1991;10(4):259-67. doi: 10.1037//0278-6133.10.4.259.
23. Schoen RE, Weissfeld JL, Bowen NJ, et al. Patient satisfaction with screening flexible sigmoidoscopy. *Arch Intern Med* 2000;160(12):1790-6. doi: 10.1001/archinte.160.12.1790.
24. Balsamo M, Cataldi F, Carlucci L, et al. Assessment of anxiety in older adults: a review of self-report measures. *Clin Interv Aging* 2018;13:573-93. doi: 10.2147/cia.S114100.
25. Stata Statistical Software: Release 15 [program]. College Station, TX: StataCorp LLC.
26. Ritchie J, Lewis C, McNaughton Nicholls C, et al. Qualitative Research Practice (Second Edition). London, UK: SAGE Publications Ltd. 2014.
27. Sekhon M, Cartwright M, Francis JJ. Acceptability of healthcare interventions: an overview of reviews and development of a theoretical framework. *BMC Health Services Research* 2017;17(1):88. doi: 10.1186/s12913-017-2031-8.
28. McBride E, Tatar O, Rosberger Z, et al. Emotional response to testing positive for human papillomavirus at cervical cancer screening: a mixed method systematic review with meta-analysis. *Health Psychol Rev* 2021;15(3):395-429. doi: 10.1080/17437199.2020.1762106.
29. Fagerlin A, Zikmund-Fisher BJ, Ubel PA. Helping patients decide: ten steps to better risk communication. *J Natl Cancer Inst* 2011;103(19):1436-43. doi: 10.1093/jnci/djr318.
30. Katzka DA, Geno DM, Ravi A, et al. Accuracy, safety, and tolerability of tissue collection by Cytosponge vs endoscopy for evaluation of eosinophilic esophagitis. *Clin Gastroenterol Hepatol* 2015;13(1):77-83.e2. doi: 10.1016/j.cgh.2014.06.026.
31. Freeman M, Offman J, Walter FM, et al. Acceptability of the Cytosponge procedure for detecting Barrett's oesophagus: a qualitative study. *BMJ Open* 2017;7(3):e013901. doi: 10.1136/bmjopen-2016-013901.
32. Whelehan P, Evans A, Wells M, et al. The effect of mammography pain on repeat participation in breast cancer screening: a systematic review. *Breast* 2013;22(4):389-94. doi: 10.1016/j.breast.2013.03.003.
33. Waller J, Bartoszek M, Marlow L, et al. Barriers to cervical cancer screening attendance in England: a population-based survey. *J Med Screen* 2009;16(4):199-204. doi: 10.1258/jms.2009.009073.

For peer review only

For peer review only - http://bmjopen.bmj.com/site/about/guidelines.xhtml

FIGURE LEGENDS

Figure 1. Summary of findings from questionnaires and patient interviews according to the themes of the Inventory to Assess Patient Satisfaction.

Figure 2. Ratings for perceived risk of oesophageal cancer at the Cytosponge appointment (baseline) and 7-14 day follow-up for participants completing both baseline and follow-up questionnaires (N = 1488). (a) Risk of oesophageal cancer compared to someone of the same age (comparative risk). (b) Per cent absolute risk of oesophageal cancer.

Possible answers to the multiple-choice question on absolute percent risk of oesophageal cancer were: 0%, 5%, 10%, 25%, 50%, 75% and 100%. Participants with missing answers on perceived risk of oesophageal cancer at follow-up were still included in the figures as they filled in other parts of the questionnaire.

For peer review only

For peer review only - http://bmjopen.bmj.com/site/about/guidelines.xhtml

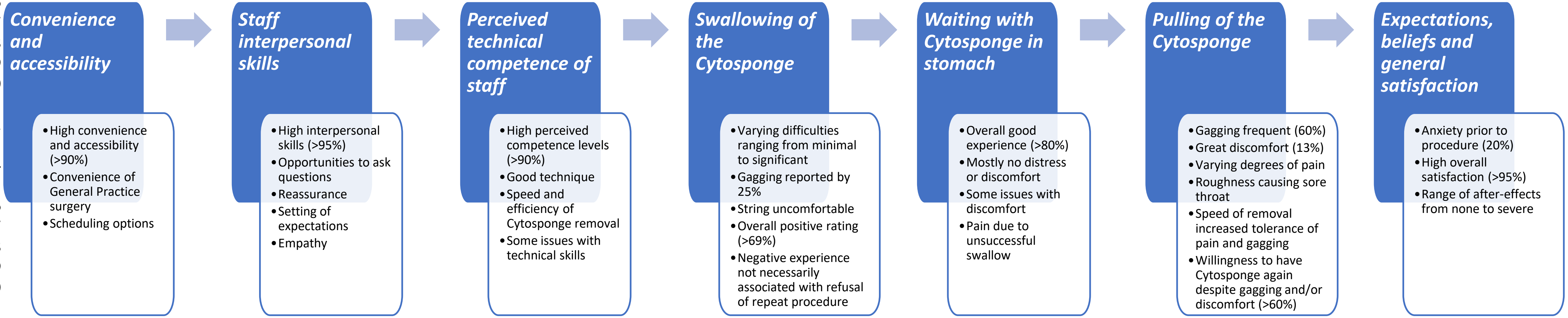
Convenience and accessibility
• High convenience and accessibility (>90%)
• Convenience of General Practice surgery
• Scheduling options
Staff interpersonal skills
• High interpersonal skills (>95%)
• Opportunities to ask questions
• Reassurance
• Setting of expectations
• Empathy
Perceived technical competence of staff
• High perceived competence levels (>90%)
• Good technique
• Speed and efficiency of Cytosponge removal
• Some issues with technical skills
Swallowing of the Cytosponge
• Varying difficulties ranging from minimal to significant
• Gagging reported by 25%
• String uncomfortable
• Overall positive rating (>69%)
• Negative experience not necessarily associated with refusal of repeat procedure
Waiting with Cytosponge in stomach
• Overall good experience (>80%)
• Mostly no distress or discomfort
• Some issues with discomfort
• Pain due to unsuccessful swallow
Pulling of the Cytosponge
• Gagging frequent (60%)
• Great discomfort (13%)
• Varying degrees of pain
• Roughness causing sore throat
• Speed of removal increased tolerance of pain and gagging
• Willingness to have Cytosponge again despite gagging and/or discomfort (>60%)
Expectations, beliefs and general satisfaction
• Anxiety prior to procedure (20%)
• High overall satisfaction (>95%)
• Range of after-effects from none to severe

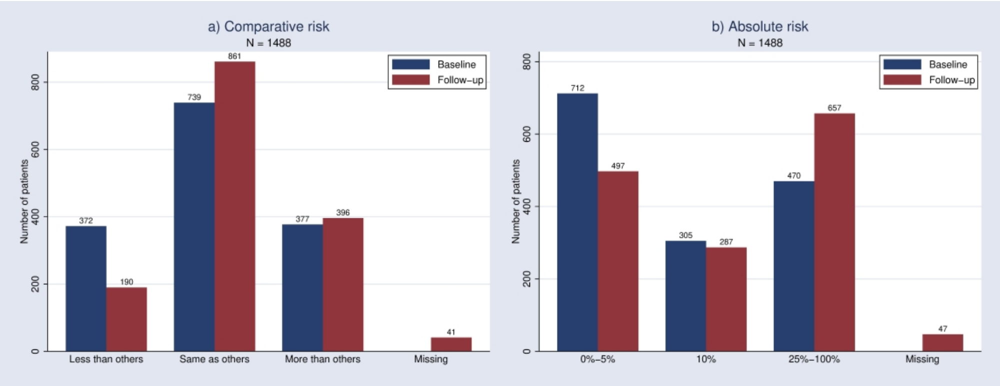

Figure 2. Ratings for perceived risk of oesophageal cancer at the Cytosponge appointment (baseline) and 7-14 day follow-up for participants completing both questionnaires (N = 1488). (a) Risk compared to someone of the same age (comparative risk). (b) Percent absolute risk.
Possible answers to the multiple-choice question on absolute percent risk were: 0%, 5%, 10%, 25%, 50%, 75% and 100%. Participants with missing answers at follow-up were included in the figures as they filled in other parts of the post-test questionnaire.

330x127mm (600 x 600 DPI)

Patient-reported experiences and views on the Cytosponge test: a mixed-methods analysis from the BEST3 trial

Roberta Maroni*, Jessica Barnes*, Judith Offman*, Fiona Scheibl, Samuel G Smith, Irene Debiram-Beecham, Jo Waller, Peter D Sasieni, Rebecca C Fitzgerald, Greg Rubin, BEST3 Consortium, Fiona M Walter

*Roberta Maroni, Jessica Barnes and Judith Offman equally contributed to this paper.

SUPPLEMENTARY MATERIALS

METHODS

Study design

The BEST3 trial [1] was a randomised controlled trial set in primary care with a mixed design (site-level and patient-level randomised) that enrolled 13,222 participants aged 50 or over with acid reflux symptoms ongoing for more than six months, identified via their general practice medical records. The primary endpoint was to compare the rate of diagnosis of Barrett's oesophagus between those offered the Cytosponge-trefoil factor 3 (TFF3) test and those on current management, and the results showed a ten-fold increase in being diagnosed with Barrett's oesophagus in the intervention arm compared with usual care.

The invitation letter (intervention arm only) was accompanied by an information leaflet on the Cytosponge. Participants expressing interest in receiving the test received a further information sheet with more details on the study and the Cytosponge. On the day of the test, participants were asked not to eat or drink anything in the four hours before the appointment. The appointment was held at participants' general practices and attendees (N = 1750) were offered an anaesthetic throat spray (optional) and water to drink to help ingest the capsule, following which 1654 (95%) patients produced a successful swallow. Furthermore, those producing a successful swallow but receiving an 'inadequate' test result (i.e. low-confidence negative TFF3, equivocal, or processing/technical failure) were invited to a repeat appointment when local resources and capacity allowed for that. All patients with a positive TFF3 result were referred for a confirmatory endoscopy, which was necessary to establish a diagnosis of Barrett's oesophagus or oesophageal cancer.

Data collection

Qualitative analysis

Face-to-face interviews were conducted by Fiona Scheibl, BA (Hons) and PhD, working at the time as Research Associate for the Department of Public Health and Primary Care at the University of Cambridge. FS has undergraduate and postgraduate training in social research and has spent more than 15 years in social and health care research in several universities in the UK. No relationship between FS and the interviewees was established prior to study commencement and the participants had no knowledge of the researcher's goals, except for what was reported in the Patient Information Leaflet, the invitation letters or the further information sheet sent by the BEST3 team, which set out all the aims and terms of the research project. The interview questions were provided by the authors and were pilot tested. No field notes were collected. Transcriptions of the audio recordings of the interviews were not returned to participants for comments or correction.

Analysis

Quantitative analysis

Questionnaire scoring

- Gastro-oesophageal reflux disease Impact Scale [2]: answers to each item were converted to scores on a four-point ordinal scale (1 = 'Never', 2 = 'Sometimes', 3 = 'Often', 4 = 'Daily') and then averaged to obtain each participant's final score.

For peer review only - http://bmjopen.bmj.com/site/about/guidelines.xhtml

- Shorter six-item form of the Spielberger State-Trait Anxiety Inventory (STAI-6) [3]: item scores on a four-point ordinal scale (1 = 'Not at all', 2 = 'Somewhat', 3 = 'Moderately', 4 = 'Very much') were reversed for positively worded questions and their sum was scaled so that the total score ranged from 20 to 80, as per the STAI guidelines.
- Perceived risk [5]: both risk compared to someone of the same age ('Much lower', 'Lower', 'Neither higher nor lower', 'Higher', 'Much higher') and absolute risk in a lifetime ('0%', '5%', '10%', '25%', '50%', '75%', '100%') are shown with some of the answer categories combined.
- Inventory to Assess Patient Satisfaction (IAPS) [6]: ratings categories ('Strongly agree', 'Agree', 'Not sure', 'Disagree', 'Strongly disagree') were combined ('Agree', 'Not sure', 'Disagree') and number and proportion of participants for each item are presented. For presentation purposes (Table 2), the text of items referring to negative aspects of the patient experience was rephrased using negative constructs to facilitate the visual comparison between items. Answers to the three items in the category "Pulling of the Cytosponge" were converted to scores on a 5-point ordinal scale (1 = 'Strongly agree', 2 = 'Agree', 3 = 'Not sure', 4 = 'Disagree', 5 = 'Strongly disagree'), which were then reversed for the two items referring to negative aspects. The three scores were then averaged for each patient to identify participants dissatisfied with the Cytosponge retrieval (i.e. score of 4 or above). Their ratings were cross-checked with the two inventory items referring to willingness to have the procedure again or to recommend it to friends.

Qualitative analysis

Researcher JB performed a thematic analysis on the interview data, with input from FW and JW. Data were organised and managed according to the Framework approach.[7] After familiarisation with the data through reading all the transcripts, JB developed an initial thematic framework of data labels. The aim with producing the initial set of labels was to enable effective data sorting and management – not to arrive at an exhaustive set of themes. This involved identifying an initial, broad set of labels that would be used to label and sort the data to enable the subsequent thematic analysis.

Labels were created inductively and deductively. Inductively-created labels were based on emergent concepts identified in the data. Deductively-created labels were based on the IAPS (as used in the quantitative questionnaire for the trial) [6] and the Theoretical Framework of Acceptability (TFA).[8] Use of the IAPS constructs as labels allowed us to more directly relate participant experience across qualitative and quantitative datasets. Use of the TFA constructs allowed us to examine additional dimensions of patient experience associated with acceptability that were not captured by IAPS.

Labels were discussed and reviewed by JB, FW and JW. JB then sorted the data by reading through each transcript and applying the labels cross-sectionally (i.e. the set of labels was applied across the entire set of transcripts where relevant). The labelled data was then transposed into the conventional Framework matrix, in which each label becomes a column and each participant/case becomes a row.

REFERENCES

1. Fitzgerald RC, di Pietro M, O'Donovan M, et al. Cytosponge-trefoil factor 3 versus usual care to identify Barrett's oesophagus in a primary care setting: a multicentre, pragmatic, randomised controlled trial. *Lancet* 2020;396(10247):333-44. doi: 10.1016/s0140-6736(20)31099-0.
2. Jones R, Coyne K, Wiklund I. The gastro-oesophageal reflux disease impact scale: a patient management tool for primary care. *Aliment Pharmacol Ther* 2007;25(12):1451-9. doi: 10.1111/j.1365-2036.2007.03343.x.
3. Marteau TM, Bekker H. The development of a six-item short-form of the state scale of the Spielberger State-Trait Anxiety Inventory (STAI). *Br J Clin Psychol* 1992;31(3):301-6. doi: 10.1111/j.2044-8260.1992.tb00997.x.
4. Kadri SR, Lao-Sirieix P, O'Donovan M, et al. Acceptability and accuracy of a non-endoscopic screening test for Barrett's oesophagus in primary care: cohort study. *BMJ* 2010;341:c4372. doi: 10.1136/bmj.c4372.
5. Lerman C, Trock B, Rimer BK, et al. Psychological side effects of breast cancer screening. *Health Psychol* 1991;10(4):259-67. doi: 10.1037//0278-6133.10.4.259.
6. Schoen RE, Weissfeld JL, Bowen NJ, et al. Patient satisfaction with screening flexible sigmoidoscopy. *Arch Intern Med* 2000;160(12):1790-6. doi: 10.1001/archinte.160.12.1790.

For peer review only - http://bmjopen.bmj.com/site/about/guidelines.xhtml

7. Ritchie J, Lewis C, McNaughton Nicholls C, et al. Qualitative Research Practice (Second Edition). London, UK: SAGE Publications Ltd. 2014.
8. Sekhon M, Cartwright M, Francis JJ. Acceptability of healthcare interventions: an overview of reviews and development of a theoretical framework. *BMC Health Services Research* 2017;17(1):88. doi: 10.1186/s12913-017-2031-8.

SUPPLEMENTARY TABLES

Supplementary Table 1. Sampling characteristics* of BEST3 participants being interviewed.

	No. of participants (N = 30)
**Geographic region in England**	
East	20
North-east	8
West	2
**Cytosponge-TFF3 outcome (at first appointment)**	
TFF3 negative	10
TFF3 positive	10
Inadequate (equivocal/low-confidence negative/technical or processing failure)**	6
Unsuccessful swallow	4
**Visual analogue scale acceptability rating (0-10)*** **	
5	2
6	1
7	1
8	4
9	4
10	9
Missing	9

*Also refer to Table 1 for the other sampling characteristics: age group and sex.
**Participants with an inadequate test result were invited to a repeat appointment when local resources and availability allowed for that.
***Visual analogue scale ratings were not used to ensure equal sampling of interviewees across scores, but rather to guarantee a diversity of experiences.

Supplementary Table 2. Understanding of test results, summarised by themes and quotes from patient interviews.

Cytosponge test result	Theme	Exemplar quotes
Positive	<u>Sense of shock due to expecting negative result</u><br>Some participants experienced shock as they had expected a negative result based on their understanding of cancer in general (i.e. that it is caused by lifestyle factors such as drinking or smoking, or that it is hereditary) rather than an understanding of Barrett's oesophagus or oesophageal cancer.	*"I never thought any further than taking the test, really. Well, I mean I don't smoke, I don't drink so I didn't expect anything other than a clear."*<br>(age 80+, positive result)
	<u>Sense of shock due to connotations of cancer more generally</u><br>Receiving a positive Cytosponge result was experienced to some degree as being like receiving a cancer diagnosis for some participants.	*"I think it was just a shock to hear that, you automatically… when I've read the leaflets and that and it's like Barrett's oesophagus is like looking for cancer, you just automatically always have that word in the back of your head, which I still have."*<br>(age 50-59, positive result)

For peer review only - http://bmjopen.bmj.com/site/about/guidelines.xhtml

	<u>Sense of confusion or concern about test result meaning</u><br>Some participants receiving positive test results felt that the use of the word "positive" was difficult to understand, as they initially interpreted it in the lay sense of meaning "good".<br>Another cause of concern was about how to communicate the positive test result to family members. Some participants did not have an adequate understanding of what the test result meant to be able to explain it reassuringly to their family members.	*"I think saying positive is like saying you've got it. It would be like a possibility that needs further investigation or something like that."*<br>(age 50-59, positive result)<br><br>*"I suppose really it may have been better for possible where you get a positive is maybe sit there in front of the GP with your wife and then explain, because I had no idea what Barrett's was and you can look it up and it tells you all sorts of... and it's not the best way to look at anything, is it?"*<br>(age 50-59, positive result)
	<u>No particularly strong reaction to positive result</u><br>Some participants did not react strongly to their result. In some cases, this was due to previous experiences that had given them relevant literacy or knowledge on cancer and cancer test results. In other cases, it was because the participant felt they had the necessary coping skills and attitudes, such as feeling there was no point in worrying, or that any problems can be managed or planned around.	*"... it didn't worry me, I had no problem. I want to know, end of, whatever you're going to throw at me, as long as I can plan it, that's how I live my life."*<br>(age 60-69, positive result)
Negative	<u>Sense of relief</u><br>The negative result alleviated a sense of uncertainty or anxiety for some participants. This sometimes extended to a sense of relief on behalf of their families. In some cases, participants felt relieved to get confirmation that they were not on the same trajectory as family members who had previously suffered from issues related to reflux, or to get confirmation that their PPI medication had been effective.	Researcher: *Alright then, and did you have any emotional feelings about having that result at all, apart from relief?*<br>Participant: *No, just relief really. And the family as well.*<br>Interviewer: *Yeah.*<br>Participant: *Because I got to that stage now where my children think they should look after me, so it was a relief to them as well.*<br>(age 70-79, negative result)
	<u>No particularly significant reaction, or a mildly positive reaction</u><br>This was sometimes due to participants simply having expected a negative result, while others simply had the attitude that there was no point in worrying.	*"I wasn't particularly bothered. There would be either something wrong or not"*<br>(age 70-79, negative result)
Inadequate (low confidence/ equivocal/ processing failure)	<u>Understood the result</u><br>Some participants understood what this result meant and the reasons behind it and were willing to attend a second appointment for another procedure. They reported understanding that the reason for their result was that there were not enough cells collected.	*"The first time I didn't receive any notification. It was about two...just over two weeks, but that possibly was to do with the fact that they hadn't been able to take enough cells, and that notification was just to say that... apologising there wasn't enough cells, and would I mind coming back? And I said no, it's fine. And the second time I received a letter quite quickly, about seven to ten days afterwards, saying that the cells were all normal."*<br>(age 60-69, inadequate result)

For peer review only - http://bmjopen.bmj.com/site/about/guidelines.xhtml

	A sense of confusion about what the result meant<br>Some participants seemed unaware that this result was possible. They wondered why the test had not worked as expected and had not collected enough cells, and if theirs was the only case of this occurring. In some cases, this experience generated mistrust. This suggests that participants needed clearer information about how this result might come about and how common it is.	*"Well it did cross my mind that I wasn't being told the truth the first time. [...] I just wondered about it. [...] But I was assured that wasn't the case, I wasn't told anything that wasn't the truth. I was told there were too few cells collected."*<br>(age 70-79, inadequate result from first test, negative result from second test)

Supplementary Box 1. Types of discomfort during removal of the Cytosponge reported by patients being interviewed.

"Notable" pain, pain that was "horrible" or "worse than was expecting"
However, in these instances, some participants noted that the pain was to be expected (and interpreted the pain as a sign that the Cytosponge was effectively gathering cells). Alternatively, some participants felt the pain was so brief that it was not a problem. In these cases where pain was experienced, participants also said they would still be willing to have the Cytosponge procedure again, suggesting that an understanding of the mechanisms of the Cytosponge and the speed of its removal were factors that made the pain more tolerable. This was also confirmed by responses to the IAPS questionnaire: out of the 193 participants agreeing with the statement "Pulling up of the Cytosponge™ caused me great discomfort" (Table 2), 113 (59%) said that they would be willing to have another test if necessary.

Gagging
Some participants mentioned that this was unpleasant but also that, because the removal process was so fast that the gagging was not much of a problem, they would be willing to have the Cytosponge procedure again. Again, this was confirmed by responses to the IAPS questionnaire: out of the 889 participants agreeing with the statement "I had to gag when the Cytosponge™ was pulled up" (Table 2), 688 (79%) said they would be willing to have another test if necessary.

Roughness or scratching from the Cytosponge
In some cases, this caused the throat to immediately feel sore. One participant commented, however, that they understood the need for the Cytosponge to be rough, otherwise it would not effectively gather cells.

Weird or unexpected sensations
For example, the removal felt funny, weird, disconcerting, strange, or "that you can literally feel it being pulled like it could cut the back of your tongue. But I don't think it actually did because I had no pain afterwards so I was fine" [age 60-69, negative result].

Supplementary Table 3. Overall STAI-6 score at Cytosponge-TFF3 appointment (baseline) and 7-14 day follow-up for participants completing both questionnaires.

**Overall STAI-6 score**		**At follow-up**				
		20<40	40<60	60-80	Missing	**Total**
**At baseline**	20<40	858	82	2	48	**990**
	40<60	300	142	12	21	**475**
	60-80	7	13	2	1	**23**
	**Total**	**1165**	**237**	**16**	**70**	**1488**

For peer review only - http://bmjopen.bmj.com/site/about/guidelines.xhtml

Supplementary Table 4. Perceived risk of oesophageal cancer compared to someone of the same age at Cytosponge-TFF3 appointment (baseline) and 7-14 day follow-up for participants completing both questionnaires.

**Relative risk of oesophageal cancer**		**At follow-up**					**p-value for McNemar's test (comparing "less than others" vs "more than others")**
		Less than others	Same as others	More than others	Missing	**Total**	
**At baseline**	Less than others	130	189	36	17	**372**	< 0.001
	Same as others	56	570	94	19	**739**	
	More than others	4	102	266	5	**377**	
	**Total**	**190**	**861**	**396**	**41**	**1488**	

Supplementary Table 5. Perceived percent risk of developing oesophageal cancer in a lifetime at Cytosponge-TFF3 appointment (baseline) and 7-14 day follow-up for participants completing both questionnaires.

**Percent absolute risk of oesophageal cancer**		**At follow-up**					**p-value for McNemar's test (comparing 0%, 5% vs 25%, 50%, 75%, 100%)**
		0%, 5%	10%	25%, 50%, 75%, 100%	Missing	**Total**	
**At baseline**	0%, 5%	396	108	181	27	**712**	< 0.001
	10%	70	123	100	12	**305**	
	25%, 50%, 75%, 100%	31	56	375	8	**470**	
	Missing	0	0	1	0	**1**	
	**Total**	**497**	**287**	**657**	**47**	**1488**	

For peer review only

SUPPLEMENTARY FIGURES

Supplementary Figure 1. (a) Trial flowchart for the patient-reported experience analysis of the BEST3 trial. (b) Venn diagram with the three subgroups of participation outlined in the analysis.

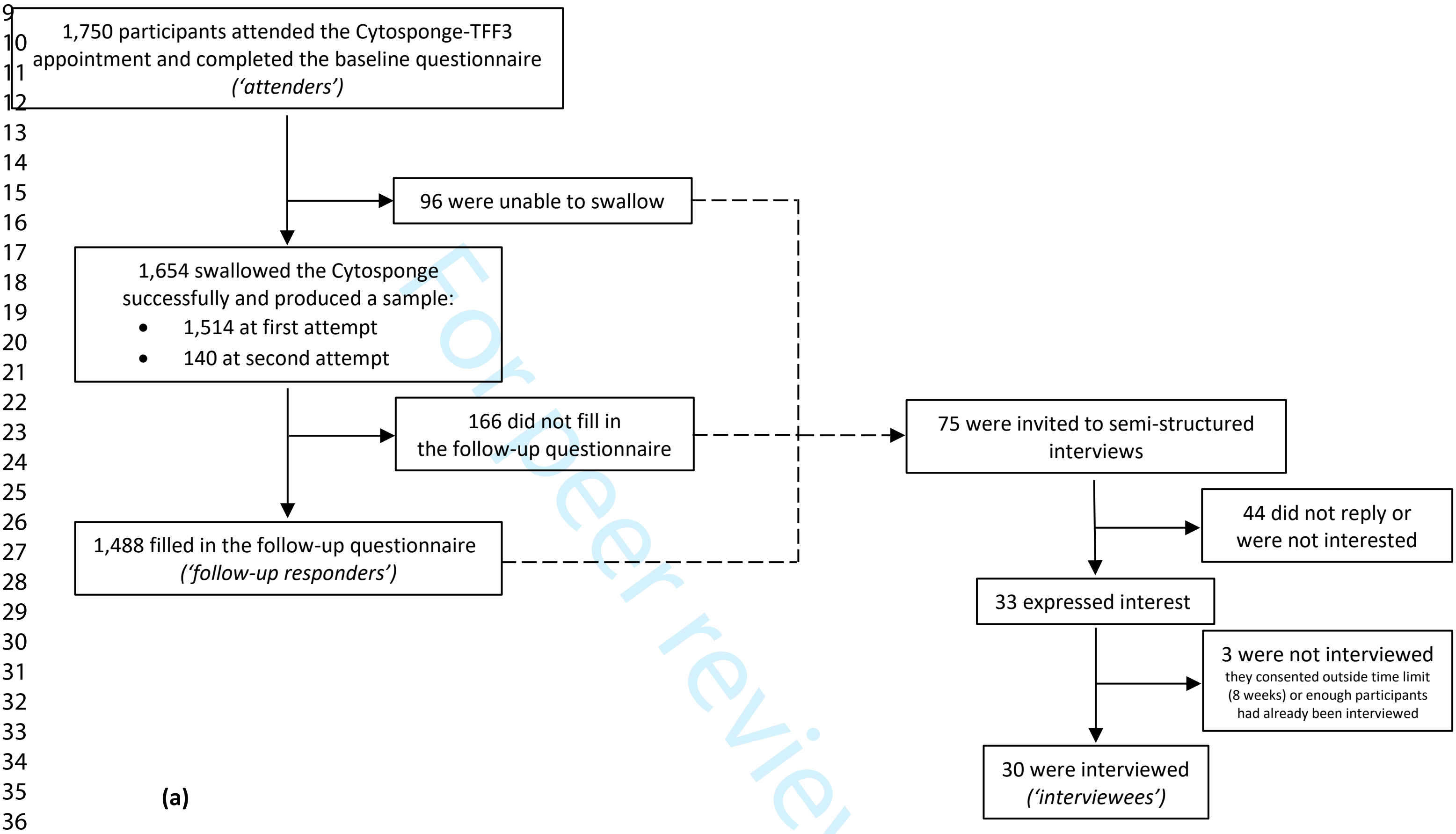

(a)

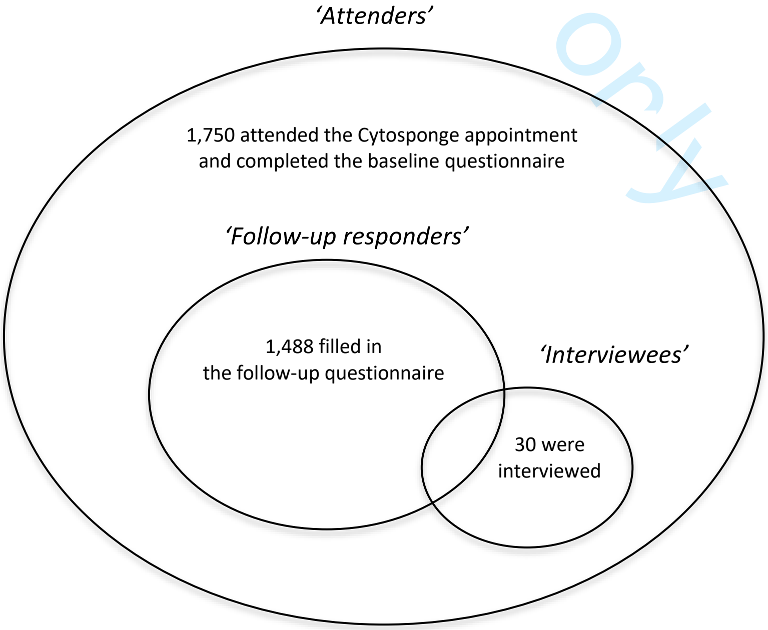

(b)

For peer review only - http://bmjopen.bmj.com/site/about/guidelines.xhtml

APPENDIX - QUESTIONNAIRES

A. BASELINE CLINICAL FORM

GENERAL INFORMATION

1. Height .... cm
2. Weight .... kg
3. What was your weight aged 20?
   - Underweight
   - Normal range
   - Overweight
   - Obese
   - Don't remember

4. Have you ever been obese?
   - Yes
   - No

5. Waist circumference .... cm
6. Hip circumference .... cm

MEDICATION

7. Medication type for reflux symptoms
   - H2 receptor antagonists
   - Proton pump inhibitor
   - Over the counter anti-acids
   - Other

7a. Name: ....

7b. Dose: ....

7c. Units:
- mg
- g
- ml
- tablet

7d. Frequency:
- OD
- BD
- TDS
- QDS
- PRN

7e. Month/year started:
- 0-1 year
- 1-2 years
- 3-4 years
- 4-5 years
- 5-6 years
- 6+ years

8. Please confirm whether patient has any comorbidities:
   - Yes
   - No

[COMORBIDITY TYPES COLLECTED IN SEPARATE SUBFORM]

SYMPTOMS

9. Gastro-oesophageal Reflux Disease Impact Scale (GIS) – part A

Please complete the following questions by marking one response per question. Consider your symptoms prior to you taking any acid suppressant medication. There are no right or wrong answers. Be sure to answer every question.

Prior to you taking any acid suppressant medication please confirm the following…	DAILY	OFTEN	SOMETIMES	NEVER
1. How often did you have the following symptoms:				
a. Pain in your chest or behind the breastbone?				
b. Burning sensation in your chest or behind the breastbone?				
c. Regurgitation or acid taste in your mouth?				
d. Pain or burning in your upper stomach?				
e. Sore throat or hoarseness that was related to your heartburn or acid reflux?				
2. How often did you have difficulty getting a good night's sleep because of your symptoms?				
3. How often did your symptoms prevent you from eating or drinking any of the foods you like?				
4. How frequently did your symptoms keep you from being fully productive in your job or daily activities?				

For peer review only - http://bmjopen.bmj.com/site/about/guidelines.xhtml

5. How often did you buy over-the-counter medication (such as Rennies, Tums, Gaviscon)?				

10. Gastro-oesophageal Reflux Disease Impact Scale (GIS) – part B

Please complete the following questions by marking one response per question. Consider your symptoms over the past week. There are no right or wrong answers. Be sure to answer every question.

In the past week...	DAILY	OFTEN	SOMETIMES	NEVER
1. How often have you had the following symptoms:				
f. Pain in your chest or behind the breastbone?				
g. Burning sensation in your chest or behind the breastbone?				
h. Regurgitation or acid taste in your mouth?				
i. Pain or burning in your upper stomach?				
j. Sore throat or hoarseness that is related to your heartburn or acid reflux?				
2. How often have you had difficulty getting a good night's sleep because of your symptoms?				
3. How often have your symptoms prevented you from eating or drinking any of the foods you like?				
4. How frequently have your symptoms kept your from being fully productive in your job or daily activities?				
5. How often do you take additional medication other than what the physician told you to take (such as Rennies, Tums, Gaviscon)?				

For peer review only

FURTHER INFORMATION

11. How long ago did your heartburn first begin?
- Never
- Last 6 months
- 7 months to 1 year
- 1 to 2 years
- 2 to 5 years
- 5 to 10 years
- 10 to 20 years
- More than 20 years

12. How long ago did you first notice the acid/sour taste in your mouth?
- Never
- Last 6 months
- 7 months to 1 year
- 1 to 2 years
- 2 to 5 years
- 5 to 10 years
- 10 to 20 years
- More than 20 years

13. Have you been prescribed treatment for H.pylori?
- Yes
- No
- Don't know

14. Did the treatment for H.pylori make your symptoms:
- Worse
- No change
- Better

15. Are you taking medicine for your stomach symptoms?
- Yes
- No

For peer review only - http://bmjopen.bmj.com/site/about/guidelines.xhtml

B. BASELINE CLINICAL FORM

Lifestyle/family history

EDUCATION

1. What is the highest level of education that you have achieved?
   - School up to 15-16 years
   - College or vocational study
   - University graduate
   - Professional training beyond college or postgraduate degree
   - Other
   - Prefer not to say
2. If other, please specify ....

SMOKING

3. How many hours a day are you exposed to other people's smoke?
   - 0 hours
   - 1-6 hours
   - 6-12 hours
   - 12-18 hours
   - 18-24 hours
4. Have you ever smoked cigarettes, tobacco, pipe or cigars?
   - Yes
   - No
5. Age when you started smoking ....
6. Have you stopped smoking?
   - Yes
   - No
7. If you are no longer smoking, at what age did you stop? ....

How many/much did you or do you smoke per day of:

8. Cigarettes ....
9. Cigars ....
10. Tobacco (cigarettes/pipe) .... oz or grams

ALCOHOL HISTORY

11. Which one of the following best describes your present alcohol intake?
    - None
    - Daily or most days
    - Weekends only
    - Occasional (once / twice per month)
12. Which of the following is your preferred beverage(s)?
    - Red wine
    - White wine
    - Spirits
    - Beer
    - Alcopops
    - Other
    - Prefer not to say
13. If other, please specify ....
14. At present, how many units do you drink a week?
    - 1-5 units
    - 6-10 units
    - 11-15 units
    - 16-20 units
    - 21-25 units
    - 26-30 units
    - 30+ units
    - Not sure
    - Prefer not to say
15. Did you ever drink heavily in the past? (Heavy drinking is defined as >14 units per week for women and >21 units per week for men)
    - Yes
    - No
    - Not sure
    - Prefer not to say
16. How many units a week did you drink when you were 20?
    - 0 units
    - 1-5 units
    - 6-10 units
    - 11-15 units
    - 16-20 units
    - 21-25 units
    - 26-30 units
    - 30+ units
    - Not sure
    - Prefer not to say

For peer review only

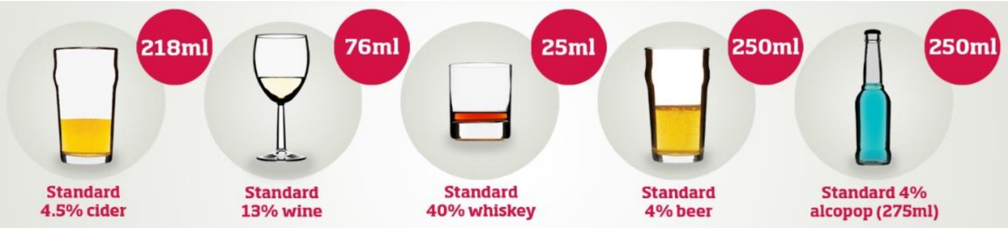

Courtesy of Public Health England

FAMILY HISTORY

17. Do any of your family have any of the following: heartburn, Barretts's oesophagus, cancer of the gullet/oesophagus, any other cancer and type.
   - Yes
   - No

18. (Please answer all questions for the relatives this is applicable for)

Relative	Heartburn	Barrett's oesophagus	Cancer of the gullet or oesophagus	Any other cancer and type

Perceived risk of developing oesophageal cancer

These questions are about how susceptible you feel to oesophageal cancer.

Compared to a person of the same age as you, what are your chances of developing oesophageal cancer? *(Please tick one)*	Much lower ☐<br>Lower ☐<br>Neither higher nor lower ☐<br>Higher ☐<br>Much higher ☐
In your lifetime, what do you consider your risk of developing oesophageal cancer is? *(Please tick one)*	0% ☐<br>5% ☐<br>10% ☐<br>25% ☐<br>50% ☐<br>75% ☐<br>100% ☐

Short-form of the state scale of the Spielberger State-Trait Anxiety Inventory (STAI-6)

A number of statements which people have used to describe themselves are given below. Read each sentence and then circle the most appropriate number to the right of the statement to indicate how you feel RIGHT NOW, AT THIS MOMENT. There are no right or wrong answers. Do not spend too much time on any one statement but give the answer which seems to describe your present feelings best.

(Please tick one box for each statement)

	Not at all	Somewhat	Moderately	Very much
**I feel calm**	1	2	3	4
**I am tense**	1	2	3	4
**I feel upset**	1	2	3	4
**I am relaxed**	1	2	3	4
**I feel content**	1	2	3	4
**I am worried**	1	2	3	4

For peer review only

For peer review only - http://bmjopen.bmj.com/site/about/guidelines.xhtml

C. 7-14 DAY FOLLOW-UP QUESTIONNAIRE

Inventory to Assess Patient Satisfaction (IAPS)

You recently received the Cytosponge™ test at your practice as part of the BEST3 Trial. On a scale of 1-5, please indicate whether you agree or disagree with the following statements:

(Please circle one response per statement)

	Strongly agree	Agree	Not sure	Disagree	Strongly disagree
**Convenience and accessibility**					
I felt that i had to wait too long.	1	2	3	4	5
The test is in a place that is easy for me to get to.	1	2	3	4	5
I found it hard to find a convenient time to come to the test.	1	2	3	4	5
**Staff interpersonal skills**					
I felt free to ask the staff questions i wanted to ask.	1	2	3	4	5
The staff seemed to hurry me through too quickly.	1	2	3	4	5
The staff used words that were hard to understand.	1	2	3	4	5
**Perceived technical competence**					
The nurse or member of staff was too rough when performing the Cytosponge test.	1	2	3	4	5
I feel confident that the Cytosponge test was performed properly.	1	2	3	4	5
**Swallowing of the capsule**					
I had to gag when I swallowed the Cytosponge capsule.	1	2	3	4	5
Swallowing the Cytosponge capsule was more comfortable than i expected.	1	2	3	4	5
Swallowing the Cytosponge capsule caused me great discomfort.	1	2	3	4	5
**Waiting with capsule in stomach**					
I had to gag while I waited with the Cytosponge capsule in my stomach.	1	2	3	4	5
Waiting with the Cytosponge capsule in my stomach was more comfortable than i expected.	1	2	3	4	5
Waiting with the Cytosponge capsule in my stomach caused me great discomfort.	1	2	3	4	5
**Pulling up of the Cytosponge**					
I had to gag when the Cytosponge was pulled up.	1	2	3	4	5
Pulling up of the Cytosponge was more comfortable than i expected.	1	2	3	4	5
Pulling up of the Cytosponge caused me great discomfort.	1	2	3	4	5
**Expectations and beliefs**					
I was very anxious about having the Cytosponge test.	1	2	3	4	5
Undergoing the Cytosponge test will benefit my health.	1	2	3	4	5
**General satisfaction**					
I was very satisfied with the care I received.	1	2	3	4	5
I would recommend the Cytosponge test to my friends.	1	2	3	4	5
I would be willing to have another if necessary.*	1	2	3	4	5

*As part of the Trial, you may still be invited for a repeat Cytosponge test.

For peer review only - http://bmjopen.bmj.com/site/about/guidelines.xhtml

Cytosponge test experience questionnaire

Please mark on the line your experience with the Cytosponge test:

0	5	10
Completely unacceptable	*Neither unacceptable nor acceptable*	*Completely acceptable*

Comments:

Perceived risk of developing oesophageal cancer

These questions are about how susceptible you feel to oesophageal cancer.

Compared to a person of the same age as you, what are your chances of developing oesophageal cancer? *(please tick one)*	Much lower ☐<br>Lower ☐<br>Neither higher nor lower ☐<br>Higher ☐<br>Much higher ☐
In your lifetime, what do you consider your risk of developing oesophageal cancer is? *(please tick one)*	0% ☐<br>5% ☐<br>10% ☐<br>25% ☐<br>50% ☐<br>75% ☐<br>100% ☐

Short-form of the state scale of the Spielberger State-Trait Anxiety Inventory (STAI-6)

A number of statements which people have used to describe themselves are given below. Read each sentence and then circle the most appropriate number to the right of the statement to indicate how you feel RIGHT NOW, AT THIS MOMENT. There are no right or wrong answers. Do not spend too much time on any one statement but give the answer which seems to describe your present feelings best.

(Please tick one box for each statement)

	**Not at all**	**Somewhat**	**Moderately**	**Very much**
I feel calm	1	2	3	4
I am tense	1	2	3	4
I feel upset	1	2	3	4
I am relaxed	1	2	3	4
I feel content	1	2	3	4
I am worried	1	2	3	4

For peer review only - http://bmjopen.bmj.com/site/about/guidelines.xhtml

COREQ (COnsolidated criteria for REporting Qualitative research) Checklist

A checklist of items that should be included in reports of qualitative research. You must report the page number in your manuscript where you consider each of the items listed in this checklist. If you have not included this information, either revise your manuscript accordingly before submitting or note N/A.

Topic	Item No.	Guide Questions/Description	Reported on Page No.
**Domain 1: Research team and reflexivity**			
Personal characteristics			
Interviewer/facilitator	1	Which author/s conducted the interview or focus group?	
Credentials	2	What were the researcher's credentials? E.g. PhD, MD	
Occupation	3	What was their occupation at the time of the study?	
Gender	4	Was the researcher male or female?	
Experience and training	5	What experience or training did the researcher have?	
Relationship with participants			
Relationship established	6	Was a relationship established prior to study commencement?	
Participant knowledge of the interviewer	7	What did the participants know about the researcher? e.g. personal goals, reasons for doing the research	
Interviewer characteristics	8	What characteristics were reported about the inter viewer/facilitator? e.g. Bias, assumptions, reasons and interests in the research topic	
**Domain 2: Study design**			
Theoretical framework			
Methodological orientation and Theory	9	What methodological orientation was stated to underpin the study? e.g. grounded theory, discourse analysis, ethnography, phenomenology, content analysis	
Participant selection			
Sampling	10	How were participants selected? e.g. purposive, convenience, consecutive, snowball	
Method of approach	11	How were participants approached? e.g. face-to-face, telephone, mail, email	
Sample size	12	How many participants were in the study?	
Non-participation	13	How many people refused to participate or dropped out? Reasons?	
Setting			
Setting of data collection	14	Where was the data collected? e.g. home, clinic, workplace	
Presence of non-participants	15	Was anyone else present besides the participants and researchers?	
Description of sample	16	What are the important characteristics of the sample? e.g. demographic data, date	
Data collection			
Interview guide	17	Were questions, prompts, guides provided by the authors? Was it pilot tested?	
Repeat interviews	18	Were repeat inter views carried out? If yes, how many?	
Audio/visual recording	19	Did the research use audio or visual recording to collect the data?	
Field notes	20	Were field notes made during and/or after the inter view or focus group?	
Duration	21	What was the duration of the inter views or focus group?	
Data saturation	22	Was data saturation discussed?	
Transcripts returned	23	Were transcripts returned to participants for comment and/or	

For peer review only

Topic	Item No.	Guide Questions/Description	Reported on Page No.
		correction?	
**Domain 3: analysis and findings**			
Data analysis			
Number of data coders	24	How many data coders coded the data?	
Description of the coding tree	25	Did authors provide a description of the coding tree?	
Derivation of themes	26	Were themes identified in advance or derived from the data?	
Software	27	What software, if applicable, was used to manage the data?	
Participant checking	28	Did participants provide feedback on the findings?	
Reporting			
Quotations presented	29	Were participant quotations presented to illustrate the themes/findings? Was each quotation identified? e.g. participant number	
Data and findings consistent	30	Was there consistency between the data presented and the findings?	
Clarity of major themes	31	Were major themes clearly presented in the findings?	
Clarity of minor themes	32	Is there a description of diverse cases or discussion of minor themes?	

Developed from: Tong A, Sainsbury P, Craig J. Consolidated criteria for reporting qualitative research (COREQ): a 32-item checklist for interviews and focus groups. *International Journal for Quality in Health Care*. 2007. Volume 19, Number 6: pp. 349 – 357

Once you have completed this checklist, please save a copy and upload it as part of your submission. DO NOT include this checklist as part of the main manuscript document. It must be uploaded as a separate file.

For peer review only
